# Structural and expression analysis of polyphenol oxidases potentially involved in globe artichoke (C. *cardunculus* var. *scolymus* L.) tissue browning

**DOI:** 10.1038/s41598-023-38874-4

**Published:** 2023-07-29

**Authors:** Valerio Pompili, Elena Mazzocchi, Andrea Moglia, Alberto Acquadro, Cinzia Comino, Giuseppe Leonardo Rotino, Sergio Lanteri

**Affiliations:** 1 Dipartimento di Scienze Agrarie, Forestali e Alimentari (DISAFA), Università degli Studi di Torino, Largo Paolo Braccini 2, 10095 Grugliasco, Torino Italy; 2 CREA, Research Centre for Genomics and Bioinformatics, 26836 Montanaso Lombardo, Lodi Italy

**Keywords:** Plant biotechnology, Plant genetics

## Abstract

Globe artichoke capitula are susceptible to browning due to oxidation of phenols caused by the activity of polyphenol oxidases (PPOs), this reduces their suitability for fresh or processed uses. A genome-wide analysis of the globe artichoke *PPO* gene family was performed. Bioinformatics analyses identified eleven *PPOs* and their genomic and amino acidic features were annotated. *Cis*-acting element analysis identified a gene regulatory and functional profile associated to plant growth and development as well as stress response. For some PPOs, phylogenetic analyses revealed a structural and functional conservation with different *Asteraceae* PPOs, while the allelic variants of the eleven *PPOs* investigated across four globe artichoke varietal types identified several SNP/Indel variants, some of which having impact on gene translation. By RTqPCR were assessed the expression patterns of *PPOs* in plant tissues and in vitro calli characterized by different morphologies. Heterogeneous *PPO* expression profiles were observed and three of them (*PPO6*, 7 and 11) showed a significant increase of transcripts in capitula tissues after cutting. Analogously, the same three *PPOs* were significantly up-regulated in calli showing a brown phenotype due to oxidation of phenols. Our results lay the foundations for a future application of gene editing aimed at disabling the three *PPOs* putatively involved in capitula browning.

## Introduction

Globe artichoke (*Cynara cardunculus* var. *scolymus* L.; 2n = 2x = 34) is a perennial and cross-pollinated vegetable native to the Mediterranean basin belonging to the Asteraceae family^1^. Its worldwide production has been stable over the last ten years and estimated around 1.5 million tons per year with a gross production value of approximatively 1 billion US dollars^2^. Italy harbors the richest globe artichoke primary gene pool and it is the leading producing country (around 370 million tons), indeed globe artichoke cultivation has spread to other Mediterranean countries and in recent years to the Americas and China^3^. The globe artichoke prime product is the immature inflorescence (head or capitulum), which represents up to 20% of the aboveground biomass, and it is consumed either fresh, preserved or frozen^4^. All the plants’ tissues are rich in pharmaceutically and nutraceutically active compounds, such as chlorogenic acid, cynarin (1,3-dicaffeoylquinic acid) and sesquiterpene lactones^5–7^, which have various industrial, pharmaceutical and cosmetic applications^8^. Furthermore, the plant lignocellulosic biomass has potential to be used as raw material to generate biofuels^9^.

The polyphenols present in the capitula are important constituents of the human diet acting as free radical scavengers, but they also represent the substrate for unwanted oxidative browning reactions mediated by polyphenol oxidases (PPOs). The latter are copper-containing enzymes, whose physiological role is not fully clarified, although a defence role against pathogens, pests and abiotic stressors has been postulated due to their localized activity in response to cutting and wounding^10–12^. PPOs may use monophenols and/or o-diphenols as substrates. The monophenols are at first o-hydroxylated to o-diphenols which in turn are oxidated to quinones. The latter are then polymerized producing dark pigments causing tissue browning of cut or bruised capitula following industrial processing or fresh consumption^12^, as they cause cellular disruption and make it possible the PPOs, sequestered in the plastid, to come into contact with their substrates. The tissue browning has a visual negative effect in addition to influencing flavor, nutritional properties and shelf life of capitula^13,14^.

In order to inhibit the enzymatic browning and maintain the capitula high quality after processing and during marketing, a number of techniques have been applied. They include both physical and chemical treatments such as heating, blanching, immersion in sugar or salt solutions, application of antioxidants, chelating agents or natural extracts^15–20^. However, these treatments just slow down the onset of browning and may have negative effects on flavor, texture and color of the heads.

In the last decade, the use of targeted genetic engineering to inactivate *PPOs* and reduce/avoid the oxidation of phenols has emerged as an efficient strategy to overcome the need of physical or chemical treatments, and examples have been reported in species of agronomic interest. In Artic® apple^21^ a RNA interference approach, based on the use of a chimeric *PPO* gene targeted to disrupt the expression of *MdPPO2*, was found to prevent unsightly browning in fruit flesh and offering fruit freshness longer. In potato, by targeting different combinations of *StuPPOs *via micro RNAs^22^ and CRISPR/Cas9 technologies^23^ a marked reduction of the enzymatic browning was observed when *StuPPO1-4* were inactivated in tandem. A CRISPR/Cas9-mediated *PPO* gene knock-out was also obtained in eggplant, in which the frame shifting and early-termination mutations of *SmelPPO4-6* were associated with a reduced PPO activity and browning of the berry flesh after cutting^11^. A further successful example of the CRISPR/Cas9 system application has been reported in the common mushroom (*Agaricus bisporus*), since the knocking-out of one of the six *PPO* genes caused about 30% of reduction in enzymatic browning^24^.

The knockout of *PPO* genes based on gene editing may thus represent a promising strategy to avoid the browning of globe artichoke capitula, while preserving their content in phenolic compounds. For this purpose, it is necessary to perform a complete characterization of globe artichoke members of the PPO gene family, whose number varies among species. Here, taking advantage of the recent availability of a high-quality globe artichoke genome sequence^25,26^ and the resequencing of four varietal types^27^, we report on the identification of globe artichoke *PPOs* and their corresponding promoter sequences to gain information of their genomic features as well as allele variations. Furthermore, by RTqPCR, we investigated the expression of *PPO* genes in different plant tissues, and in capitula tissues also after cutting. *PPOs*’ expression level was also evaluated in three types of in vitro growing calli in order to assess the role of the enzymes on the callus browning which is considered one of the main factors limiting artichoke plant regeneration.

## Results

### Genomics of globe artichoke *PPO* genes and phylogenetic analysis

**Figure 1:**
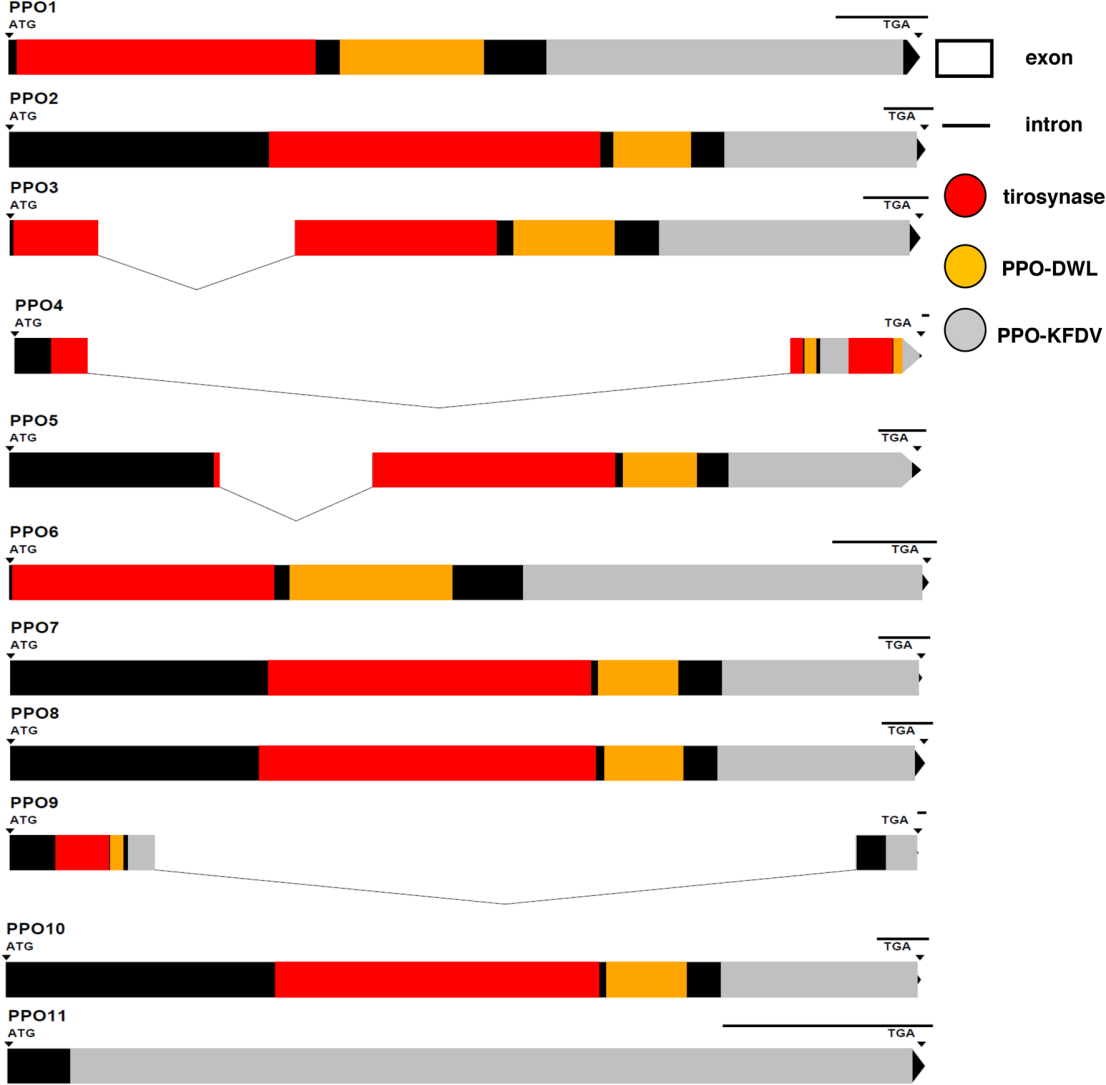
Schematic representation of *PPO* genes. The *PPO* genomic sequences are illustrated from the start codon (ATG) to the stop codon (TGA) including exon (boxes) and intron (lines) regions, the latters identified by Wormweb software (https://wormweb.org/exonintron). On the genomic sequence, the corresponding putative protein domains codified, predicted by Pfam software (https://pfam.xfam.org/), are indicated in red (tirosynase), yellow (PPO-DWL) and grey (PPO-KFDV). At the end of sequences, black unit bars indicate 100 bp.

By performing a BLASTp analysis, based on the previously annotated globe artichoke *PPOs*^25^, 11 *PPO* genes were identified in the new (v2.0) globe artichoke reference genome^26^. Genomic and putative protein features of the identified *PPOs* are summarized in Supplementary Data S1, while *PPOs* genomic sequences with corresponding putative protein existing domains are graphically illustrated in Fig. 1.

*PPO* genes were found localized on different chromosomes and on different strands (Supplementary Data S1). In details, *PPO1*-*5* mapped on different strands on chromosome 2, *PPO6* and *PPO7* mapped on the plus strand on chromosome 8 and on the minus strand on chromosome 12, respectively, while *PPO8*-*10* mapped on different strands on chromosome 17. Differently, *PPO11* localized on the plus strand of an unplaced scaffold (ScYrq3g_1694), thus its exact position in the genome is still unknown. *PPO* gene sequences were highly variable in length (Supplementary Data S1), ranging from 437 bp (of *PPO11*) to 12,813 bp (of *PPO4*). Only *PPO3*, *PPO4*, *PPO5* and *PPO9* showed the presence of one intron in the sequence, while all the other *PPOs* were exclusively characterized by exon sequences. The alignment of all the *PPO* CDSs highlighted a high nucleotide similarity between *PPO1* and *PPO2* (98.3%), *PPO2* and *PPO3* (97.5%), and *PPO1* and *PPO3* (96%).

Putative PPO proteins ranged in size from 145 (*PPO11*) to 956 (*PPO4*) amino acids (Supplementary Data S1) and were characterized by the same protein functional domains: a common central domain of tyrosinase, a polyphenol oxidase middle domain (PPO1_DWL) and a domain of unknown function (DUF_B2219) containing the KFDV conserved motif (Fig. 1; Supplementary Data S1). Interestingly, *PPO11* showed only the PPO-KFDV domain (Fig. 1; Supplementary Data S1).

**Figure 2:**
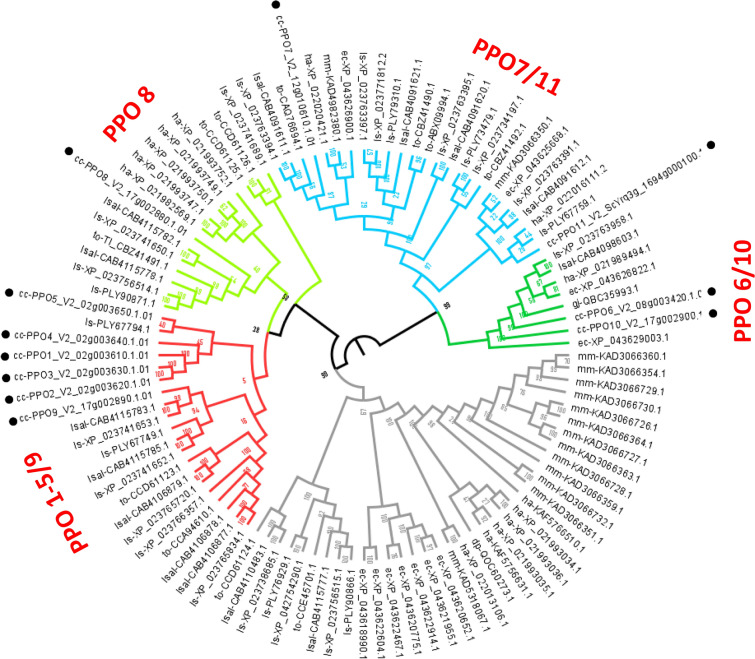
Phylogenetic tree of PPOs. The phylogenetic tree was constructed with the 11 isolated globe artichoke PPOs and a set of PPOs retrieved from 8 species of *Asteraceae*, by applying a Neighbor joining algorithm with a 1000 iterations bootstrap analysis. The generated clusters are highlighted by different colours and PPOs from *C. cardunculus* var. *scolymus* are indicated by bullet points. *Taraxacum officinale* (to)*, Lactuca sativa* (ls)*, Heliantus annuus* (ha)*, Gerbera jamesonii* (gj)*, Cynara cardunculus* var. *scolymus* (cc)*, Dahlia pinnata* (dp)*, Mikania micrantha* (mm)*, Lactuca saligna* (lsal)*, Erigeron canadensis* (ec).

A phylogenetic analysis was performed to deepen the structural features of globe artichoke PPOs. A neighbor-joining (NJ) tree (Fig. 2) was constructed, based on the 11 isolated globe artichoke PPOs and a set of PPOs (Supplementary Data S2), partially characterized, retrieved from eight species of *Asteraceae*. The NJ identified five main PPO clades, tagged on the basis of globe artichoke PPO information. One clade contained from CcPPO1 to CcPPO5 and CcPPO9; a second clade only CcPPO8; a third clade from CcPPO7 to CcPPO11; a fourth clade CcPPO6 and CcPPO10, while no CcPPOs were included in a fifth clade. A high sequence similarity was observed among the five CcPPOs (PPO1-PPO5) included in the same clade, while CcPPO9 was more distantly related. In the same clade clustered also seven *Lactuca saligna* and five *L. sativa* PPOs orthologs due to the proximity of these species at genomic level^25^. The CcPPO8, clustering separately from all the other CcPPOs, grouped with lettuce PPOs and PPOs from *Taraxacum officinale* (Fig. 2). The CcPPO7 and CcPPO11 clustered together with members of PPOs belonging to *T. officinale*, *Erigeron canadensis*, *Gerbera jamesonii*, *Mikania micrantha, L. sativa, L. saligna* and *Heliantus annuus*; CcPPO6 and CcPPO10 were also found in the same clade together with members of PPOs belonging to *E. canadensis*, *G. jamesonii*, *L. sativa, L. saligna* and *H. annuus*. At last the cluster not including CcPPOs grouped orthologs of other 6 species and included all the PPOs from *M. micrantha*, a widespread weed growing in the tropics.

### SNPs and Indel variations in *PPOs* among globe artichoke genotypes

**Figure 3:**
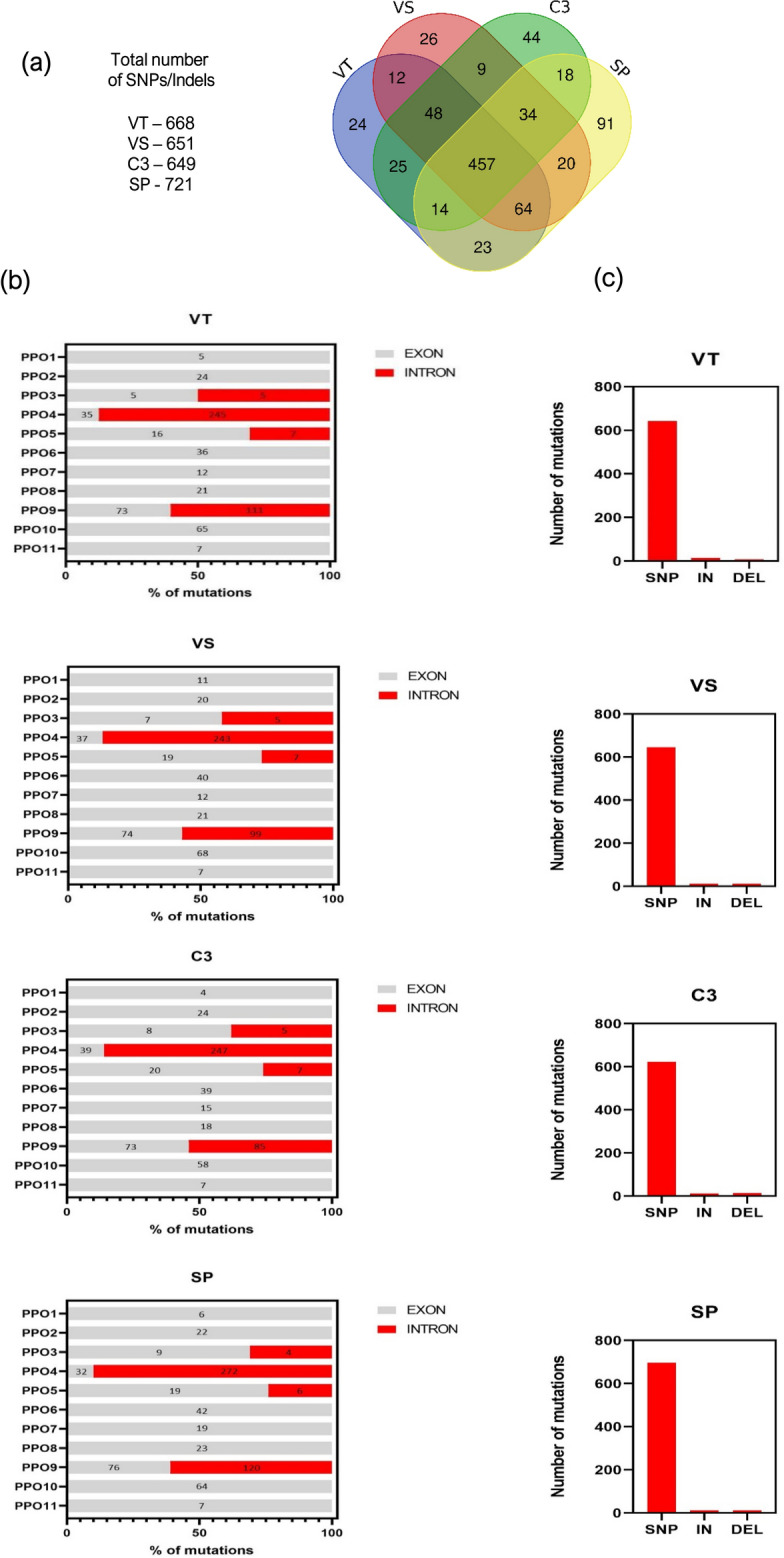
SNPs/indels analysis in *PPO* genes of four agronomically important globe artichoke varietal types. (**a**) The total number of polymorphisms and a Venn diagram indicating intersections among the four varietal types are reported. (**b**) The number of variants per *PPO*, divided in exon and intron regions, is reported. (**c**) The number of the different types of variants (substitutions, insertions and deletions) is indicated. Abbreviations indicate Violetto di Toscana (VT), Violetto di Sicilia (VS), Romanesco C3 (C3) and Spinoso di Palermo (SP).

The presence of allelic variants was evaluated in the *PPO* genomic sequences of four genotypes belonging to widely cultivated globe artichoke varietal types: ‘Violetto di Toscana’ (VT), ‘Violetto di Sicilia’ (VS), ‘Romanesco C3’ (C3) and ‘Spinoso di Palermo’ (SP). A list of all the SNPs/Indels identified with corresponding genomic features is reported in Supplementary Data S3. SNPs/Indels profile was similar among the four varietal types (Fig. 3). A range of 649–721 polymorphisms/varietal type was identified (Fig. 3a), with an average number of 672. A total of 457 polymorphisms were conserved across the four varietal types (Fig. 3a). The lower number of variants was detected in *PPO1* and *PPO11* (6 and 7, respectively) while *PPO4* and *PPO9* showed the highest major number of variants (287 and 155, respectively) (Fig. 3b). In *PPO4* and *PPO9* the majority of variants were found in intron regions, while in *PPO3* and *PPO5* in exons (Fig. 3b). The type of variants was also highly conserved among the four varietal types, since Indels were present in a range of 2 ± 0.5% and the vast majority was represented by SNPs (substitutions) (Fig. 3c).

Each identified variant was evaluated for its impact on the corresponding gene translation (Table 1). In VT five high impact variants were located in three PPO sequences: two in *PPO4* (1 bp substitutions), one in *PPO5* (4 bp insertion) and two in *PPO9* (20 bp deletion and 1 bp substitution) (Table 1). In VS and in SP the same two Indels were identified in the exon of *PPO5* (4 bp insertion) and *PPO9* (20 bp deletion) (Table 1). In C3, a 1 bp deletion was present in *PPO4* while a 4 bp insertion in *PPO5*. A high impact 4 bp insertion in homozygosis was found in *PPO5* of VT, which being located at the beginning of the gene sequence, presumably leads to a frame-shifting in gene translation with the introduction of early-termination mutations causing the production of a not functional protein.

**Table 1:** Summary of high impact variants in the *PPOs* of four globe artichoke varietal types.

Varietal type	Gene	Locus	Gene position	SNP/Indel position	Reference	Alternative	Genotypic status	Effect
VT	*PPO4*	V2_02g003640.1.01	3831196–3844009	3831685	G	C	Het	ETM
	*PPO4*	V2_02g003640.1.01	3831196–3844009	3842888	G	C	Het	ETM
	*PPO5*	V2_02g003650.1.01	3860408–3862336	3860433	AAC	AACGTAC	Hom	FS + ETM
	*PPO9*	V2_17g002890.1.01	3195388–3206783	3206527	TTGTCAATGACAAAGACAATGT	TT	Het	FS + ETM
	*PPO9*	V2_17g002890.1.01	3195388–3206783	3206699	C	A	Het	ETM
VS	*PPO5*	V2_02g003650.1.01	3860408–3862336	3860433	AAC	AACGTAC	Het	FS + ETM
	*PPO9*	V2_17g002890.1.01	3195388–3206783	3206527	TTGTCAATGACAAAGACAATGT	TT	Het	FS + ETM
C3	*PPO4*	V2_02g003640.1.01	3831196–3844009	3843437	ACC	AC	Het	FS + ETM
	*PPO5*	V2_02g003650.1.01	3860408–3862336	3860433	AAC	AACGTAC	Het	FS + ETM
SP	*PPO5*	V2_02g003650.1.01	3860408–3862336	3860433	AAC	AACGTAC	Het	FS + ETM
	*PPO9*	V2_17g002890.1.01	3195388–3206783	3206527	TTGTCAATGACAAAGACAATGT	TT	Het	FS + ETM

For each variant, the position into the genome, and the reference and alternative sequences are reported.

VT, Violetto di Toscana; VS, Violetto di Sicilia; C3, Romanesco C3; SP, Spinoso di Palermo; Het, Heterozygous; Hom, Homozygous; ETM, Early-termination mutation; FSM, Frame-shifting mutation.

### In silico prediction of a putative TFBS based-gene regulatory and functional profile of globe artichoke *PPOs*

The promoter sequence of the 11 *PPOs* (up to a 1 Kb regulatory sequence upstream of the ATG translation start site) was screened by the PlantPAN software, with the goal to identify putative transcription factor binding sites (TFBSs), and the corresponding transcription factors (TFs) possibly involved in the regulation of their expression. The TFBS database of *A. thaliana* was used as reference. The TFBSs as well as corresponding TFs and TF families are reported in Supplementary Data S4. The identified TFBSs were distributed fairly evenly along the *PPO* promoters and ranged from 46 (*PPO2*) to 150 (*PPO10*) with an average of 68 TFBSs/gene promoter (Table 2). The corresponding TFs able to recognize the identified TFBSs grouped into a multitude of TF families (Table 2), whose number was not proportional to the number of corresponding TFBSs. For instance, *PPO10* promoter, showing the highest number of TFBSs (150) was associated with just 26 TF families, while 29 TF families were associated to the 67 TFBSs identified in the *PPO1* promoter (Table 2).

**Table 2:** Number of transcription factor binding sites (TFBSs) and corresponding transcription factors (TFs) families identified in the promoter sequences of globe artichoke PPO genes.

Gene	Total number of TFBS	Total number of TF family
*PPO1*	67	29
*PPO2*	46	21
*PPO3*	58	24
*PPO4*	68	23
*PPO5*	62	21
*PPO6*	48	18
*PPO7*	92	23
*PPO8*	58	19
*PPO9*	52	22
*PPO10*	150	26
*PPO11*	51	23

**Figure 4:**
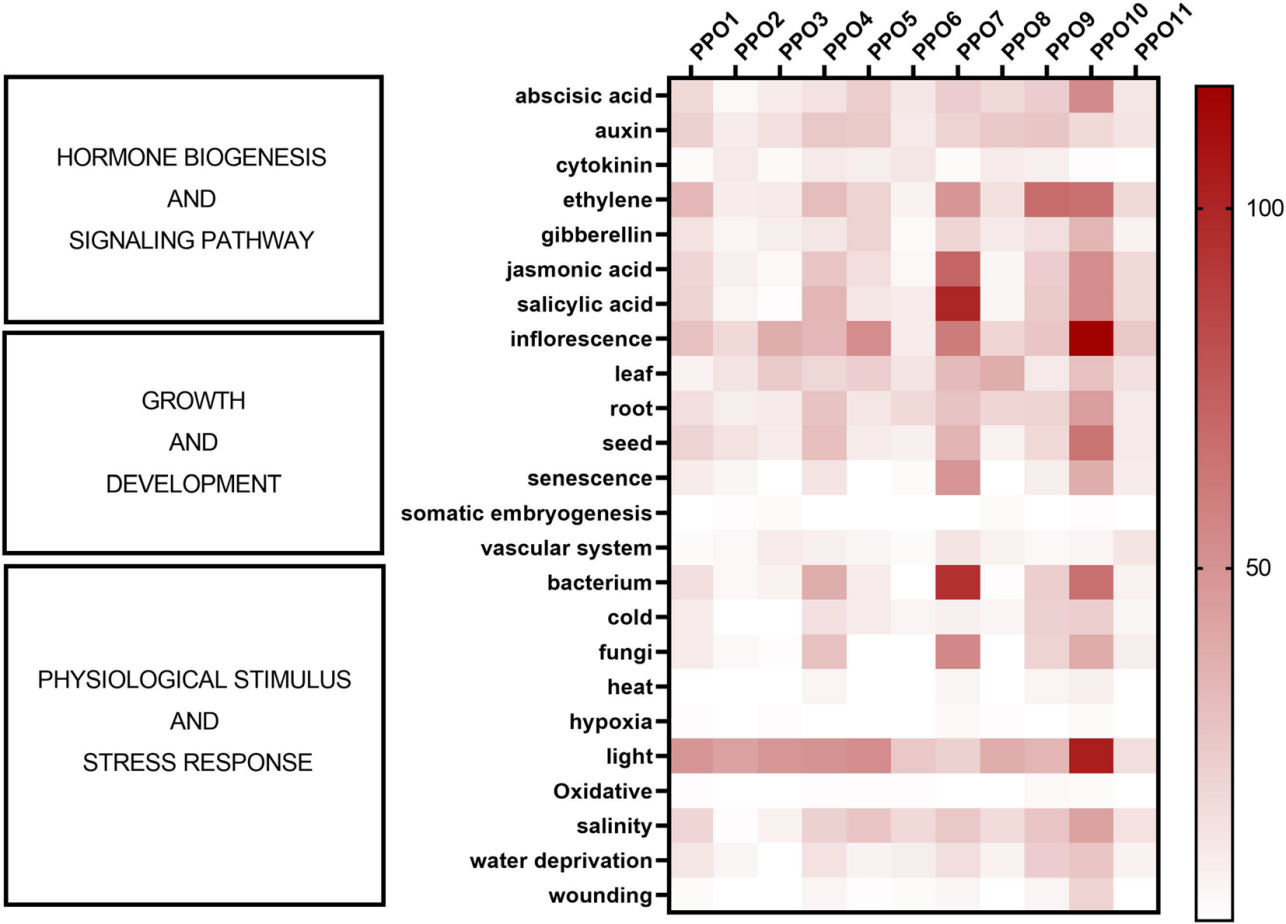
Putative transcription factor (TF) binding sites (TFBSs)-based regulatory and functionally profile of globe artichoke *PPOs*. Heat map showing the putative TFBSs-based functional profile of *PPO* genes. According to the identified TFBSs and related TFs, for each PPO the corresponding biological processes annotated (retrieved by PlantTFDB 5.0 database, planttfdb.cbi.pku.edu.cn) are reported on the left side of the heat map.

Based on the available gene ontology information retrieved by the PlantTFDB database, related to the biological processes associated with each identified TF, a putative regulatory and functional profile for each *PPO* promoter was generated (Fig. 4). In detail, *PPO* promoters were found to be putatively regulated during several biological processes included into three macro-groups: (i) hormone biogenesis and signaling pathways; (ii) growth and development; (iii) physiological stimulus and stress response. With regard to the first group, the activity of *PPO* promoters (mainly *PPO4*, *PPO7*, *PPO9* and *PPO10*) was associated with ethylene, salicylic acid and jasmonic acid pathways that are well known to be involved in the response to different abiotic and biotic stressors. On the other hand, the activity of *PPO* promoters included in the second group (*PPO5*, *PPO7* and *PPO10* with high impact) appeared to be potentially regulated during the inflorescence growth and development. The *PPO7* and *PPO10* promoters showed a potential activity during senescence as well, which is associated to tissue browning. Finally, with regard to the third group, a potential regulation of *PPO4*, *PPO7* and *PPO10* promoters resulted associated with plant response to biotic stresses, such as bacteria and fungi. Notably, all *PPO* promoters showed a high impact regulation upon light stimuli.

### Tissue- and organ-specific transcriptional levels of *PPOs* in globe artichoke

In addition to in silico analyses, the assessment of changes in the expression profile of *PPO* genes in different plant tissues and in response to environmental stimuli, such as wounding, may shed light into the biological roles of this important globe artichoke gene family. To this end, the expression pattern of *PPO* genes was investigated by RTqPCR analyses in leaf, stem and capitulum tissues of the VT globe artichoke varietal type, chosen for its high tendency to browning due to cutting (Supplementary Fig. S1). In capitula the analyses were carried out in external and internal bracts as well as receptacle before and after cutting (i.e. basal expression, T0; expression 15 min after cutting, T15).

**Figure 5:**
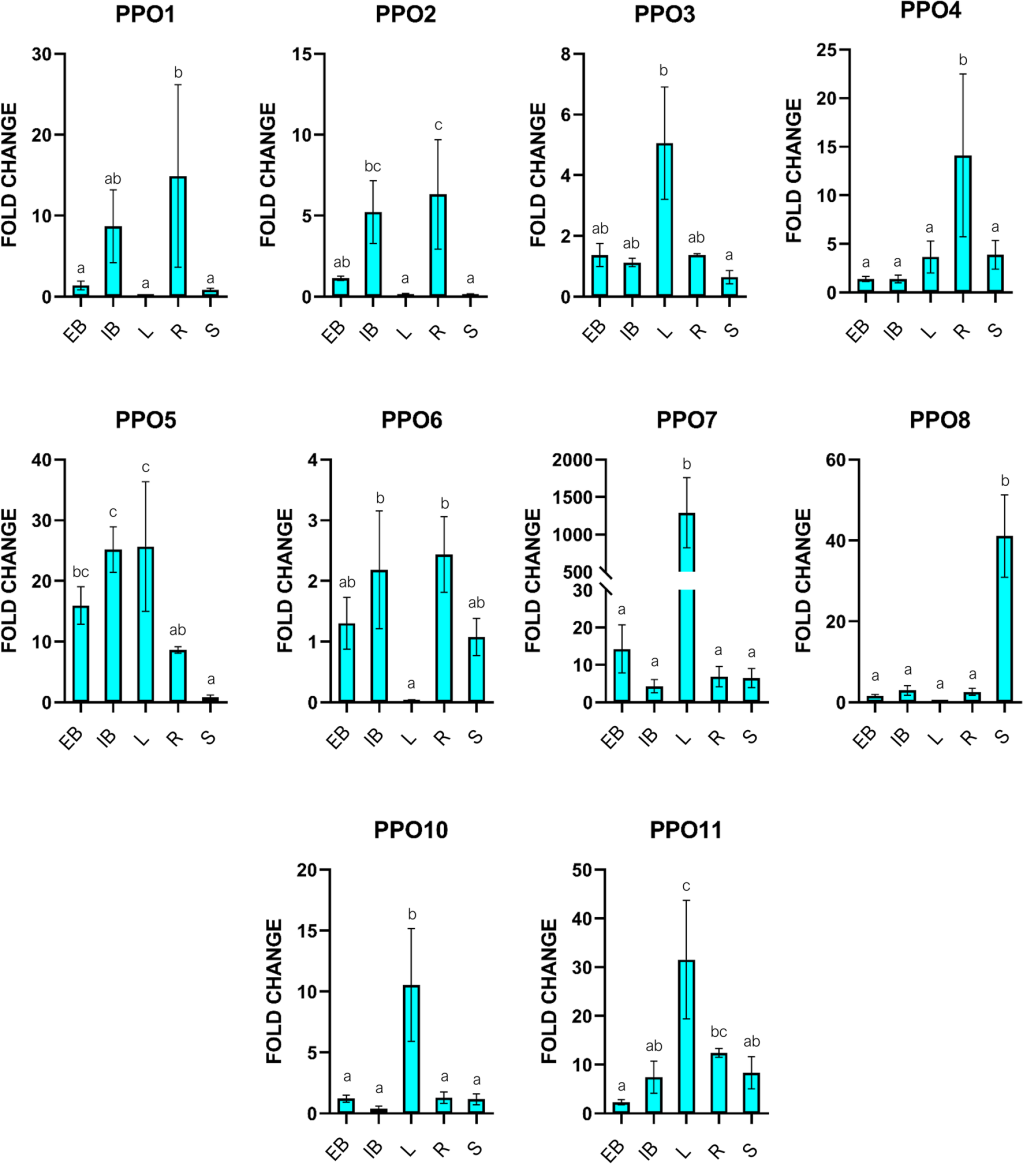
Transcriptional level of VT *PPO* genes in different plant tissues of Violetto di Toscana (VT) globe artichoke varietal type. Each graph shows the basal transcriptional level of one *PPO* in the tested tissues. Bars indicate mean values ± SE of the technical duplicates and three biological replicates. Letters indicate statistically significant differences of the datasets according to a one-way ANOVA test followed by a Tukey’s HSD (*p* value ≤ 0.05). External bract (EB); Internal Bract (IB); Leaf (L); Receptacle (R); Stem (S).

Following the assessment of *PPOs* basal expression, a heterogeneous expression profile was observed (Fig. 5). *PPO3*, *PPO7*, *PPO8*, *PPO10* and *PPO11* were expressed in all the tissues, however, the transcription of *PPO3*, *PPO7*, *PPO10* and *PPO11* was significantly higher in leaves (L) while the one of *PPO8* in stems (S). *PPO4* was also detected in all tissues but its expression was significantly induced in capitulum’s receptacle (R). *PPO1* and *PPO2* showed a similar expression profile being mainly and significantly expressed in capitulum’s internal bracts (IB) and receptacle. *PPO5* was significantly expressed in leaves and capitulum’s tissues, such as external (EB) and internal bracts. *PPO6* was not detected in leaves but its expression was significantly high in capitulum’s internal bracts and receptacle. Interestingly, *PPO9* was not detected in any of the analyzed tissues.

**Figure 6:**
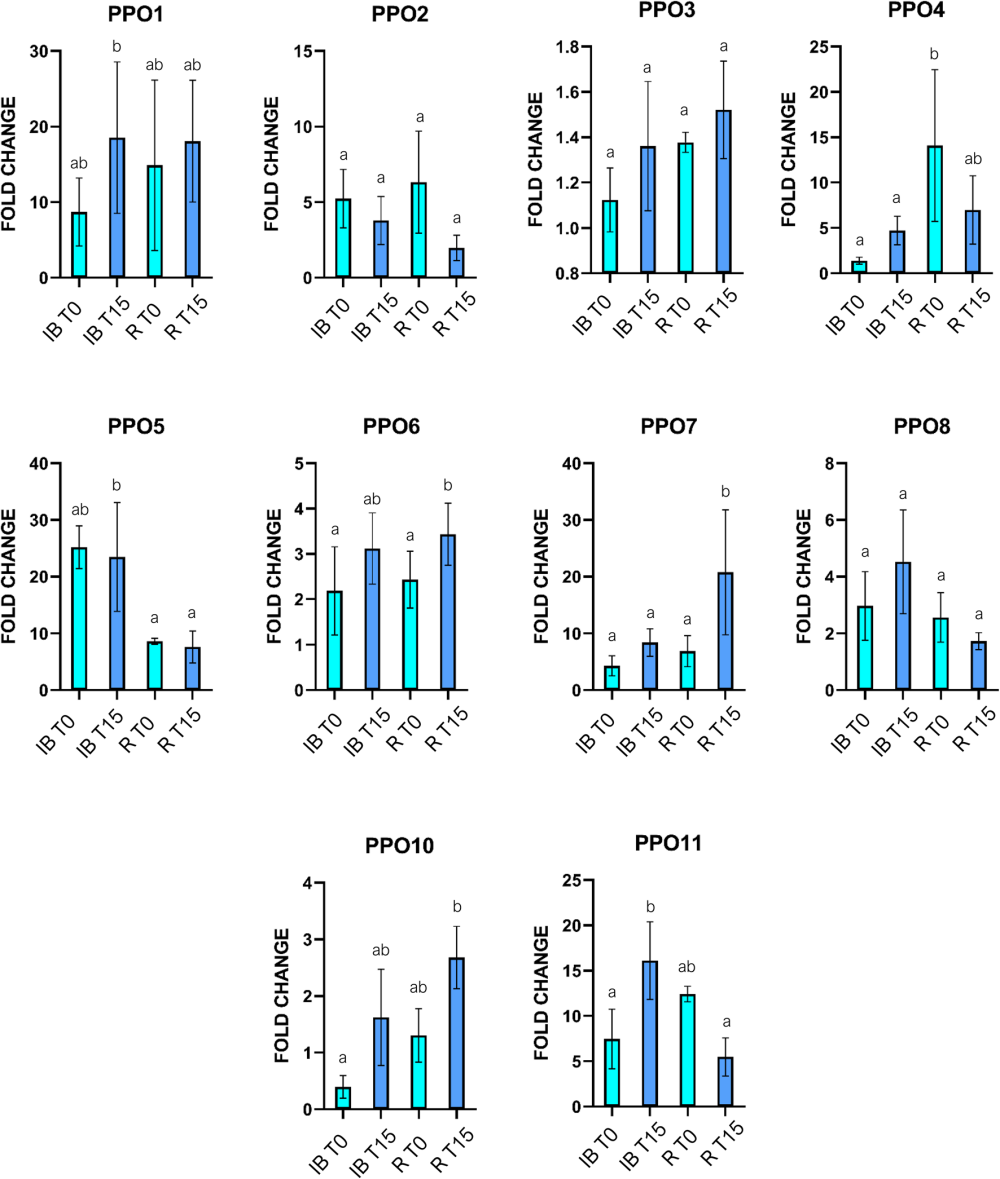
Expression level of VT *PPO* genes of Violetto di Toscana (VT) globe artichoke varietal type upon wounding. Each graph shows the expression level of one *PPO* in the tested tissues at T0 and T15 after cutting. Bars indicate mean values ± SE of the technical duplicates and three biological replicates. Letters indicate statistically significant differences of the datasets according to a one-way ANOVA test followed by a Tukey’s HSD (*p* value ≤ 0.05). Internal Bract (IB); Receptacle (R); Time zero (T0); Time 15 min (T15).

In the capitulum, 15 min after cutting, *PPO1* and *PPO10* showed positive fluctuations in expression levels in both receptacle and internal bracts (Fig. 6) while *PPO6*, *PPO7* were significantly upregulated in the receptacle and *PPO11* in internal bracts. On the contrary, *PPO3*, *PPO4*, *PPO5*, and *PPO8* did not highlight any significant increase in their expression activity while *PPO9* expression was not detected.

**Figure 7:**
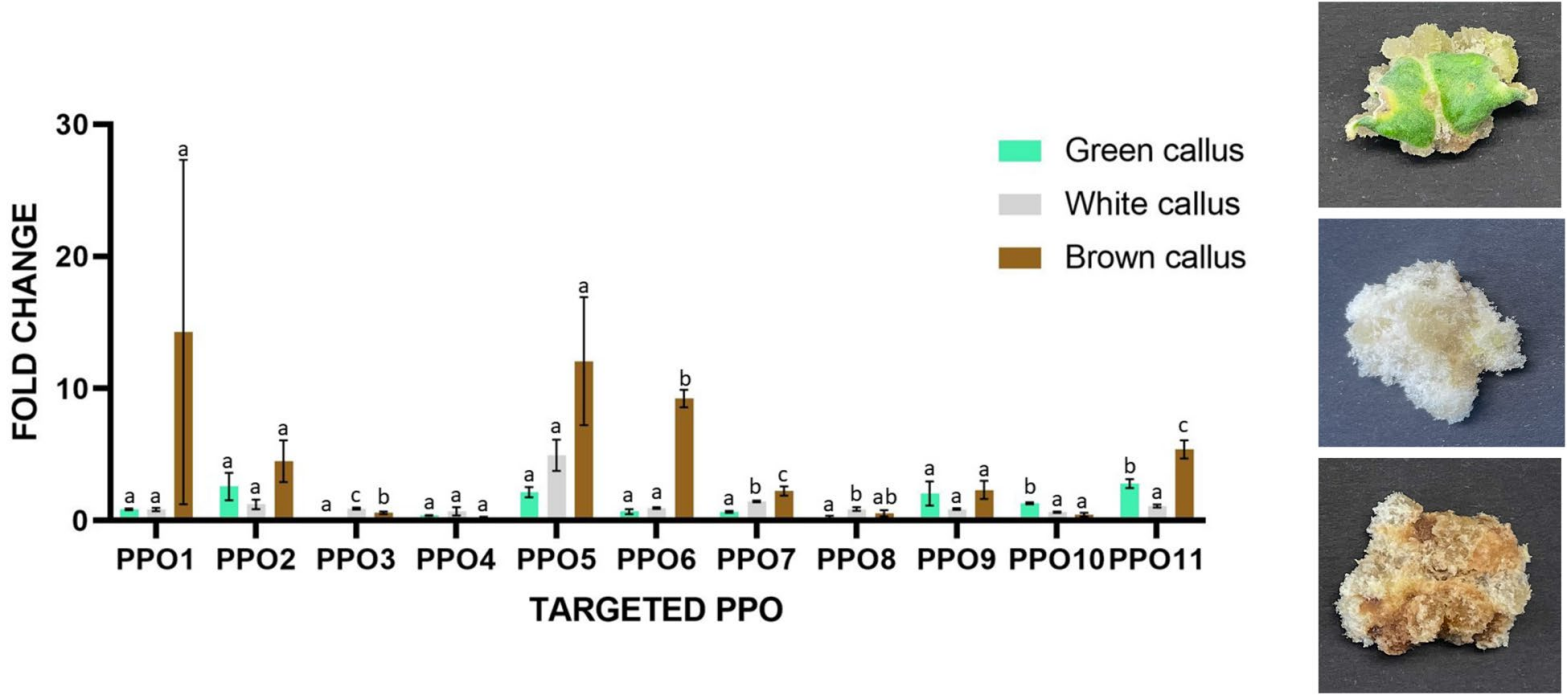
Expression levels of *PPOs* in different *callus* morphologies of the Spinoso Sardo varietal type. Three different callus phenotypes (pictures on the right) were tested in RTqPCR analysis for all the *PPOs.* In the graph, Bars indicate mean values ± SE of the technical duplicates and three biological replicates. Letters indicate statistically significant differences of the datasets according to a one-way ANOVA test followed by a Tukey’s HSD (*p* value ≤ 0.05).

In order to acquire more information regarding the involvement of *PPOs* in the browning of calli during in vitro culture, the expression levels of *PPO*s were also evaluated in three calli types of the ‘Spinoso sardo’ varietal type, showing different morphologies. This varietal type was chosen due to the availability of efficient in vitro maintenance protocols^28^ (Fig. 7). Three types of calli were analyzed: white, green and brown. RTqPCR analyses highlighted that *PPO6*, *PPO7* and *PPO11* were significantly upregulated in brown calli in respect to green and white calli. *PPO1* and *PPO5* also appeared weakly upregulated in brown calli, but their increased expression was not statistically significant. Differently, *PPO3* and *PPO8* showed a significant upregulation in white calli while *PPO10* was significantly upregulated in green calli. Finally, *PPO2*, *PPO4* and *PPO9* showed no significant differences in transcripts levels in all types of tested calli.

## Discussion

Globe artichoke’s antioxidant capacity is one of the highest among vegetables, due to its high content in polyphenols (chlorogenic acid, cynarin, luteolin 7-O-rutinoside, and luteolin 7-O-glucoside)^29^. However, the activity of the polyphenol oxidase enzymes, by catalyzing the oxidation of phenols, cause an undesirable browning in globe artichoke heads cut during the processing or storage before fresh consumption. This enzymatic reaction other than reducing the commercial value and shelf life of capitula, also affects the antioxidant capacity of the product, resulting in its decreased nutritional value. Physical and chemical treatments are applied to prevent the browning process, but they are not completely efficient and may induce negative effects on the product quality^18,30^.

In last few years, different research works have set up genetic engineering protocols aiming at the inactivation of PPO’s in species of agronomic interest, such as apple, potato, eggplant, mushroom, resulting in reduction of enzymatic browning^11,21–24^. Among them, the CRISPR/Cas9 technique has shown to provide the most user-friendly tool for targeted gene loss-of-functions and has added advantage of gene knockout over RNAi, as it targets the endogenous genes with more precision and simplicity.

In 2013, the first gene coding for a globe artichoke *PPO* was isolated and its sequence corresponds to our *PPO7*^31^. The 1764 bp *CcPPO* sequence was found to encode a putative protein of 587 amino acids and the analysis of the promoter region revealed the presence of cis-acting elements putatively involved in the response to light and wounds. The *PPO* resulted upregulated 48 h after wounding, even though the browning process had started earlier, presumably because other PPOs were also implicated in the phenomenon. Indeed, PPOs are encoded by a gene family whose member numbers varies significantly among species^32^, and the number of *PPO* genes in a plant is not directly related to the size of its genome^33^.

Thanks to the availability of the whole globe artichoke genome sequence^25,26^, we identified and isolated 11 *PPO* genes. Bioinformatic analyses made it possible to acquire information on *PPOs* genomic and transcriptional features, and their structural genetic and putative protein profiles were generated. One-third of the isolated globe artichoke *PPOs* contained introns in the sequence (*PPO3*-*5* and *PPO9*), similarly to what was reported for *Populus trichocarpa*^34^. Intron–exon *PPO* genes were also detected in pineapple^35^ and wheat^36^, while *PPOs* in *Solanaceae* species (potato, tomato and eggplant) lack introns^11^. Previous studies have shown that introns play essential roles in the regulation of the transcriptome^37^, and that the absence of introns contributes to a more rapid transcription of genes involved in stress or defense responses^38^. This may suggest different functions among members of the globe artichoke *PPO* gene family.

The analysis of putative TFBSs called attention to some *PPOs* whose promoter is regulated by light stimuli, wounding, and hormones signaling (ethylene, jasmonic acid and salicylic acid) and biosynthetic pathways involved in senescence and defense against biotic attacks, processes which are involved in tissue browning^39,40^.

The phylogenetic analyses highlighted a high sequence similarity among five CcPPOs (PPO1-PPO5) located in chromosome 2, presumably paralogues and the result of ancestral gene duplication. Interestingly, the presence of these five genes on the same chromosome is highly similar to that of *L. sativa* PPO orthologs. In lettuce these PPOs were functionally characterized and found to be activated by different physical stimuli, such as pH, heat and light^41^. Differently, PPO8 grouped mainly with PPOs from *T. officinale* that has been the object of studies for its high content of PPOs in latex which acts as wound sealing^42^, while PPO7/11 and PPO6/10 appeared more distantly related.

The allelic variants of the eleven globe artichoke *PPOs* were analysed in four genotypes belonging to as many varietal types using the v2.0 globe artichoke genome^26^ as reference. A range of 600–700 variants per variety was identified, the majority of which was represented by SNP. Interestingly, in the VT varietal type an insertion of 4 bp in the 5′-end of *PPO5* coding sequence was found in homozygosis. This mutation affects the correct gene translation originating an unfunctional protein. The occurrence of natural variants affecting the translation of members of the *PPO* gene family have been recently reported in *Triticum aestivum*, in which the genomic diversity of the *23* wheat *PPO* genes was investigated across a population of 207 wheat varieties and variants associated to a decreased level of PPO activity^43^.

To identify *PPOs* putatively associated with globe artichoke tissue browning in different organs and tissue, the transcriptional levels of the eleven isolated *PPOs* were evaluated by RTqPCR in plant tissues and capitula (pre- and post-cutting) of the VT varietal type as well as in calli of the SP varietal type characterized by different phenotypes. VT was chosen due to its high content of phenols^44^ and a marked tissue browning after cutting while SP for the availability of efficient in vitro maintenance protocols^28^. *PPOs* activity has been reported to vary from one organ to another and inside an organ, depending on the tissue considered^45^. Indeed, the transcriptional profiles revealed heterogeneous levels of *PPOs* expression in both plant tissues and in capitula upon wounding. In the latter, transcripts level 15 min after cutting highlighted that *PPO6*, *PPO7* and *PPO11* were significantly up-regulated and thus putatively involved in tissue browning, while *PPO1* and *PPO10* played a minor effect. Interestingly, consistent results were obtained by analyzing *PPOs* expression level in calli tissues, as a significant increase in the expression of *PPO6*, *PPO7* and *PPO11* was observed in brown in respect to white and green calli.

A bottle-neck in the application of CRISPR/Cas9 gene knock-out in globe artichoke in represented by its recalcitrancy to in vitro plant regeneration from calli after genetic manipulation. According to the literature, in 1990s, globe artichoke calli were transformed with reporter genes for the first time, but no plant regeneration was obtained^46^. More recently we developed an in vitro culture protocol, which resulted in a high frequency of callus induction making use of absorbers of polyphenols and inhibitors of polyphenol oxidase, highlighting that tissue browning caused by phenolic compounds oxidation represents a key factor hampering callus induction and subsequent plant differentiation^5,47^.

Our future research will be focused on setting up a *PPO*-based gene editing approach for simultaneously knock-out the three key candidate *PPO* genes: i.e. *PPO6*, *PPO7* and *PPO11* which putatively play a key role in inducing tissue browning in globe artichoke capitula as well as the onset of oxidation of the phenols in in vitro calli hampering plant regeneration. This will allow to generate genotypes of globe artichoke characterized by better shelf life of the capitula and to overcome the use of physical and chemical treatments needed to reduce their browning during industrial transformation.

## Methods

All methods were performed in accordance with the relevant guidelines/regulations/legislation.

### Identification and structural characterization of PPOs

To identify all PPOs in *C. cardunculus* var. *scolymus*, PPOs protein sequences previously annotated in the v.1.0 proteome^25^, and available in the Globe Artichoke Genome Database (https://www.artichokegenome.unito.it), were used for a BLASTp analysis (https://blastp.ncbi.nlm.nih.gov) against the v2.0 globe artichoke reference proteome^26^ using an e-value threshold of 1e^-5^, and a HMMER analysis (https://hmmer.org) using default parameters. The identified putative PPO protein sequences (v2.0) were downloaded and their features annotated in Supplementary Data S1. Based on their annotation, the corresponding gene and promoter sequences (1 Kbp upstream the translation start site) were isolated as well and retrieved from the v2.0 globe artichoke reference genome^26^ available at the Artichoke Genome Database (http://www.artichokegenome.unito.it).

The identified putative PPO protein sequences were analyzed using the Pfam software (https://pfam.xfam.org/) to predict protein structure domains (Fig. 1). Instead, *PPO* genomic sequences were analyzed with the Wormweb software (https://wormweb.org/exonintron) to graphically generate a *PPO*-specific exons-introns profile (Fig. 1).

### Phylogenetic analysis of PPOs

The identified putative PPO protein sequences (v2.0) were used for a BLASTp search to find homologous in the non-redundant protein sequences NCBI database of 8 *Asteraceae* species (*T. officinale, L. sativa, H. annuus, G. jamesonii, Dahlia pinnata, M. micrantha, L. saligna, E. canadensis*), using an e-value cut-off of 1e^-5^. All the identified PPO protein sequences (Supplementary Data S2) were aligned with the globe artichoke PPO sequences with the Clustal omega multiple alignment program (https://www.ebi.ac.uk/Tools/msa/clustalo/), using default parameters. The aligned PPOs were then used to construct a phylogenetic tree applying a Neighbor joining algorithm^48^, and confidence level was established for each node by performing a bootstrap analysis with 1000 iterations. The IQTREE (http://www.iqtree.org) tool (Fig. 2) was used to infer the best phylogenetic tree by maximum likelihood.

### SNP/Indel discovery in PPO sequences of globe artichoke genotypes

The SNP/Indel analysis was conducted by mapping the *PPO* sequences (fastq) of four widely cultivated globe artichoke varietal types: ‘Violetto di Sicilia’ (VS), ‘Violetto di Toscana’ (VT),’ Spinoso di Palermo’ (SP) and ‘Romanesco C3’ (C3), to the v2.0 reference genome, using a Burrows-Wheeler Aligner program (BWA) with default parameters. The SNP/Indel calling was performed on the BAM files using Samtools mpileup with default parameters except for: (i) filter multimapping events (− q > 1) and (ii) minimum mapping quality (Q = 20). A variant call format (vcf) file was produced. SnpEff software was then used to annotate the identified allelic variants and evaluate their impact on the protein function.

### In silico search of transcription factor binding sites in the promoter sequences of *PPOs*

To detect putative transcription factor binding sites (TFBSs) and corresponding transcription factors (TFs) putatively involved in the regulation of *PPOs* expression, *PPO* promoter sequences (1 Kbp upstream of the ATG translation start codon) were examined by the “promoter analysis tool” of PlantPAN 2.0 (https://plantpan2.itps.ncku.edu.tw/promoter.php). TFBSs calling was performed against the *A. thaliana* reference database by considering a similarity score set to 0.95 and only the coding strand comprising the *PPO* sequence. For each TFBSs, the corresponding TFs identified were investigated by the PlantTFDB 5.0 database (http://planttfdb.gao-lab.org/) in order to acquire information on their biological functions.

### Plant material sampling in vivo and in vitro

The ‘Violetto di Toscana’ varietal type was used to analyze the expression of *PPO* genes in the plant tissues sampled from leaves, stems and capitula; in the latter the tissues were collected also 15 min after cutting. Plant material of the ‘Violetto di Toscana’ varietal type were collected in three biological replicates from as many clonally propagated 1-year-old plants, grown under usual agricultural practices at the experimental fields of DISAFA (University of Turin, Carmagnola, TO, Italy). Each sampled capitulum was vertically cut in four parts (Supplementary Fig. S1), of which two used for sampling tissue portions of about 2 × 1 cm of surface and 0.5 cm of thickness of the two external bracts, internal bracts and receptacle. The sampled tissues from one part of capitulum were immediately frozen in liquid nitrogen (T0) while sampled tissues on a second part of the same capitulum were sampled after 15 min and then frozen in liquid nitrogen. Side by side leaf samples of fully developed leaves as well stem portion were sampled and immediately frozen in liquid nitrogen. All the samples were stored at − 80 °C before being used for RNA extraction.

The ‘Spinoso sardo’ varietal type, was used to evaluate gene expression of *PPO* genes in calli characterised by different phenotypes. Plantlets, propagated in vitro and kindly provided by the Agenzia per la Ricerca in Agricoltura (AGRIS SARDEGNA, Cagliari, Italy) were maintained on an in-house propagation medium (4.4 g L ^−1^ of MS with vitamins, 30 g L^−1^ of sucrose, 0.5 mg L^−1^ of benzyl-aminopurine (BAP) and 7 g L^−1^ of plant agar) and transferred on a new medium every 4 weeks. In vitro culturing was conducted in a growth chamber at 24 ± 1 °C and a photoperiod of 16 h light/8 h darkness. From two weeks old plantlets, leaf explants of 5–10 mm were picked up and transferred on a callogenesis induction medium containing 4.4 g L^−1^ of MS with vitamins, 30 g L^−1^ of sucrose, 1 mg L^−1^ of BAP, 3 mg L^−1^ of 1-naphtaleneacetic acid (NAA), 5 mg L^−1^ ascorbic acid, 5 mg L^−1^ citric acid and 7 mg L^−1^ of plant agar^5^. Culturing was performed for 6 weeks at 24 ± 1 °C in two regimes in order to obtain different *callus* types: (i) full darkness for 6 weeks to obtained white calli; (ii) full darkness for 4 weeks followed by 2 weeks with a 16/8 h light/dark period to obtain green calli, most of which turned to brown calli due to phenols oxidation. After 6 weeks, about one cubic centimeter of tissue was collected from calli of each morphological type, immediately frozen in liquid nitrogen and then stored at -80 °C. For each type of callus, three samples were collected from three different calli and used for the subsequent RNA extraction procedure.

### RNA extraction, synthesis of cDNA, RTqPCR and statistical analysis

RNA was extracted from plant tissue samples using the “Spectrum plant total RNA kit” (Sigma-Aldrich, St. Louis, USA), according to manufacturer’s instructions. Extracted RNA was treated with DNase I (Thermo Fisher Scientific) to remove contaminant genomic DNA and quantified on the NanoDrop 8000 Spectrophotometer (Thermo Fisher Scientific).

For each sample, synthesis of cDNA was performed by reverse transcription using 1 µg of extracted RNA and the “High-Capacity cDNA Reverse Transcription Kit” (Applied Biosystems, USA), following protocol’s instructions. The produced cDNA was used for RTqPCR analysis.

RTqPCR reactions were conducted on a “StepOnePlus Real-Time PCR system” (Applied Biosystem), in 96-well plates, in technical duplicates and three biological replicates, and carried out in a 10 µL final volume containing 10 ng of starting cDNA, the “2X Power SYBR Green PCR Master Mix” (Applied Biosystem, USA) and the couples of primers (0.3 µM) for *PPOs* (Supplementary Table S1) or *Actin*^49^ amplification. The following PCR protocol was used: 1 cycle of 95 °C for 10 min; 40 cycles of 95 °C for 15 s, 60 °C for 60 s. At the end of the amplification, the melting curve analysis was performed to assess primer pair specificity. The obtained RTqPCR data were quantified using the 2^−ΔCt^ method based on Ct values of *PPO* genes and *Actin* (*ACT*) used as housekeeping gene. For statistical analyses, the IBM SPSS statistical software was used to perform a one-way ANOVA test followed by a Tukey’s HSD test (*p* value ≤ 0.05) to assess differences between each value (the latter corresponding to the mean of technical duplicates and three biological replicates).

## Supplementary Information

Supplementary Information 1.

Supplementary Information 2.

Supplementary Information 3.

Supplementary Information 4.

Supplementary Information 5.

Supplementary Information 6.

## Data Availability

Further inquiries on our data can be directed to the corresponding authors (VP, SL).
