# Supplementary material for: Structural and expression analysis of polyphenol oxidases potentially involved in globe artichoke (C. cardunculus var. scolymus L.) tissue browning
Source: Sci Rep. 2023 Jul 29;13:12288. doi: 10.1038/s41598-023-38874-4 (PMC10387078; doi:10.1038/s41598-023-38874-4)
Supplement: Supplementary file 2 — Supplementary Information 2. [file 41598_2023_38874_MOESM2_ESM.pdf]

TITLE

Structural and expression analysis of polyphenol oxidases potentially involved in globe artichoke (C. cardunculus var. scolymus L.) tissue browning

AUTHORS

Valerio Pompili, Elena Mazzocchi, Andrea Moglia, Alberto Acquadro, Cinzia Comino, Giuseppe Leonardo Rotino, Sergio Lanteri

Supplementary Data S2. Sequences of PPOs used in this study for the construction of the phylogenetic tree. The names of the species are reported in the first part of each sequence header as follows: *Taraxacum officinale* (to), *Lactuca sativa* (ls), *Heliantus annuus* (ha), *Gerbera jamesonii* (gj), *Cynara cardunculus* var. *scolymus* (cc), *Dahlia pinnata* (dp), *Mikania micrantha* (mm), *Lactuca saligna* (lsal), *Erigeron canadensis* (ec). These supplementary data are related to Figure 2.

>to-Tl_CBZ41491.1 polyphenol oxidase precursor [Taraxacum officinale]

MASLTFSTLPTSTTIKKPLFSKTSSHLNQSHRFKVSCNAAANNDDKTVKNSETPKLILPFE
MQNVDRRNVLLGLGVGGLYGAANLTTNIPSAFGIPITAPDNISDCVTASSNLRNSADAV
RGLACCPPVLSTDKPKDYVLPTNPVTRIRPAAQRATSDYVAKYQQAIQAMKDLPDDHP
HSWKQQGKIHCAYCNGGYSQGQTGHPELQLQIHNSWLFFPFHRWYLYFYEKILGKLIN
DPSFAIPFWNWDNPTGMVLPGMFEDNGRNNPLFDPFRDATHLPPTIFDIEYAGKDTGAS
CIDQIGINLPSMYRQMITNASDTTSFFGGEFVAGDDPLNKQFNVAGSIEAGVHTAAHRW
VGNPRMANGEDMGNFYSAGYDPLFYVHHANVDRMWKIWKDVDRKGHKDPTAGDWL
NASYVFYDENENLVRVYNRDCVDVRKMGYDYERSEIPWIRSRPTAHAKGANVAAKSV
GIVQKVEDVIFPLKLNKIVKVLVKRPATNRTKEDKEKENELLFVNGIQFDGERFVKFNVF
VNDVDDGVETTAASSEFAGSFAQLPHNHGDKMLMRSGVAFGITELLEDIEAEGDESILV
TLVPKTGCDELTISEITIKLVPVV

>to-CCA94610.1 polyphenol oxidase precursor [Taraxacum officinale]

MASLQSLFIPTITTRTTLPNPTSNRRSKTYPKVSCNATSDDNEKLLILPETQKLILPTTLNV
DRRNLLLGLGAGLYSTVNFTSAPAAFADPITTPDISTCVASTKGFQPKNAVRTNACCPPT
LSKTVKEFVFPNDRATRIRPAAHRASPEYIAKYKAAIQKMRDLEDDDPHSFVSQAKIHC
AYCNGGFTQDKHPDKELQIHNSWLFFPFHRWYLYFYERILGKLIDDPTFAIPYWNWDNP
AGMSLPDFFEEGGKANPAFDAYRNSSHLAPAIADLDYDGTEKNYTCIKQLSVNLGAMN
KQMIRNAGDWKSFFGGEYRAGSDPIGNKDPSVGSVEAGCHTAIHRWVGNSRLPNGED
MGNFYSAGYDPVFYVHHANVDRMWKVWKDLGIRGHIEPPDNDWRNASYVFYDENKE
LVRVYNKDCVRLEKLKYDYQFAPIPWKNSRPVARTKKSNIALKSVGTVKQAQDSKFPL
KLDKTTKVLVKRPATNRSKEDKENANEVLLIKGVVFNGEKFVKFDVFVNDKDDVTAST

AEESEFAGSFAQLPHNHGDKMLMTSSARFGLTELLEDIDAEDDESILVTLVPKVGCEEVT
IGEIKVELVPIEE

>to-CCD61123.1 polyphenol oxidase precursor [Taraxacum officinale]

MASLQLLTTFTTTATPRKPPNPTSSRRSKTYPKQTHRFKVSCNVAKDDNENLLLVPESQ
KLILNVDRRNLLLGLGGAGLYTSLPAAFAAPITTPDISTCIPSESGFNTEDSVRSNQCCPPA
TSSTIPKEFVFPKDTTTRIRPAAHRATPEYVAKYKAAIQAMRDLPDDHPHSFVSQAKIHC
AYCNGGYTQVASGFPDKMLQVHNSWLFFPFHRWYLYFYERILGKLIDDPTFALPYWN
WDHPAGMTLPAFFETDGKRNPVFDAYRNVTHVTPAAIVDLDYNGSDSGAPCLQQINTN
LTAMYKQMVSNAADPLSFFGGVYKAGDDPFGNGDPSVGSVEAGCHTAVHRWVGNPR
MMNTEDMGNFYSAGYDPAFYVHHANVDRMWKVWKDLGIPGHSEPTDPDWLNASFVF
YDENEQLVRVYNKDCVNLENLKYDYELSPLPWLKNRPVPHTPKPVDNAAKPAIKLPEV
KFPVKLDKTVKVYVKRPVKKRGKAEKDKVSEQLVLKGIKFNSTKFVKVDVFVNDQDD
APAILPSESEFVGSFAQLPHGRSGRGRGRGHGHSELMTSAARFGLTELLEDIGAEDDEYI
LVTLVPKAGSEDVTVEEITVELVPVNP

>to-CCD61124.1 polyphenol oxidase precursor [Taraxacum officinale]

MASLSSTLATVTLSCSPAFCKSAPTRRWRTQAKQTHRFQVPCLVTSDDHEKPPTTKPQP
QNLTPPQTSLARRNLLLGLGGLYSTTVNLTTQSPVFANPIKAPSLVPGCKNSDWNLDFES
GIRTGACCPPFAKELEIDYKFPTTGETRIRQPIHRASPEYIEKFKVAMQKMKALPDDDPCS
FKNQAKVHCAYCNGNYTQMASGYPEKILQVHYSWLFFPFHRWFLYFFERILGDLIEDKT
FGLPYWNWDNPAGMEIPAVFEEGGKSNPLYNSYRNVNHLRPAIVDLDYQMEERNMSPL
DQIRINMCNMNRRMMRNASDAKSFFGGEYVAGNNPTPREDSSIGSIEAGCHTAVHRWV
GDPRTPNHEDLGNFYSAAYDPLFYIHHSNVDRMWTLWKGFGGEGRIEPNDKNWEEAS
YVFYDENRKPVRVYNKHCVNLEDLKYEYEDSETPWKSNPPKPHPRSSVTTASPENMAA
MKFPLSISETVTVQVKRPETNRPKDQKKTIREILLLKGINFDGGKFVKFDVFVNVCDDISR
ITPCESQYAGAFGLLPHKRGEKPDSKTGVRIELTELLEEINADGDDSVRVTIVPRVGCDD
VTFEDIKIELIDISEKESK

>to-CCD61125.1 polyphenol oxidase precursor [Taraxacum officinale]

MASLTFSSLPTCTTTTTNKPFLFSKTSFTHSNQFKVSCTAADNNENPDILPKSSSFERRNL
LLGLGGLYGAANIPSAFGEPITAPNSATSCAEASGLKNPREAIRGLSCCPPLPLDGNDVPK
PYTFPTVAFDKLRVRKPAHLLVEDTAYIKQFNTAIQKMKDLPDNDPHSWKNQAKIHCA
YCNGGYTQVETGHPELTIQVHNSWLFLPFHRWYLYFLEKIMGKVLEDESFALPYWNWD
NPAGMMLPAMYEGNIKDPKNAIYDALREPSHLPPAIFDFNYANKETSPVLPDDEQKNVN
LALMYRQMISSGSSTLTFMGGQARAGLDSKDERVRSLSLGGSLENGVHTGAHRWVGNS
RMPNGEDMGNFYSAGFDPLFYGHHANVDRMWNVWKAMGKQLRLDGHIDPVDEDWL
NASYVFYDENRVRVRVYNKDSVHTPTMGYKYESSETPWVTRKPLARPREENVAVKAA

NDGVKNVSDEGVLPLTLEDGKLVRVLVSRPIEAKNRSPEDKKKTNEMLSLNNIYYSCQK
FIRFNVIVNDTGKKPAKDITAAQAEFVGSFAQLPHNAGEEDKMLMKTDELFGITELMED
TKAEGDDFILVTIVPKVGCTGVKITGIDIQMVDAEPAGYKF

>to-CCD61126.1 polyphenol oxidase precursor [Taraxacum officinale]

MASLSFSTLPTSTTTNKRLFTQSSPTHSNLFKVSCKAADDNENSDIPPKYSSFERRNLLLG
LGGLYGAANIPSAFGVPIEAPDSAASCFEAFSSVRNPKEALRGLACCPPIYSKAAPVPXVF
PTVDKKKLRERKPAHLLSQDADYVEQFRKGVQILKELPDDDPHSWKNQAKIHCAYCNG
GYRQEKSGFPDLEIQVHNSWLFLPFHRWYLYFLEKILGKHLEDDTFALPYWNWDNPVG
MSIPPIYEVKDPITIPDENGKQKIRSLYDPFRDQLHASPNAILDFNYGGQDVEITPKEQVNI
NLALMYRQMVSSGYNTRLFMGGEVKAGVDPISQNVKDLSLGGSLENGVHTAAHRWV
GNRRMPNSEDMGNFYSAGFDPVFYAHHANVDRMWNVWKALGKKLGKPGHVDPIKPD
WTNASYVFYDENRVRVRVYNKDCVDTQTMGYTYQPSKTPWVSRRPTASPSNVNIIERA
TNNGVKKVEDQAVLPLTLTEKTPVSVLVSRSKTSPEDKKKENEMVCFSGIKYDCEKFVR
FNVIVNDPGEKQVDKINAAAGQFVGSFAQLPHNHGGVDTKMSTSVEFGITELLEDTKAE
QTDSILVTIVPVIGCEGVIITDINVKVVKAEGETESETPIPQTDIEF

>to-CBZ41490.1 polyphenol oxidase precursor [Taraxacum officinale]

MPSLSPSPTTTTTPTASRDGRSFSSSSNYCSSFSFNSTQVPIARIPNHRHALACKTRGVDD
HHEIDSDKVDRRNILLGLGGLYGAAASFGSNSLAFADPIMAPDVTKCGPPDLPKGAIRTN
CCPPSTTKILDFKLPSPSNTLRIRPAAHLVDKDYITKFNKAIKLMKALPNDDPRSFKNQAD
VHCAYCDGAYDHFGFQDLDLQVHASWLFLPFHRYYIYFFEKICGKLINDPTFAIPFWNW
DAPDGMTIPDIYTNKKSSLYDALRDAKHQPPSLVDLDYNGIDENLNPSKQTSTNLKILYR
QMVSSSKTASLFMGNPYRAGDEANPGAGSIENIPHDPVHIWTGDRSQRNGEHMGNFYS
AARDPIFYAHHANIDRMWSIWKTLGGRRKDFTDKDWLDSSFSFYDENADLVRVKVRD
CLDSKNLGYVYQDVKIPWLKTKPSSKSEKSRRKAEHHKRDVARADEHIPFAKDVFPASL
DKVIKVLVPRPKKSRNKKQKEEEEEILVVEGIEVKSDLFVKFDVFVNDEDDGVRATADK
TEFAGSFVNVPHNHKHGKNVKTRLRLGISELLEDLGAEDDDNVLVTLVPKTKGGEVSIK
RIKIEHEN

>to-CAQ76694.1 polyphenol oxidase [Taraxacum officinale]

MASLAPSPTTATTGGRRFSSSSTYSSSFSFKSSQVPRARIPHHRHAVSCKTQNDDQHHEN
SGKVDRRNMLLGLGGLYGAAATFGSNPLAFADPIMAPDLTKCGPADLPQGAVATNCCP
PFTKNIIDFKLPPPSTNLRVRPAAHLANKDYIAKFNKAIELMKALPDDDPRSFKQQAAVH
CAYCDGAYDQVGFPDLDLQVHNSWLFLPFHRHYLYFFEKICGKLINDPNFAIPFWNWD
TPDGMTIPAIYANKNSQLYDALRDGKHQPPSLVDLDFNGEDENLSKSKQTSTNLTIMYR
QMVSSSKTASLFMGSPYRAGDEANPGGGSLESIPHGPVHIWTGDRNQPNGEDMGNFYS
AARDPIFYAHHANIDRMWSVWKTLGGRRNDFTDKDWLDSAFLFYDENAELVRVKVRD

ALDSKKLGYVYQDVGIPWLETKPEPRLKRALSKIKKKLAVARADEHIPFAKDVFPATLD
KVIKVLVPRPKKSRSKKQKDAEEEILVIEGIEVKRDEFVKFDVFVNDEDDGMRATADKT
EFAGSFVNVPHKHKHGKSVKTRLRLGISELLDDLGADDDDNVLVTLVPKNKGDEVSIK
GMKIEYED

>to-CCE45701.1 polyphenol oxidase precursor [Taraxacum officinale]

MAPLSFTLPTATTSYSPFFSQTAGNQRTLKKHAKQTHRFQVSCNVSSEDHEKPLPTYTQP
QKLILPQTSLDRRNLLLGLGSVYSTVNMPSAFATPIASPGFDLPLLKPSCRDATSGFNDTT
KELLRSKKCCPPASKKYPERRYKIDEITGPTRTRKPIHNNPECVKKYMDALTIMRGLPDD
DPRSFTNQAKIHCAYCNGSYMQNGQEIQIHNSWLFFPFHRWYLFFYERILGDLIEDPHFA
LPYWNWDNPNGGMVTPQLFVNEMFVNEENGKSEPNSLFDEYRNPGHLGSGLIDLDYQ
GQDRTDGFDQKEIHLATMNRQMMRNAFDATSFFGGKYVAGNEPIQRGDNVVGSVEAG
CHTAVHRWMGKPDPLRNGEDMGNFYSAGYDPLFYVHHANVDRMWTLWKEMGGKEP
DDIDWGNASYVFYDEKQEPVRVFNRDCADLTNLKYNYKESPTPWTERPPRSRCFRPKL
EIRTTTKVYTDIKDVKFPLSLTETKWLQVPRPAAFINRDAEQKKKEKEILCINGIMFDGTK
PIKFDVLVNECDAEYFTPCDSENVGSFAAIPHAKGGAMGCSSGMRFSLSELLEETGAEG
KDSINVTFIVPKATDNNKATDNNIATIKNIEIRLIPVLEKLSKGSKA

>to-CBZ41492.1 polyphenol oxidase precursor [Taraxacum officinale]

MESFAPTNNGTGISSTGGRSFYSSSSTYSASFSFTSSQVPIARIISTPRHAISCKTQDDHDHR
HANSGKIDRRNVLLGLGTLSGATATFGPNSLAFADPIMAPDITLCGPATFPEGVATTNCC
PPSTTKILDFKVPSQANTLRVRPAAHLVNKDYIAKYNKVVQLMKALPEDDPRNFMQQA
SVHCAYCDGTYDQLGFQDLEFQVHASWLFYPFHRYYLYFFEKICGKLIDDPNFTIPFWN
WDAPDGMTIPYIYTNKNSSLYDSLRDAKHQPPSLVDLDYNGVDRNLSPSKQTSTNLNIM
YRQMVMASKTTSLFLGSPYRAGDDPSPGGGIMENVPHTPVHNWTGDRSQPNVEDMGN
FYSAGRDPIFYAHHANVDRMWSIWKSLVGERKDYTDTDWLDSLFLFYDENADLVRVK
VRDCLDSKNLGYVYQDVDIPWLNSKPYPRLKKRLRAYKNVLFAKDVFPGSLDKLIKVM
VPRPKKLRSQEENGHDEVEEEEILVIEGIEVKMDKFVKFDVLVNEDDEKKATADKTEFA
GSFVNIPHKHKPGKNMKTRLRLGISELLKDLGAEDDKNVLVTLVPKTKGGEIRIDEIKIE
YGN

>to-ABX09994.1 chloroplast polyphenol oxidase [Taraxacum officinale]

MASLAPSPTTTLTPTGGRSFSSSSNYSFSFSFNSTQVPTPKTLNHRHAVTSCKSADDHHH
DIDSGTLDRRNVLLRLGGLYGAAATFGSNSLAFADPIMAPDLTQCGPADIPAGGIVTDCC
PPFTTKIQDFKLPPPSNTLRIRPAAHLVDKDYIDKFNKAIGLMKALPDDDPRSFKQQAVV
HCAYCDGAYEQVGYPGLDLQVHNSWLFFPFHRCYLYFFEKICGKLINDPTFAIPFWNWD
AADGMTIPDIYTNSKSPLYDTLRDAKHQPPSVVDLDYNGVDENLSPSEQTSTNLTIMYR
QMVSSAKTASLFMGSPYRAGDEPNPGGGTLENIPHGPVHIWTGDRNQPNGENMGNFYS

AGKDPIFYAHHANVDRMWSIWKTLGGRRKDFTDSDWLDSSFSFYDENAEFVRVKVRD
CLDSKKLGYAYEDVKIPWLKTKPRSKYGKSRRKAVGHKHAVARADELIPFAKDVFPAS
LDKVMIKVLVPRPKKSRSKKQKDEEEEVLVVEGIEVKRNEFVKFDVFVDDDDDEVRAT
ADKTEFAGSFVNVPHVHKHEKNVTTRLRLGISELLDDLGADDDDNVLVTLVPKTEGGE
VSIGMIKIEYED

>ls-XP_023741652.1 polyphenol oxidase I, chloroplastic [Lactuca sativa]

MELSRTDLTPTTMMASYIFSTVPSATEVTTNNFSHSSIFSKTSTHRFKYTHENQTHRFKVS
CNKTSDDKYDTLETSLDKKNVDRRNLLLGLGGTLYGAANLTFLPSAFSVPIAAPNVSDC
AIASKGIHNIKDAVRGVACCPPVLTLNSPKNYVFPKETAVRIRPAAQRASDDYIDKYKA
AIKAMRDLPDDHPHSFKQQAKIHCAYCNGSYTQKESGKEYEHLTLQIHNSWLFFPFHR
WYLYFYERILGKLIDDPTFAIPYWNWDNPTGMIIPDLFEKPIQVRERKENPVFDAYRDAR
HLPPALVDIDYNGEDRGVSCIDQITINLSAMYKQMISNASDPTSFFGGRYVAGMDHDDK
NSHGNPSVGSIEAGCHTAVHRWVADPRMPNNEDMGNFYSAGYDPIFYAHHANVDRM
WKIWKELGIRGHREPTDKDWLDASYVFYDENEELVRVYNRDCVDLNKLNYDYETSRIP
WARNRPIPRAKNPQMAARSARMGRSFHDVQFPVKLDGIVKVLVKRPYVNRTKEEKEK
ANEILMLNGICFDSEKFVKFDVYVDDKDDEPETTAADSEFAGSFAQLPHHQSGEKMFMT
SAARFGLTELLEDIEAEDDESIMVTLVPRTGSDDITISEIKIELVPIV

>ls-PLY90871.1 hypothetical protein LSAT_9X102320 [Lactuca sativa]

MASLALSSLPTSTTTKKPLFSKTSSHVKPFHRFKVSCNAPADNNDKTVNNSDTPKLILPK
TPLETQNVDRRNLLLGLGGLYGAANLTTIPSAFGIPIAAPDNISDCVAATSNLRNSKDAIR
GLACCPPVLSTNKPMDYVLPSNPVIRVRPAAQKATADYIAKYQQAIQAMKDLPEDHPHS
WKQQGKIHCAYCNGGYNQEQSGYPNLQLQIHNSWLFFPFHRWYLYFYEKILGKLINDP
TFALPYWNWDNPTGMVIPAMFEQNSKTNSLFDPLRDAKHLPPSIFDVEYAGADTGATCI
DQIAINLSSMYRQMVTNSTDTKRFFGGEFVAGNDPLASEFNVAGTVEAGVHTAAHRWV
GNSRMANSEDMGNFYSAGYDPLFYVHHANVDRMWQIWKDIDKKTHKDPTSGDWLNA
SYVFYDENENLVRVYNRDCVDINRMGYDYERSAIPWIRSRPTAHAKGANVAAKSAGIV
QKVEDIVFPLKLNKIVKVLVKRPATNRTKEEKEKANELLFVNGITFDAERFLKIDVFVND
VDDGIQTTAADSEFAGSFAQLPHNHGDKMFMRSGAAFGITELLEDIEAEGDDSVVVTLV
PRTGCDEVTIGEIKIQLVPIV

>ls-PLY67749.1 hypothetical protein LSAT_9X102021 [Lactuca sativa]

MRDLPDDHPHSFKQQAKIHCAYCNGSYTQKESGKEYEHLTLQIHNSWLFFPFHRWYLY
FYERILGKLIDDPTFAIPYWNWDNPTGMIIPDLFEKPIQVRERKENPVFDAYRDARHLPPA
LVDIDYNGEDRGVSCIDQITINLSAMYKQMISNASDPTSFFGGRYVAGMDHDDKNSHGN
PSVGSIEAGCHTAVHRWVADPRMPNNEDMGNFYSAGYDPIFYAHHANVDRMWKIWK
ELGIRGHREPTDKDWLDASYVFYDENEELVRVYNRDCVDLNKLNYDYETSRIPWARNR

PIPRAKNPQMAARSARMGRSFHDVQFPVKLDGIVKVLVKRPYVNRTKEEKEKANEILM
LNGICFDSEKFVKFDVYVDDKDDEPETTAADSEFAGSFAQLPHHQSGEKMFMTSAARF
GLTELLEDIEAEDDESIMVTLVPRTGSDDITISEIKIELVPIV

>ls-XP_023756514.1 polyphenol oxidase I, chloroplastic [Lactuca sativa]

MMASLALSSLPTSTTTKKPLFSKTSSHVKPFHRFKVSCNAPADNNDKTVNNSDTPKLILP
KTPLETQNVDRRNLLLGLGGLYGAANLTTIPSAFGIPIAAPDNISDCVAATSNLRNSKDAI
RGLACCPPVLSTNKPMDYVLPSNPVIRVRPAAQKATADYIAKYQQAIQAMKDLPEDHP
HSWKQQGKIHCAYCNGGYNQEQSGYPNLQLQIHNSWLFFPFHRWYLYFYEKILGKLIN
DPTFALPYWNWDNPTGMVIPAMFEQNSKTNSLFDPLRDAKHLPPSIFDVEYAGADTGA
TCIDQIAINLSSMYRQMVTNSTDTKRFFGGEFVAGNDPLASEFNVAGTVEAGVHTAAHR
WVGNSRMANSEDMGNFYSAGYDPLFYVHHANVDRMWQIWKDIDKKTHKDPTSGDW
LNASYVFYDENENLVRVYNRDCVDINRMGYDYERSAIPWIRSRPTAHAKGANVAAKSA
GIVQKVEDIVFPLKLNKIVKVLVKRPATNRTKEEKEKANELLFVNGITFDAERFLKIDVF
VNDVDDGIQTTAADSEFAGSFAQLPHNHGDKMFMRSGAAFGITELLEDIEAEGDDSVV
VTLVPRTGCDEVTIGEIKIQLVPIV

>ls-XP_023741650.1 polyphenol oxidase I, chloroplastic [Lactuca sativa]

MMASLSLSTLPTSTPTKKPLFSKTSSHVKQSHRFKVSCNSAANNNEKTVKNSETPKLILP
KTPLEMQNVDRRNLLLGLGGLYGAANLTSIPSAFGTPIAAPDNISDCVTASSNLQNAND
AVRGLACCPPVLSTDKPKDYVLPTNPVLRVRPAAQRATDEYIVKYKAAIQAMKNLPDE
HPHSWKQQAKIHCAYCNGGYNQEQSGFPDIQLQIHNTWLFFPFHRWYLYFYERILGKLI
NDPTFALPYWNWDNPTGMVLPAMFETDGKRNPIFDPYRNATHLPPAIFEVGYNGTDSG
ATCIDQISANLSLMYKQMITNAPDTTTFFGGEFVAGDDPLNKEFNVAGSIEAGVHTAAH
RWVGDPRMANSEDMGNFYSAGYDPLFYVHHANVDRMWKIWKDLGIKGHTEPTSTDW
LDASYVFYDENEELVRVYNRDSVNMTAMGYDYERSEIPWLHSRSVPHTKGANVAAKL
VGIVKKVEDVTFPLKLNETVKVLVKRPTKKRNKKNKQEANEMLFLNKIKFDGEEFVKF
DVFVNDVDDGVETTAAESEFAGSFSQLPHGHKHGTKMSMTSGAAFGLTELLEDIEAED
DDSILVTLVPKIGCDDVTVGEIKIKLVPIV

>ls-XP_023765720.1 polyphenol oxidase I, chloroplastic [Lactuca sativa]

MASFQLVNPFASTTRKLPDSTSSRRLKTHPQKNHRFKVSCNVAQDGNEKLLLVPDSKNL
ILPKPSLDTLNVDRRNLLLGLGGLYSTVNFTSLPAAIAAPITTPDISTCIPSEQGFNVQDSV
RSNQCCPPMMTTTPKDFVFPKDKTIRVRPAAHRATPEYIAKYKAAIQAMKDLPDDHPHS
FVQQAKIHCAYCNGGYTQVASGYADKQLQIHNSWLFFPFHRWYLYFYERILGKLIDDPT
FALPYWNWDNPAGMSFPAFFETDGKRNPVFDAFRNVNHVSPETVVDLDYNGSDSGAP
CLQQISTNLAAMYKQMISNATDPLSFFGGEFRAGDDPFGNSDPSVGSIEAGCHTAMHRW
TGNPRMPNNEDMGNFYSAGYDPAFYVHHANVDRMWKVWKDLGIKGHTEPTDPDWL

NASYVFYDENEELVRVYNKDCVQTENLKYDFELSPLPWLKNRPVAHTKPETTTKPVEK
VKVPDVKFPIKLDKIQKVLVKRPAKNRSQSEKEKATEQLLIKGIKFNVSKFVKFDVFVND
QDDVPTSSASESEFAGSFAQLPHHHGGHKKLMTSAARFGLTELLEDIGAEDDEYILVTLV
PKVGAEDLTVDEIKVELVPIV

>ls-XP_023765834.1 polyphenol oxidase I, chloroplastic [Lactuca sativa]

MSSFQSLATFTSITTRTLPNSPSNRRSNSYPKQTHRLKVSCNVAPEDNEKLLVVPETQKLI
LPKTSLDTLNVDRRNLLLGLGGLCTTVNFTSIPTAFGRPITAPDISSCRASTDGLDLKNAI
RTNACCPPNLSKKVKDFVFPNDKSLRIRRAAHKAPEDYITKYKAALKAMRALPDDHPH
SFVSQAKIHCAYCNGGYTQIATGDSDKIIQIHNSWLFFPFHRWYLYFYERILGKLINDPTF
AIPYWNWDNPAGMTLPAFFEEGNNRKEKLENPAFDAFRNTSHFAPTIVELDYQGEDSG
APSAKQININLTQMNSQMIRNAHDTRSFFGGKYVAGSDPIPNGDRCVGSIEAGCHTAIHR
WVGDSRTFNNEDMGNFYSAGYDPLFYVHHANVDRMWVEWKGLDKRNKEPKDEDWL
NASYVFYDENEELVRVYNKDCVRNDKLRYAYEFSPLPWLNNRPTPRTLKSKIALKSVG
TVKQVEDTKFPLKLDKITKVLVKRPATNRSQEEKEKAVELLLIKDVKYNGGKFVKFDVF
VNDQDDVRASSAEESEFAGSFAQLPHGPGDDMLMTSGARFGLTELLEDIQAEDDELILV
TLVPKAGCEEVTVGEIKVELVPLDD

>ls-XP_023766357.1 polyphenol oxidase I, chloroplastic [Lactuca sativa]

MFSFQSFATFTSITTRTLPNSTSDRRYNSYPKQIHHLQISCNVAPDDKEKLVVVPETQKLI
LPKSSLDTLNVDRRNMLLGLGGLYTTVNFTSPAAFAAPITTPNFSTCVTSNLGFQDPNKA
VRSRACCPPAPATSTAPKDFVFPKDQVIRIRPAAHRTTTEYVAKYKAAIQAMRDLPDEH
PHSFVAQAKIHCAYCNGGYTQIASGFPDKELQIHNSWLFFPFHRWYLYFYERILGKLIDD
PTFALPYWNWDHPNGMTFPAFLEDDSAFDAYRNRKHLPPALVDLNYSGSDRHATCIRQ
ITSNMTLMYKQMISNAGDTTSFFGSEYRAGNDAYRNGDPSVGSIEAGCHTAVHRWMG
DPGMPNNEDMGNFYSAGYDPAFYIHHANVDRMWKLWKDMGIKGHSEPTHLDWRNAS
YVFYDENEQLVRVYNKDCVSLEKLKYDYEYSPPLWKISRSSIRRTLPEPIPYNMKSAETV
KQLPDVKFPLKLDKITKVVVKRPAKSRSQEDKEKANELLLIKGIKFNSDKFIKFDVFVNG
QDDVSESFEEESEFAGSFAQLPHNHGDDMLMKSGIRFGLTELLEEMEAEDDEFILVTLVP
KVWFEEVTIDEIKVELVPII

>ls-XP_023738685.1 polyphenol oxidase I, chloroplastic [Lactuca sativa]

MASLSSTLATVIPSSPEFSKTATKRRLKTHAKRTHRFQVSCNDNEKPPTTNPQPEKLILPP
ETSLNRQNVDRRNLLLGLGGLYTTTNLNTLPPVFANPIKAPSFVPGCTDSLWNLDFESGV
RTGACCPPAAKELEMDYKFPTTGETRIRQPVHRASPEYIQKFKDAMQKMKALPDDDPC
SFKNQAKVHCAYCNNSYTQMASGYPEKVLQVHYSWLFFPFHRWFLYFFERILGDLIED
KTFGLPYWNWDNPAGMEIPAVFEDGGRSNPLYNSYRNVNHLRPAVIDLDYRMKERNIS
PLDQVRINLCIMNRQMKRNASDPTSFFGGEYVAGDNPISSVGSIEAGCHTAVHRWVGDP

RTPNEEDLGNFYSAAYDPLFYVHHANVDRMWTLWKGLGGEGRKEPNDKNWEEASYV
FYDENRKPVRVYNKHCVNLEDLKYEYEDSETPWKSNPPKPRSATYSETTSPENVTEMEF
PLSISETVTVQVKRPETNRPKDQKKTIKEILLLKGINFNGGKFVKFDVFVNIFEDINRISPC
ESQYAGGFGLLPHKTSEKMNSKTGVRIELTELLEEINADGDDSVQVTIVPRVGCDDVTFE
DIKIELIDVYEKESN

>ls-XP_023741689.1 polyphenol oxidase I, chloroplastic [Lactuca sativa]

MASFSFYTLPTSTSTIKNPLFSKSSSHVKHSHRFRSSCKAAADSNDKYVENPDTPKLILPK
SPSLDTQNVDRRNLLLGLGGLYSAANFTSIPSAFGVPIEAPDINISKCVTATVRGVSAEAI
RGLTCCPPVFDSSAKPAPYEFPDNQVIRMRPAAQRVSADYKKDFRKAVEIMKGYNDND
PHSWTQQAKVHCAYCNGAYTQVKSGLEFEKYIIQVHNSWLFFPFHRWYLYFLEKIMGK
ALGDDTFALPYWNWDHPTGMTIPAMYEDKLKNPDGNVDTPENTRFNSLFDPLRNTSHI
APALIDFQYYPQKQEVYNCADQIEINLSIMYNQMIANALDTKSFFGGELVAGENPNENK
KAGSIEDGVHTIAHQWVGNNRLKNGEDMGNFYSAGYDPLFYGHHANVDRMWKIWKG
MNRRHHAPSSTDWLDASYVFYDENRKLVRVYNRDCVDTRTMGYDYERSEIPWIRNRP
NPHPKGGKDKGNARKPDKATVKDLSFPVRLNQTLEVRVMRPAKRTTEDKERTEIAIEKL
VLQGVRYDCERFVKFDVIMNDPDNGVDVTPVDTEFLGYFSRLPHGMVAENRMKEISGI
SFAIKDRLKILKVENDDSIVVKIVPRAGCEDVTIQNIEVVMDPVDNIVPLAESLVVQDRNS
DELTLEGPTALDSNSDDSGSE

>ls-XP_023763958.1 polyphenol oxidase I, chloroplastic [Lactuca sativa]

MASLPTPTVTAAGATTKTYSSSFTTTSPVISSWPLFSKKCALNKPLKHKISCNAGSSENSL
NNLDRRNVLLGLGGLAGAVNLTSVPSVGAAPISAPDISKCGTNPLSGFRPGESTPTGGDC
CPPDSPQIMDFKFPKNEAFRVRPAAHLLSPKYIAKFNEAIKRMKELPETDPRNFLQQAHI
HCAYCNGAYTQSSSGFPDIEIQIHNSWLFFPFHRWYLYFYERILGSLIDDPTFALPFWNW
DTPAGMTIPKYFNDPKNAVFDPKRNQGHLQGVVDLGYNGKDSDTTDIEKVKNNLAIMY
RQMVTNATDPTAFFGGEYRAGIEPISGGGSVEQSPHTPVHRWVGDPRELNGENLGNFYS
AGRDTLFYCHHSNVDRMWSLWKMQGGKHKDITDPDWLNTSFVFYDENKNLVRVYVK
DCLYTNQLGYDYQRVDVPWLKSKPVPRAPRSGVARKSIGKVKQAKEVSFPVKLDKTV
KVLVARPKKSRSKKEKEDQEELLIVQGITYDSEKYVKFDVYVNDEDDDASAPDQTEFA
GSFAQLPHKHKGKTMSKTNFRAGLTELLEDLEADDDDNVLVTIVPRSGSEDITIDNIKIIY
A

>ls-PLY90866.1 hypothetical protein LSAT_9X102220 [Lactuca sativa]

MKTHGKQTHRFQVSCNVSSNNHEKPLPKNPQPQKLILPQTSLDLQNVDRRNLLLGLGG
VYSTATLSGLPPAFAEAIKAPFNQPDRPCKDAVSGFDINKKLLRPIDCCPLSKNGPESHFK
FPDKSSKTRIRYPLHKLPVGYLDKYMDAIQKMKDLPDSDPRSFNNQAKVHCAYCNGSY
TQNGQELQIHNSWLFFPFHRWYLYFYERILGDLIGDSTFGLPYWNWDNPEGMTIPHFFV

EKQCNNYKFENGENPLYDKYRDESHLRYELVDLDYSGRNRDLCYDQKEINLATMNRQ
MMRNAFDATSFFGGKYVAGDEPIPRGDNVVGSVEAGCHTAVHRWVGNPDPKGNKED
MGNFYSAGYDPLFYVHHSNVDRMWTLWKQMGGKEPTDTDWENASYVFYDEKQNPV
RVYNKQSVDLSNLKYEYHSSATPWTDRPPRSRCNRPGYPKRNNTKDFPNQKDPPEALT
LTDSTVRLRVKRPPASKNRNAEQKKSEKEILCLIGISFDCTEAAKFDVFVNDCDEEQITPC
DSENVGSFAAVPHAKGMAMGCKSGMRFSLTELLEETKAEGDESIRVTIVPRTTPGKKVK
VTIDAIEIRLIPVLEK

>ls-XP_023756515.1 polyphenol oxidase I, chloroplastic [Lactuca sativa]

MASLSFTLATPTTSSSPFFSQTTTKQRRLMKTHGKQTHRFQVSCNVSSNNHEKPLPKNPQ
PQKLILPQTSLDLQNVDRRNLLLGLGGVYSTATLSGLPPAFAEAIKAPFNQPDRPCKDAV
SGFDINKKLLRPIDCCPLSKNGPESHFKFPDKSSKTRIRYPLHKLPVGYLDKYMDAIQKM
KDLPDSDPRSFNNQAKVHCAYCNGSYTQNGQELQIHNSWLFFPFHRWYLYFYERILGD
LIGDSTFGLPYWNWDNPEGMTIPHFFVEKQCNNYKFENGENPLYDKYRDESHLRYELV
DLDYSGRNRDLCYDQKEINLATMNRQMMRNAFDATSFFGGKYVAGDEPIPRGDNVVG
SVEAGCHTAVHRWVGNPDPKGNKEDMGNFYSAGYDPLFYVHHSNVDRMWTLWKQM
GGKEPTDTDWENASYVFYDEKQNPVRVYNKQSVDLSNLKYEYHSSATPWTDRPPRSRC
NRPGYPKRNNTKDFPNQKDPPEALTLTDSTVRLRVKRPPASKNRNAEQKKSEKEILCLIG
ISFDCTEAAKFDVFVNDCDEEQITPCDSENVGSFAAVPHAKGMAMGCKSGMRFSLTELL
EETKAEGDESIRVTIVPRTTPGKKVKVTIDAIEIRLIPVLEK

>ls-XP_042754290.1 polyphenol oxidase, chloroplastic [Lactuca sativa]

MASLSFTLAMATTPSSSPFFSKPANQRQLIKTHAKQTHRFQMSCNVPSDDHEKPVDRRN
LLLGLGGLYSAVNLTGLPSAFADPITTPSFNPNCRDAGTGFDVKKGLLRTTACCPPESKK
GPEKQFEFPKHDEIRIRYPIHCAPEGYMNKFKEAMRLMRALPDDDPRSFKNQAKIHCAY
CNGSYTQMATGSQQELLIHFNWLFFPFHRWYLYFFERILGELIGDPTFGLPYWSWDERE
GMKIPPTFREGGESNPLYDIYRNNIRNYEAIVDLDFNGKDREDTTDDYQIKINQHAMYR
QMMRNAFDTKSFFGGKYVAGNTPIDAKDSSVASIEAGCHTAIHRWVRDPGSPNGEDMG
NFYSAGYDPLFYVHHSNVDRMWALWKEMGESNRDPIHPDWLNASYVFYDEKQNPVR
VYNKQCVDMEKLKYKYHGPEIPSWVNSRPKPKCSASERSQIDITSATKDVKNRTLTNVD
TFVLVRPETARTRTVDESEIEVLTLNNISFNGNKAVKFDVLVNACNIDTNKFTPADSEYA
GSFATVPHNHDMKISTTFRFPLRELLKDIGAEGNTAIQVTIVTQEKETENISIGEIKIEDYSL
AEISKASLPTGLQGAGANVGVDDLTE

>ls-PLY76929.1 hypothetical protein LSAT_9X1560 [Lactuca sativa]

MASLSFTLAMATTPSSSPFFSKPANQRQLIKTHAKQTHRFQMSCNVPSDDHEKPIINTPQ
HQKLILPKTSLDMQNVDRRNLLLGLGGLYSAVNLTGLPSAFADPITTPSFNPNCRDAGT
GFDVKKGLLRTTACCPPESKKGPEKQFEFPKHDEIRIRYPIHCAPEGYMNKFKEAMRLM

RALPDDDPRSFKNQAKIHCAYCNGSYTQMATGSQQELLIHFNWLFFPFHRWYLYFFERI
LGELIGDPTFGLPYWSWDEREGMKIPPTFREGGESNPLYDIYRNNIRNYEAIVDLDFNGK
DREDTTDDYQIKINQHAMYRQMMRNAFDTKSFFGGKYVAGNTPIDAKDSSVASIEAGC
HTAIHRWVRDPGSPNGEDMGNFYSAGYDPLFYVHHSNVDRMWALWKEMGESNRDPI
HPDWLNASYVFYDEKQNPVRVYNKQCVDMEKLKYKYHGPEIPSWVNSRPKPKCSASE
RSQIDITSATKDVKNRTLTNVDTFVLVRPETARTRTVDESEIEVLTLNNISFNGNKAVKFD
VLVNACNIDTNKFTPADSEYAGSFATVPHNHDMKISTTFRFPLRELLKDIGAEGNTAIQV
TIVTQEKETENISIGEIKIEDYSLAEISKASLPTGLQGAGANVGVDDLTE

>ls-XP_023763394.1 polyphenol oxidase, chloroplastic [Lactuca sativa]

MASLAQSPTTTTTTGGRCFSSSSTYSSSFSFKSSQVPIARITNHRHAVSCKGALDDDDHH
HENSGKFDRRNVLLGLGGLYGAAATFGSNSLAYAAPIMAPDLTKCGPADLPQGAVPTN
CCPPYTTKIHDFKLPPPSTTFRVRPAAHLANKDYIAKFNKAIELMKALPDDDPRSFKQQA
AVHCAYCDGAYDQVGFPDLELQVHGSWLFLPFHRYYLYFFEKICGKLIDDPNFAIPFWN
WDAPDGMKIPDIYTNKKSPLYDALRDAKHQPPSLIDLDYNGDDENLSRSKQTSTNLTIM
YRQMVSSSKTASLFMGSPYRAGDEASPGSGSLESIPHGPVHIWTGDRNQQNGEDMGNF
YSAARDPIFYAHHANIDRMWSVWKTLGGRRNDFTDKDWLDSSFLFYDENAEMVRVKV
RDCLDSKKLGYVYQDVEIPWLKSKPEPRLKRALSKIKKLAVARADEHIPFAKDVFPASL
DKVIKVLVPRPKKSRSKKQKEDEEEILVIEGIELKRDEFAKFDVFVNDEDDGMRATADK
TEFAGSFVNVPHKHKHGKNVKTRLRLGISELLEDLGAEDDDNVLVTLVPKNKGGEVSIK
GIKIEHED

>ls-PLY67794.1 hypothetical protein LSAT_0X6520 [Lactuca sativa]

MGNFYSAGYDPLFYGHHANVDRLWSIWKGMDRKGHKDPTSIDWLDASYVFYDENEEL
VRVYNRDCVDTRRMGYKYERSAIPWIESRPTPHAKGANVAVNAVASGIVPKVENLTFP
LTINKTFEVLVPRPAKNRTNADKEKANELLMINGIKFDCERFFKFDVIVDDLDDGVEVTA
ADSEFAGSFAQLPHGDSDEKMLMTSGASFGITELLEDIEAEGDDSILVKIVPKEGCDDVTI
SNIKVVLVPSE

>ls-XP_023763395.1 polyphenol oxidase, chloroplastic [Lactuca sativa]

MASFSPSQATSYTSGRRFSSSSTYSSSFSFKSSQVPIARISKHRHAVSCKTLDDDHHHHAN
SGKLDRRNVLLGLGGLYGTAANFGSNSLAFADPIMGPDLSKCGPAELPQGAIPTNCCPPF
TTKIIDFKLPPQSNPLRVRPAAHLVDKDYIDKFSKAIELMKALPDDDPRSFKQQANVHCA
YCDAAYVQLGYPDVELQVHNSWLFFPFHRCYLYFFEKICGKLIDDPTFAIPFWNWDAPV
GMKIPDIYTDKNSSLYDTLRDAKHQPPTVVDLDYNGFDNNLSPSEQTSTNLTIMYRQMV
SNAKTASLFMGSPYRAGDDPSPGAGSLESVPHNPVHIWTGDRNQPNGEDMGNFYSAGK
DPIFFAHHGNLDRLWSVWKTLGGRRKDFTDNDWLDSSFLLYDENAELNRVKVRDCVD
SKNMNYVYQDVELPWLESKPVPRLQKASRNIKKHAHEHIPFAKDVFPASLDKVIKVRVP

RLKKSRTKKQKEEEEEILVIEGIEVKRDEFVKFDVLVNDDDDGTQATAAKTEFAGSFAS
VPHMHKHGKNWKTKLRIGITDLLEDLKDEEDHNVLVTLVPKTSGGDISIGGIKIEHEEC

>ls-PLY73479.1 hypothetical protein LSAT_2X43561 [Lactuca sativa]

MAPDVTKCGPAELPIGVEPINCCPPITTNILDFKPPQSNTLRVRPAAHLVVNEDYIAKFNK
AIELMKALPHDDPRNFMQQANIHCAYCDGSYKQVGFPNLEHQVHTSWLFFPYHRYYV
YFFEKICGKLIDDPNFAIPFWNWDAPDGMQIPDIFTNKNSQLYDPLRDTNHQPPSVVDLD
FNGVDKNLSRSELTSKNLSVMYRQMVSSAKTASLFMGRPYRAGDEPNPGSGSIEVSPHA
PVHSWTGDPKQKNWEDMGTFYSAARDPIFYAHHANIDRMWSIWKTLGGKDFTDKEWL
DSSFVFYDENADLVRAKVRDCCDSKNLGYVYQDVDIPWINCKPYRPSERAPVSYDHIPY
TKNVFPGNLDKVIKVLVPRPKKSRSKKEKEEEEEILVIDGIEVKTDEFVKFDVLINCEDDG
MSATADQLEFAGSFVNVPHNHDHGKNMKTRLRLGISELLEDLTADNDEKVLVTLVPKT
NGSGISIQEIKIEYEVR

>ls-XP_023734197.1 polyphenol oxidase, chloroplastic [Lactuca sativa]

MASPAASPTKTPTATTSTFTGGRSLSSSFTYSSSFSFKSSQVPIARISNHRHAVSCKTIDDD
NHHANSSKFDRRNILLGIGGLYSATTTFGSNSLAFADPVMAPDVTKCGPAELPIGVEPIN
CCPPITTNILDFKPPQSNTLRVRPAAHLVVNEDYIAKFNKAIELMKALPHDDPRNFMQQA
NIHCAYCDGSYKQVGFPNLEHQVHTSWLFFPYHRYYVYFFEKICGKLIDDPNFAIPFWN
WDAPDGMQIPDIFTNKNSQLYDPLRDTNHQPPSVVDLDFNGVDKNLSRSELTSKNLSV
MYRQMVSSAKTASLFMGRPYRAGDEPNPGSGSIEVSPHAPVHSWTGDPKQKNWEDMG
TFYSAARDPIFYAHHANIDRMWSIWKTLGGKDFTDKEWLDSSFVFYDENADLVRAKVR
DCCDSKNLGYVYQDVDIPWINCKPYRPSERAPVSYDHIPYTKNVFPGNLDKVIKVLVPR
PKKSRSKKEKEEEEEILVIDGIEVKTDEFVKFDVLINCEDDGMSATADQLEFAGSFVNVP
HNHDHGKNMKTRLRLGISELLEDLTADNDEKVLVTLVPKTNGSGISIQEIKIEYEVR

>ls-PLY79310.1 hypothetical protein LSAT_0X46281 [Lactuca sativa]

MAPDLSKCGPADLPEGAVSTDCCPPYTTKILDFKLPPPSNTFRVRPAAHLANEDYIGKFN
KAIELMKALPDDDPRSFKQQANVHCAYCDGAYDQVGFPDLELQVHNSWLFFPFHRYY
MYFFEKICGKLIDDPNFAIPFWNWDAPDGMKIPDIYTNKKSSLYDPLRDVDHQPPSLIDL
DFNGVDENLSPSEQTSKNLTVMYRQMVSSSKTSTLFMGSPYRAGDDASPGSGSIENTPH
NPVHIWAGEWKHNNGKNMGKLYSAARDPLFYAHHGNIDRMWSVWKTLGGRRKDFTD
KDWLDSSFLFYDENAELNRVKVRDCLDTKNLGYVYQDVEIPWLKSKPVPRRTKPKQKP
KNKNNKQAVARADEYIPFAKDVFPASLNEVIKVLVPRPKISRSKKQKEEEEEILVIEGIEV
KIDEFVKFDVFVNDEDDGMRATADKTEFAGSFVNVPHTHKHGKNLKTRLRLGISELLED
LNAEDDENVLVTLVPKTRGSGISIAEIKIEHEE

>ls-XP_023771812.2 polyphenol oxidase, chloroplastic, partial [Lactuca sativa]

SKHRHAVSCKTLDDDHHHHANSGKLDRRNILLGLGGLYGTAATFGSNSPAIAAPIMAPD
LSKCGPADLPEGAVSTDCCPPYTTKILDFKLPPPSNTFRVRPAAHLANEDYIGKFNKAIEL
MKALPDDDPRSFKQQANVHCAYCDGAYDQVGFPDLELQVHNSWLFFPFHRYYMYFFE
KICGKLIDDPNFAIPFWNWDAPDGMKIPDIYTNKKSSLYDPLRDVDHQPPSLIDLDFNGV
DENLSPSEQTSKNLTVMYRQMVSSSKTSTLFMGSPYRAGDDASPGSGSIENTPHNPVHI
WAGEWKHNNGKNMGKLYSAARDPLFYAHHGNIDRMWSVWKTLGGRRKDFTDKDWL
DSSFLFYDENAELNRVKVRDCLDTKNLGYVYQDVEIPWLKSKPVPRRTKPKQKPKNKN
NKQAVARADEYIPFAKDVFPASLNEVIKVLVPRPKISRSKKQKEEEEEILVIEGIEVKIDEF
VKFDVFVNDEDDGMRATADKTEFAGSFVNVPHTHKHGKNLKTRLRLGISELLEDLNAE
DDENVLVTLVPKTRGSGISIAEIKIEHEE

>ls-XP_023763397.1 polyphenol oxidase, chloroplastic [Lactuca sativa]

MTSFASSPTKTLTGTASRSERRISSSSNYSSSFSFKSSQVPIARISKHRHAVSCKTLDDDHH
HHANSGKLDRRNILLGLGGLYGTAATFGSNSPAIAAPIMAPDLSKCGPADLPEGAVSTD
CCPPYTTKILDFKLPPPSNTFRVRPAAHLANEDYIGKFNKAIELMKALPDDDPRSFKQQA
NVHCAYCDGAYDQVGFPDLELQVHNSWLFFPFHRYYMYFFEKICGKLIDDPNFAIPFW
NWDAPDGMKIPDIYTNKKSSLYDPLRDVDHQPPSLIDLDFNGVDENLSPSEQTSKNLTV
MYRQMVSSSKTSTLFMGSPYRAGDDASPGSGSIENTPHNPVHIWAGEWKHNNGKNMG
KLYSAARDPLFYAHHGNIDRMWSVWKTLGGRRKDFTDKDWLDSSFLFYDENAELNRV
KVRDCLDTKNLGYVYQDVEIPWLKSKPVPRRTKPKQKPKNKNNKQAVARADEYIPFAK
DVFPASLNEVIKVLVPRPKISRSKKQKEEEEEILVIEGIEVKIDEFVKFDVFVNDEDDGMR
ATADKTEFAGSFVNVPHTHKHGKNLKTRLRLGISELLEDLNAEDDENVLVTLVPKTRGS
GISIAEIKIEHEE

>ls-XP_023741653.1 polyphenol oxidase, chloroplastic-like [Lactuca sativa]

MMASFNLYTVPAATTATTNIIKTNYYQLKTQAKQTHRLKASCNAIPDKNNDKALETSLQ
MINIDRRNIILRLGGLFVASNMTSVPLAYANAIAARSNHSVCAASPLGIQNLGTPVKEPM
ENREDVDTNKLGYEYKWSEIPWGRSQPTEYGKDSKFVDKSIGIEKKVGEVEFPVKLNKT
VKVLVKRPAVNRTKEDKQKANEILLVNGVRFDGEKYVKFDVFVNDIDNGTETTPADSE
FAGSFAQLPHGKTDRMMMMSGVRFGLTELLEDIKAEDDEYVLVKLVPRTGCDDVTVSE
IKIELVPVV

>ls-XP_023763391.1 polyphenol oxidase, chloroplastic [Lactuca sativa]

MAGIPTTPATFPMSLDKPTTVMVARPAKKEREKEEEEVLVIEGIEINRNEFVKFDVFINDE
DEETAAGGGAEKAECAGSFVNVPHKHRNGHSGDGGKVKKTQLRIGISELLEDLGVEED
DEDVVVKLVPRCENVHVTIGGIKIENE

>ls-PLY67759.1 hypothetical protein LSAT_0X6500 [Lactuca sativa]

MASLSFSTLPISTSTSTTKKPLFSTNSSHEKRSHRYKFSCTATADDNDKTVENSDTPKLILP
KSPSLDMQNVDRRNLLLGLGGLYSAANLTTIPLAFGIPIQAPNDISSCVAARSSIPNQKEA
LRGIACCPPKRSTRPPGRYNFRTDQAIRV

>ha-XP_021982569.1 polyphenol oxidase I, chloroplastic [Helianthus annuus]

MASISLSAVPTTSLSSRPPLFSKTSSTYCFKVSCKSSMDDHSENNPKLILPMQNVDRRNM
LLGLGSLYGAANLTNIGSALAYPITSPDNISNCVPAEDGFIPGDAVRGNACCPPTTQTTA
VPYVLPKFTKLRVRPAADRLSEEYIAKYQLAIKRMKELPDEDPRSWKQQGKIHCAYCN
GAYSQYMNGHPELKIQVHNNSLFFPFHRWYLYFYERILGSLIDDPTFGLPYWNWDNPK
GMLLPDMFEAPYPNPDPTKPPNPKFNPLFDPYRNCAHLPPAIVDLDTSSAAGDKSCAHQ
QISENLSAMYTQMIGTTTALPDAFFGTNYDPDPSKILAGRIESGVHTAVHIWVGNPRMPN
NEDLGNFYSAGYDPAFYVHHSNVDRMWQIWNELDFRNVNPCNDDWLNASFVFYDEN
QRLVRVYNKDCVDINKMGYTYEPSRLTWLQGRPIRRVKASNIAANSFGVVKKVEEIEFP
VKLDQAVKVLVKRPATKRNEDDKMKTSEMLFLEGIKYNGEQFFKFDVLVNDVDDGIET
TPVSSEFVGTFAQVPHSLGGNMVMTSGAAFGLTQLLEDLEAENDEYVLVTLVPRAGAE
DATISKINIQLVPLAN

>ha-XP_021993747.1 polyphenol oxidase I, chloroplastic [Helianthus annuus]

MASLSLSAVPTTTTTSLSSRSPLFSKSTHRSKVSCKSSADDHSENKLILPMQNVDRRNML
LGLGGLYGAANLTNIGSALAYPITAPENISNCVPADVGFNTEDAVRGSACCPPTTQTTAV
PYVLPEVTELRVRPAADRLTPEYIAKYQLAIKRMKELPDEDPRSWKQQGKIHCAYCNGA
YTQYMNGHPELKIQVHNNSLFFPFHRWYLYFYERILGSLIEDPTFGLPYWNWDNPKGM
LLPEMLEAPYPHPDPESNRPPNPRYNPLFDPYRNVAHLPPAIIDLDRGNAGGDKDCVAQI
SENLTAVYDQMIADSAANPDAFFGTNPNPDDPKAIVAGSIESGVHSAVHVWVGNPRMT
NNEDMGNFYSAGYDPAFYLLHSNVDRMWQIWNELDARNVNPSNDDWLNASFVFYDE
NQNLVRVYNRDCVDIERMGYTFEPSRLTWLQARSMPRNKASNIAAKSVGVVKKVEEIE
FPVKLDQTVKVLVKRPATNRSADDKKKASEMLFLEEIKYNGEQFFQFDVLVNDVDDGI
ETTRVSSEFAGTFAQVPHGLGGGHMLMSSGTAFGLTQILEDLEAEGDEYVLVTLVPREG
AEDATISNIKIKLVPLAN

>ha-XP_021993750.1 polyphenol oxidase I, chloroplastic [Helianthus annuus]

MASLSLSTVPTTITSLSSRSALFSKTSTHRFKVSCNGSSDERLTNSENNPIKPILPMQNNNV
DRRNMLLGLGGLYGAANFTSIGSALAYPITAPDNISDCVAATVGVTDPENAVRGVACCP
PSSQTTPAPYSLPDSTQLRVRPAAHRLTSDYIDKYRAAIAAMKALPDDDPRSWKQQGKI
HCAYCNGAYSQEMNDHKELKLQVHNSSLFYPFHRWYLYFYERILGSLINDPTFALPYW

NWDSPMGMLLPAMLETPVTDPSGKTERSDPKFNSLFDPYRNVAHLPPSILDLNYRGTES
GAYCVDQISSNLSTMYTQMVSSATTRDAFFGTNPDPVTASTAGTIERGAHTAVHIWVGN
PRMTNNEDLGNFYSAGYDPAFYLHHANVDRMWKVWKDLSPSTNKDPDNDDWNNAS
YVFYDENKNLVRVYNKDCVDINKMGYDYEDSRIPWIKSRPVARVKASKVAAKSVGVV
KKVEEIKFPVKLDKIVKVLVKRPATNRSESDKKKATEMLFLNGINYNSEQFFKFDVLVD
DVDDGIETTASSSEFAGTFAQVPHGLRGEKMVMRSGAAFGITELLEDIEAEGDEYVLVT
LVPRAGTDDATISEIKIQLVPIA

>ha-XP_021993749.1 polyphenol oxidase I, chloroplastic [Helianthus annuus]

MASLSLSAVPTTPLSSRSSPLFSKTSTHRFNVSCNSSADENPKLILPKSTSLNVDRRNMLL
GLGGLYGAASLTNIGSASAFPVTAPDNIADCVAATDGVRDPDKAIRGTACCPPKLSDSIP
PDYVLPNFPQLRIRPPAQRVDDDYIVKYRAAIAAMRALPDEDPHSWKQQGKIHCAYCN
GGYSLEMNGLPDVKLQIHNSSLFYPFHRWYLYFYERILGKLIGDDTFALPYWNWDNPTG
MMLPAMLAPVFDDRGNPNQLLNPLFDPFREVKHLPPAIVDLQYRGTESGLDCIDQINTN
LSTMYSQMINQTTDTFFGTNPGDDGKGTPGSIENGAHTSVHIWTGNSKMPNREDMGNF
YSAGYDPAFYVHHANVDRMWHVWKGLNPLTNKDPIDDDWLNASYVFYDENRQLVRV
YNRDAVDVTKMGYDFEPSRTPWINNRPVARVKASNVAAQSVGVVKKVEEMEFPVKLD
QTVKVLVKRPSTKRSEDDKKKANEILFLNGIKYNGQQFFKFDVLVDDVDDGIETTAASS
EFAGTFAQVPHGLGGEKMFMTTGRAFVINELLEDIEAEDDEYVLVTLVPRAGADDATVS
EIKIQLVPTE

>ha-XP_021993034.1 polyphenol oxidase I, chloroplastic [Helianthus annuus]

MSSSLPLTSPFTFTNTQLAFKARTNQTQGFRVSCNSAQDDQADKKLILPESQKLVVPSVD
RRNLLVGLGGLYTAANLPGAMAAPITAPDITSICQDAKDGIRNIATALRTTKCCPPSLGK
TVKPFRFPTEKTVRKRWPAHAGTKKQVDDYRRAIKAMRELDDDHPHSFVSQAKIHCAY
CNGGYTQVDSGTGFPDINIQIHNSWLFFPFHRWYLYFYERILGKLINEPEFALPFWKWDE
PAGMPIAEMFLPESPNPLYDELRDPDHIQKRLIDLDYAGTDKDIPDQQQIECNLATVYRD
LVRNGGDTLSFFGGEYVAGDSPVAGTDPSVGSVESGSHTAVHRWVGKRGTPNSEDMG
NFYSAGYDPVFYIHHANVDRMWKLWKDLRLPGHVDPKEDDWLNASYVFYDENEDLV
RVYNKDCVDVRRLKYDFIENSDGPFPWRKSRPPQRRKSAQVASTVDVKTVEKTKFPVR
LDKIVKVSVKRPAVNRSEEDKEKNNEVLLIKGIKYDSGKFVKFDVFVNDKLKEGEVTTP
CDPEYAGGFAQIPHNAMKSMFMTSSARFGLNELLEDTNTEGDEYAMVTLVPRTGCEDL
TVGQIKIELVPIPKA

>ha-XP_021993752.1 polyphenol oxidase I, chloroplastic [Helianthus annuus]

MASLCVSTFPTTSLSPLSPLSSKPPPYRFKVSCNASSDDHPKTSESPNLILPMQNNNVDRR
NLLLGLGGLYGTANMTNIGSAFAYPVTAPDDISDCVPATSLINNLDDVVRGVACCPPHS
PAAFPKPYVLPSFRALRVRSPAHKLTPEYIEKYKAAIAAMKALPDDHPHSWKQQGKIHC

AYCNGAYHQQLSENQKVEVKVHFSSIFYPFHRWYLYFYERILGKLIDDPSFALPYWNW
DNPMGMMLPAMFEAPGPDNDLRTNPLFDPYRNVSHAAPAIVDLQFDDKERGHPCVDQ
VSINLCSLHTQMIRTKRTDDFFGCDPNPKVSECGGSIENGAHTAVHRWVGNPRMANHE
DLGNFYSAGYDPVFYVHHANVDRMWHIWMGLKGNRNKGPRSTDWKNSSYVFYDENE
ELVRVYNRDCVDMKRMGYMYEASSIPWKESPPIPRVKASKVALKSVRSVKKAEETEFP
VKLDKRVNVLVKRPATTRSEDEKEKATEMLVLNGIKFNSEKFFKFDVLVNDVDDGIQTT
ATSSEFVGTFAQVPHMPGHKMFITSGASYGITEVLEDLEAEEEEYVLVTLVPRAGTEDAT
VSEIKIELLPID

>ha-XP_021993036.1 polyphenol oxidase I, chloroplastic-like [Helianthus annuus]

MSSSLLPLTSPFTSTNTQLAFKARTNQTQGFRVSCNSAQDDQADKKLILPESQKLVVPNV
DRRNLLVGLGGLYTAVNLPGAMAAPITAPDITSICKDASAGIRNQEGAIRTRKCCPPSLG
KKIKEFQFPTENSVRKRWPAHAGTPKQVDDYRRAIQAMRDLPDEHPHSFVSQAKIHCA
YCNGGYTQVDSGFPEIDIQIHNSWLFFPFHRWYLYFYERILGKLINEPNFALPYWKWDEP
RGMPIPEMFVPETVNGKPNSLYDVYRDANHIKERIVDLDYDGKDKDIPDFQQVQCNLAT
VYRDLVRNGGDTLSFFGGEYNAGDPPVENSDPSVGSVEAGSHTAVHRWVGDPKQPNQ
EDMGNFYSAGYDPAFYIHHANVDRMWKLWKELRLPGHVEPTDPDWLNASYVFYDEN
EDLVRVYNKDCVDLGKLKYNFIENSKEVFPWRKSRPPQRRRSSQVATTGEVKTVDKVR
FPVRLSEILKVRVKRPAVNRSDEEKEKANEVLLIKGIKYDSGKFVKFDVFVNDKLKEGEI
TTPCDPEYAGGFAQIPHSDMRSMFMKSSARFGLTELLEDTNTEGEEYATVTLVPRVGCD
DLTVGQIKIELVPVRKV

>ha-XP_021993035.1 polyphenol oxidase I, chloroplastic [Helianthus annuus]

MSSLLPISTTLSAFPSTTTQLAFKARTNQTQGFRVSCNHAQDEQNDNKIILPESQKLVLPN
VDRRNLLVGLGSLYTAVNLPAAMAAPITSPDITSICKDANSGIGDIVGAVRTRKCCPPSL
GKTIKPFVFPTETTVRKRWPAHAGTKKQVDDYRRAIQAMRDLPDDHPHSFVSQAKIHC
AYCNGGYTQVDSGFPEIDIQIHNSWLFFPFHRWYLYFYERILGKLINEPSFALPFWKWDD
PAGMPMPEMFVAETVDGQPNSLYDVYRDSRHLPPAIVDLDFDGKDKNIPDQQQRACNL
ATVYRDLVRNGGDTLSFFGGEYVAGDQPVANGAKSVGSVEAGSHTAVHRWVGDTNQ
PNNEDMGNFYSAGYDPVFYCHHANVDRTWKLWKDLGLPGHVEPTSPDWLNASYVFY
DENQELVRVYNKDCVDIGKLKYTFLENSKEVFPWRKSRPARRSKTDQVASTGDVPTVD
QLKFPVSLENILKVRVKRPAVNRSKEDKATANEILLVNGIKFDSDKFVKFDVFVNDKVK
DGVFTTPCDPEYAGGFAQIPHNDMKKMSMSSAGRFGLTELLEDTNTEGEEYATVTLVP
RVGCEDLTIGEISIKLIPLA

>ha-XP_022013106.1 polyphenol oxidase I, chloroplastic [Helianthus annuus]

MSCSIPVSPTTLTKTTFISQRALIKTHKNQTHGFRVTCNVAPNDDHNHLKLILPDIDRRSL
LVGLGGLYTASNFPSLPAALADPITTPDITSSCKEADYGIKNLEKVVRTRKCCPPNIKKTI

KPFVFPTEKTVKVRWPAHNGSKEQVEKYKRAIQAMKDLPEDHPHSFIQQAKIHCAYCN
GGYTQVDSGFPDIDIQIHNSWLFFPYHRWYLYFYERILGKLINDPTFALPYWNWDNPAG
MTIPEIFLNDTRTPNPLFDIYRDARHLPPQLVDLDFGGGERDTTSEIQIACNLSTVYRDLV
RNGADTKSFFGGEYVAGDAPVANGDKSVGSVEAGCHIAIHRWVGDSTQANFEDLGNL
YSAGYDPLFYAHHSNVDRMWTLWKSLGIQGHTEPTDPDWLNASYVFYDENEDLVRVY
NKDCIKMDKLKYKYYEESESVFPWRKSRPAKRNKESQVALTSPGDVKTVNQLKFPVTL
DAILKTRVKRPAVNRTDAEKAKANEVLLINGIKFDGEKYVKFDVFVNDTLKEGEVTTPC
DPEYAGGFAQIPHSGMDKMMMSSAARFGLTELLEDTNTEGEEYATVTLVPRIGCDNLTI
SDIKIALVPI

>ha-KAF5766510.1 putative catechol oxidase [Helianthus annuus]

MLPAQPWKKNQRIQVSERKDNTDAMARACWDKREQVDKYRRAIQAMRDLPEDHPHS
FVNQAKIHCAYCNGGYTQVDNGFPDIEIQIHNSWLFFPFHRWYLYFYERILGKLINDPTF
ALPFWKWDEPVGMPIPEMFLPEVVNGKPNSLYDVYRDEIHIKQRLVDLDYDGNDKDIT
NQTQVLCNLSTVYRDLVRNGADTISFFGGEYKAGNSPIKNGDPSVGSVEAGSHTAVHR
WVGDRKKRNGEDMGNFYSAGYDPVFYIHHSNVDRMWKLWKELGIKGHNEPTDPDWL
NASYVFYDENEELVRVYNKDCVNIRKLNYDFIENSKEVFPWRKSRPARRNKKSQVEPT
ATVETVDKIKFPVRLNEILKVKVKRPAVNRSEAEKAKANEVLVIKKIRYDSGRFVKFDV
FVNDKVKPGEITTPCDPEYAGGFAQLPHNDMKNMFMGSSARFGLTELIEDTNTDGEEY
ATVTLVPRTGCDDLTIGEIKIQLVPILA

>ha-XP_022020421.1 polyphenol oxidase, chloroplastic [Helianthus annuus]

MASLAPPQTTTTTGKNLSSSSTYASSFSFKSSQVPISRNRTKRHAVSCKTLDNHNDDHQE
NSPKIDRRNVMLGLGGLYGAAATFGSGSLAFADPIMAPDITKCGAADLPQGVKPITCCPP
VAKSIIDFKPPPVSTTRIRPAAHLVNKDYIAKFNKAIELMKALPDDDPRSFKQQAAVHCA
YCDGAYDQVGFPDLELQVHGSWLFLPFHRHYLYFFEKICGKLIDDPSFAIPFWNWDAPD
GMTIPKIYTDKKSPLYDTFREARHQPPSIVDLDFNGVDENLSRSKQVSTNLTIMYRQMVS
SAKTASLFMGNPYRAGDEVPGGGSLESIPHGPVHVWTGDSTQPNGEDMGNFYSAGRDP
LFYAHHANVDRMWSVWKTLGGRRADFTDKDWLDSSFLFYDENAEMVRVKVRDCLDS
KNLGYVYQDVETPWLNTKPTPRINRVLNKIKKLGSARADEHIPFAKDVFPTSLNKTIKVL
VPRPKKSRSKKQKDEEEEILVIEGIEVKRDEFVKFDVFVNDEDDGMNATADKTEFAGSF
VNVPHKHHKHGKSAKTRLRFGISELLDDLKANDDDNVLVTLVPKNGAGEISIKGIKIEHE
D

>ha-XP_021989494.1 polyphenol oxidase I, chloroplastic [Helianthus annuus]

MSSLTTSPATATGTTTKTYSSSFTTTSPTVSSWPLFSKTSKKCAIQSLKHKVSCNAGSSEN
LLNNLDRRNVLLGLGGLAGAVNLTSVPSVGAAPLAAPDISKCGTNPLSGFKPGENTPTG
GDCCPPNSTLIKDFEFPTNQAFKVRPAAHLLSAKYIAKFNEAIKRMKALPEDDPRNFLQQ

AHIHCAYCNGAYTQSSSGFPDIEIQIHNSWLFFPFHRWYLYFYERILGSLIDDPTFALPFW
NWDTPAGMTIPKYFNDPKSALFDTKRNQAHLKGVVDLGYNGKDTDATDIEKVKNNLAI
MYRQMVTNATDPTAFFGGEYRAGKEPISGGGSVEQSPHTPVHRWVGDPREPNGENLGN
FYSAGRDTLFYCHHSNVDRMWSLWKMLGGKHKDITDTDWLNTSFVFYDENKNLVRV
YVKDCLLTNQLGYDYQRVDVPWLKSKPVPRAPRSGVAKKLIGKVKKSDDVVFPVKLD
KTVKVLVPRAKKSRSKKEKEEKEEILIIQGITYDSEKYVKFDVYVNDEDDDDDAAPDQT
EFAGSFAQLPHKHKGKTSSKTNFRAGLTELLEELEADDDENVLVTVVPRSGSEDITIDAI
KIIYA

>ha-KAF5756631.1 putative catechol oxidase [Helianthus annuus]

MSTSFLPVSFTLYAVPCTTTQRAFKTRTNQTQGFRVSCNDAPNDHNDNKLIPSESQKLVL
PNVDRGNLLVGLGGLYTVANLQGAIAAPITSPDITSICKEANSDIGNISSSVRTRKCCPPN
LRKTIKPFVFPTENTMRMRWPAHNGTNEQVEKYKKAIQAMRDLPEDHPHSFNSQAKIH
CAYCNGGYTQVDSGFPNVEIQVHGSWLFFPFHRWYLYFYERILGKLINDPTFALPFWKW
DEPAGMSIPEIFVTKVVNGKPNSLYDVYQDTPHLPPAIVDLGFDIVNKGLSNETQIACNL
STPTDPHWLNASYVFYDENEELVRVYNKDCVDIEKLKYDFIKNSKEVLPWRKSRPAKH
SISDQVVSTLEVPTIDELKLPVSLENTLKVRVKRPAVKRSKEENAKANEILLIGGIKFDRD
KFVKFDVFVNEKLKDDVLTTPCDPEYAGGFAQIPHNYMKKMLMSSAARFRLTELLEDT
NTDDEEYATVALVPRSGCEDLTVGEVKIKLVPLV

>ha-XP_022016111.2 polyphenol oxidase A1, chloroplastic-like [Helianthus annuus]

MAGVSTAPATFPMSLDKPATVVVKRPAKNERGHGEEQEEVLVIEGIEVNKDEFVKFDVF
INDEDEEVASGAGPEKTEFAGSFVNVPRKKWEDGGDGSGKVIKTRLRIGISELLEDLGVE
DDDEHVVVKLVPKCDNVHVVIGGVKIEIE

>gj-QBC35993.1 polyphenol oxidase PPO [Gerbera jamesonii]

MASLPTPPATAAGATTKTYSSSFTTTSPAVSSWPVFAQSSKKCSIKSLKHKVSCNAGSSD
NSLNNLDRRNVLLGLGGLAGAVNLTSVPSVGASPLAAPDISKCGTLPLSGFKPGEDTPTG
GDCCPPDSSQILDFEFPKNQTFKVRPAAHLLSPKYIEKFNLAIKRMKELPADDPRNFLQQ
AHIHCAYCNGAYTQSSSGFPDIEIQIHNSWLFFPFHRWYLYFYERIMGSLIDDPTFALPFW
NWDTPAGMTIPKYFNNESSALFDPKRNQSHLQAVIDLGYNGADADTTDVEKVTNNLAI
MYRQMVTNATDPTSFFGGEYRAGNEPISGGGSVEQSPHTPVHRWVGDTREPNGEDLGN
FYSAGRDTLFYCHHSNVDRMWSLWKMLGGKHKDITDEDWLNTSFVFYDEKKNLVRV
YVKDCLFTNQLGYDYQRVDVPWLKSKPVARVPRGGVAKKLAGKIKQTKDVSFPVKLD
KTVKVLVPRPKKSRSKKEKEDKEEILVVQGISYDSEKYVKFDVYVNDEDDVSAPDQT
EFAGSFAQLPHKHKGKSRSKTNFRAGLTELLEELGADDDENVLVTVVPRSGSEDITIDDI
KIILV

>cc-PPO1_V2_02g003610.1.01 2:3791255-3792244

MDFQNSASADYTCVQQIGANLTLMYKQMITNSPNADTFFGGVFRAGDDPIDIQHSTGPI
ESGVHTAVHIWVGNPRMPNNEDMGNFYSAGWDPLFYTHHANVDRMWALWKDXQGP
DYPDHTDPTDPDWLNASYVFYDENKELVRVYNRDCVNVENLSYVYEPSPLPWLRSRPV
PRNTNSNVAAKSFGTVKKVEETKFPVKLDQTVKVLVKRPATKRSKEDKKKSYELLYIN
DIKLDXEKFIKFDVFVNDLDDGTPSTPTDSEFAGSFSQLAHLHGHEMVMRSGASFGLNE
LLEDIQAENDEYILVTLVPGEGCEDVTVGEIKVELVSSAS

>cc-PPO2_V2_02g003620.1.01 2:3811850-3813700

MASLATPTSFTAIPATTPATTTKRLPNSPFLSTSRQRSKQSTHRSFKVSCNATDNNEKXLE
SEKLTLNLDRRNLLLGLGAGGLYSTVNLPAAFADPITTPDIATSCKDSTDGFNLNNHIRT
NACCPPTSLTPVQDYVIPTDEVLRMRPAAHRVTPEYVAKYQAAMAIMRALPXXDPHSF
AQQAKIHCAYCNGGYLQAGYTDKELQIHNCWLFFPFHRWYLYFFERILGKLINDPTFAL
PYWNWDNPSGMSMPGFFEGKYLPNSNSRLPNPAFDAFRNVDHLPPAILNMDFQNSASA
DYTCVQQIGANLTLMYKQMITNSPNADTFFGGVFRAGDDPIDIQHSTGPIESGVHTAVHI
WVGDXRMPNNEDMGNFYSAGWDPLFYTHHANVDRMWALWKDMQGPDYPDHXDPT
DPDWLNASYVFYDENKELVRVYNRDCVNVENLSYVYEPSPLPWLRSRPVPRNTNSNVA
AKSFGTVKKVEETKFPVKLDQTVKVLVKRPATNRSKEDKKKSYELLYVNDIKLDGEKFI
KFDVFVNDLDDGTPSTPTDSEFAGSFSQLAHLHGHEMVMRSGASFGLNELLEDIQAEND
EYILVTLVPGEGCEDVTVGEIKVELVSSAS

>cc-PPO3_V2_02g003630.1.01 2:3822577-3823984

MASLATPTSFTATTPATTTKRLPNSPFFSTSRQRSKQSTHRSFKVSADYTCVQQIGANLTL
MYKQMITNSPNADTFFGGVFRAGDDPIDIQHSTGPIESGVHTAVHIWVGDPRMPNNEDM
GNFYSAGWDPLFYTHHANVDRMWALWKDMQGPDYPDHXDPTDPDWLNASYVFYDE
NKELVRVYNRDCVNVENLSYVYEPSPLPWLRSRPVPRNTNSNVAAKSFGTVKKVEETK
FRVKLDQTVKVLVKRPATKRSKEDKKKSYELLYVNDIKLDGEKFVKFDVLVNDLDDGT
PSTPTDSEFAGSFSQLAHLHGHKMVMRSGATFGLNELLEDIQAENDEYILVTLVPGEGCE
DVTVGEIKVELVSSAX

>cc-PPO4_V2_02g003640.1.01 2:3831196-3844009

MATFATPTTFTGLPATTTKKLPNSPFFSTSXTRSKQTHRTFKVSCNVADENEKRLEAEKP
TLLEGLNLDRRNLLLGLGAGGLYIPAAFAAPITTPDISTCKASVEGFFNLKXRIRTNACCP
PLPLPGIKSVKKHVLPRNEVVRLRPAAHRVTPEYLDKYQRAMAAMRALPDEDPHSFVQ
QAKIHCAYCNGGYFQEGYPDKELQVHNCWLFFPFHRWYLYFFERILGKLINDPTFGLPY
WNWDNPSGMSMPGFFEGKTLPSNDPAITPPLNPAFDAFRNVDHLPPAILDIDYSNTASA

DFTCVQQIGSNLSLMYKQMIANSPNSETFFGGKFIAGDAPISIKNSKGPIESGVHTAAHIW
VGNPRMPNNEDMGNFYSSGWDPLFYTHHANVDRMWKIWKDLKGPDYPDHTEPTDPD
WLNASYVFYDENKELVRVYNKDCVQMENLLYEYEPSPLPWLRSRPVGRNKNSNVAAK
SSGKVKKVEETKFPLKLDGIVKVLVKRPTTNRTKEDKKKAYELLYVNDIKFDGEKFVKF
DVFVNDLDDGTPCSPQDSEFAGSFSQVAHLRGHKMLMTSGVKFGLNELLDDIEAEADE
YILLTLVPREGCQEVTIGEIKIALVPSSYPDKELQVHNCWLFFPFHRWYLYFFERILGKLIN
DPTFGLPYWNWDNPSGMSMPGFFEGKTLPSNDPAISPPLNPAFDAFRNVDHLPPAILDID
YNNTASADFTCVQQIGSNLSLMYKQMITNSPNSETFFGGKFIAGDDPISIQNSKGPMESG
VHTAAHIWVGNPRMPNNEDMGNFYSLGWDPLFYTHHANVDRMWKIWKDLKGPDYPD
HTEPTDPDWLNASYVFYDENKELVRVYNKDCVQMEKFVKFDVFVNDLDDGTPCSPQD
SEFAGSFSQVAHLRGHKMLMTSGVKFGLNELLDDIETEADEYILLTLVPRDGCQEVTIGE
IKIELVPSSANSG

>cc-PPO5_V2_02g003650.1.01 2:3860408-3862336

MGGQACIQKHSSITMASSILPFTSACTQTAATSTNTFLTPSSTFLSNSKPFSHRSSKNQNH
QGFRVSCNVAPADHDDDNNNPKSLIIPATQKLILPNVDRRNLLVGLGGLYTTANFASLPL
ALAEPITAPNISSSCKTAAMGIGNMKDARILGKLIKDPTFALPYWNWDNPTGMVIPAXFE
EGGKGTKDNPNSLFDAYRDGRHLPPEIVDLNYSAETDATCIEQIGINLATMYRQMVSSA
TSDTLFFGGKYVAGDAPVANGAKSIGSIEAGCHTAVHRWVGDSRMPNNEDMGNFYSA
GYDPLFYIHHTNVDRMWKIWKDLGIPGHSEPTSSDWLNASYVFYDENEELVRVYNKDS
VKIENLKYGYERSEIPWLKGRPVPRSKNSKIASKSIGKVKKVQDVKFPVKLDKTVEVLV
KRPATNRTSADKEKTTEVLLINGILFDSEEFVKFDVFVNDKEDGRPSSPSDSEFAGSFSQV
PHSDMNKMLMSSAAKFGITELLEDTEAEGDEFVLVKLVPKVGCEDLTISEIKIELVSNA

>cc-PPO6_V2_08g003420.1.01 8:3768876-3769760

MYRQMVTNATDPTAFFGGEYRAGIEPIQGGGSIEQSPHTPVHRWVGDPREINGENLGNX
YSAGRXTLFYCHHSNVDRMWSLWKMTGGKHKDLTDEDWLNTSFVFYDENKKLVRVY
IKDCLLTTQLGYDYQRVDVPWLNSKPVPRAPKSGIAKKLAGKVKQTKDVSFPVKLDKI
VKVLVPRXKKSRSXKEKEDKEELLIIQGISYDSEKYVKFDVYVNDEDDDASAPDQTEFV
GSFAQLPHKHKGKTXSKTNFRAGLTELLEELGADDDDNVLVTVVPRSGCEDITIDNIKIIF
A

>cc-PPO7_V2_12g010610.1.01 12:16811702-16813483

MSSLAPPPAITTTARRGKTFSSSSRYSSSLFLRSSKVSTARNPNHRHAVSCKTLDDDHHEE
SGKVDRRNVLLGLGGLYGAAATFGSNSLAFADPIMAPDVAKCGPADLPQGAKPTNCCP
PSTSKIIDFKLPPPSNNLRVRPAAHLANKEYIAKFNRAIELMKALPDDDPRSFKQQAAVH
CAYCDGAYDQVGFPDLELQVHGSWLFLPFHRHYLYFFEKICGKLIDDPNFAIPFWNWD
APDGMKIPDIYTNKKSPLYDPLRDAKHQPPSLVDLDFNGVDENLSRSKQVSTNLTIMYR

QMVSSAKTASLFMGSPYRAGDEANPGGGTLESTPHGPVHVWTGDSTQPNGENMGNFY
SAARDPIFYGHHANVDRMWSIWKTLGGRRQDFTDKDWLDSSFLFYNENAEMVRVKVR
DCLDSKKLGYVYQDVETPWLNSKPTPRLKRVLSKIKKLGVARADEQMPPFAKDVFPAS
LDKVIKVLVPRPKKSRSKKQKDEEEEILVIQGIEVKRDVFVKFDVFVNDEDEGMSGADK
TEFAGSFVNVPHKHKHGKNVKTKLRLGISELLEDLDVEDDDNVMVTLVPKNGGGDISIK
GIKIEFD

>cc-PPO8_V2_17g002880.1.01 17:3179409-3181226

MASFSFSTLPTTFSGSPSFSKASSHRFKVSCNAASGDTNHPETTPKLITSLDMTHNVDRRN
LLLGLGGLYGAANFTSLPSAFATPIAAPDNIADCVTASSNLQNPTDVVRGLACCPPALSS
KPEIYTLPVNPVTRIRPAAQRATPEYIEKYKAAIQEMRKLPDDHPHSFKQQAMIHCAYCN
GGYNQEQSGHPELQLQIHNSWLFFPFHRWYLYFYERILGKLINDPTFAIPYWNWDHPTG
MMLPAMFEDADPDTTPKQNPIFDPYRDVTHLPPAILDAEYAGANTGADCVNQISSNLAS
MYRQMITNATDTTSFFGGEFVSGDNPLQADTLIAGSIESGIHTAMHRWVGNPRMANNE
DMGNFYSAGYDPVFYVHHANIDRMWKIWKDLGIPGHVEPTSDDWNNASYVFYDENER
LVRVFNKDSIDIGRMGYDYERSSVPWLQSRPVAHAKRSKAAAQSVGTVKKVEDVEFPV
KLDQVVKVLVKRPATNRTKEDKEKAHEILLLNGIKFDGEKFVKFDVFVNDVDDGIETTA
AESEFAGSFAQLPHGGHSEKMLMTSGARFGITELLEDIEAEDDEYVLVNLVPKIGCDDAT
VSEIKIELVPIVQT

>cc-PPO9_V2_17g002890.1.01 17:3195388-3206783

MASFSSAAVPSAAPATTNKTNTLPPSPLFSKTSSHRFKTHANQTLRFKISCNAAANDDDK
SLNNSEILNLTPKRSPDMQNVDRRNLLLGIGGLYGAANYMTSIPSAFAAPITAPDNISDCI
AANAGISDKKDAARTLACCPPVLSESAPKRFVLPKGAVTRVRPAAQRVTDEYLAKYNE
ALQKMRDLPDDDPRSFKQQAMIHCAYCNGSYRQKEQDGKPRKEIQIHNSWLFFPFHRW
YLYFYERILGKLIDDPKFAIPYWNWDSPTGMTIPAMFEPDPAKGDPRKNPIFNAYRDAK
HLPPVVVDIDYNGKERGASCIDQISINLAAMYKQMISSATDPISFFGGEYKAGDDPVNM
GSPLIGSIESGCHTAVHRWVGNSRMPNNEDMGNFYSAGYDPIFYAHHANVDRMWKIW
KGLGIKGHHDPTSEDWLEASYVFYDENKELVRVYNKDCVDTEPMGYEYETSRIPWSRN
RPIPRTKKPKIAARSAGRVRKVEDVKFPLKVDRIVKVLVKRPARNRSKEEKEKEKEILFM
DGIRFDSETFVKFDVYVDDKDDEPATTAAESEFAGSFAQLPHNHTDKMFITSAARFGLT
ELLEDIEAEDDDSVLANSHRVHPHAKKTGRRFKVPCNAGEDGNDKTLESSEKPKLIKPE
TSFDMQNIDRRNLLLGIGLTTLSSAFAVPNISDSAAASLDVEMRDGVDTSIGLKHGNRPT
PNAKGSKLIPAESAGTVKDVKFPVKLDQTVKVLVKRPAVNRTKEDKEKATEVLMVNG
VRFDGEKFVKFDVFVNDKDNVPATTAADSEFAGSFAQLPHNRSSEKMMMMSGARFGIT
ELLEDLKAEDDEYVVVSLVPRTGCEDVTVSEIKIELVPIV

>cc-PPO10_V2_17g002900.1.01 17:3212811-3214601

MASLPSPVAAAAAGITTPYSSSFATVSSWPLLSKKCSIKSAKQTHRYGVRCNAADSDDQ
KPIIESLNSLDRRNVLLGLGGIAGAVNLTSIPSVGAAPLVAPDISKCGTNPLSGFKAGADT
PEGSDCCPPDSSQIMDFQFPKEEVFRVRPAAHLLSPSYIAKFNLAIKRMKELPADDPRNFL
QQAHIHCAYCNGAYTQSSSGFPDVEIQIHNSWLFFPFHRWYLYFYERILGSLIDDPTFGLP
FWNWDTPAGMKIPSYFNESSSALFDSKRNQDNLTAVVDLGYNGQPSTLSDLDKVTNNL
AIMYKQVVTNATDPLLFFGGEYRAGTEPIRGGGSVEQSPHTPVHRWVGDPRELNGEDL
GNFYSAGRDTLFYCHHSNVDRMWSLWKTLGGKHKDLTDSDWLNTSFVFYDENKNLV
RVYVKDCLLTSRLGYDYQRVDVPWLNSRPVPRSRKSGIARRSMGRVKRTTDINFPVKL
NKTVRVLVPRPKTSRSQTEKENEQEVLVVQGIEYDSQQYVKFDVYVNDEDDDNTSPAQ
TEYAGSFAQLPHTHKGKTKSKTNFRAGLTELLEDLEADDDDTILVTLIPRSGCGDITIDGI
NIVYV

>cc-PPO11_V2_ScYrq3g_1694g000100.1.01 ScYrq3g_1694:2494-2931

MAGISTAEATFPMSLEKPVTVVVARPAKKERDQEEEEEEVLVIEGIEIKRDEFVKFDVFIN
DEDEMPASGGGPEKAEFAGSFVNVPHKQRDGGGGMVIKTRLRLGISELLEGLEVGDDD
EHVLVKLVPRCDDVHVTITGIKIEIE

>dp-QOC60273.1 aurone synthase [Dahlia pinnata]

MSSSLLPLASTISAFPSPNTQSIAFKTRTNQTQGFRVSCNTAPDNTNINHNDNKLILPEPQK
LVLPNVDRRNLLVGLGGLYTTTNLTPPSAFAAPIVTPDITSICKDASAGIKNIKDAIRTRK
CCPPSLGKTIKDYVIPAETTLKKRWPAHKGTPKQIDDYIRAIQAMKDLPQDDPRSFLNQA
KIHCAYCNGSYNQDGTDISLQIHNSWLFFPFHRWYVYFYERILGKLINVPDFALPFWKW
DEPDGMPIPEIFLPKDVNGKVNPLYDEFRDANSVKDRIVDLDFGGKDKDRPAKQQIYCN
LNTVYRDLVRNGGDTLSFFGGEYVAGVIPPDKTPSAGSVEAGSHTAVHRWVGDDKQPN
DEDMGNFYSAGYDPIFYVHHANVDRMWKLWKDLRLPGHVEPTDRDWLDASYVFYDE
NQELVRVYNRDCVDINKLSYKYIENSKEVFPWRKSRPVKRDPSFQVQSTVNVPTVDQVS
FPVSLDNILKVRVKRPRVNRTKEEKKKENEVLVINNVKFDCDKFVKFDVFVNDKLKNG
VFTTPCDPEYAGSFTQIPHSDLAKVSMKSAARFGLNQLLDDTNTEGEEYATVTLVPKTG
CDDLTIGQIFIKLVPRLQASD

>mm-KAD3066360.1 hypothetical protein E3N88_34240 [Mikania micrantha]

MSSSSLLPLISSFTSLPSTNTNRAINTKTNQAQGFRVSCNQTPDNHNDKKLVLPQAQKLVI
PNVDRRNLLVGLGGLYTATNLTSMPSALAAPITAPDITSICKDAGAGVNDKENAVRTLK
CCPPSLGKPIKDFVFPTDKKVRMRWPAHTGTKEQVDKYRAAIQAMRDLPDDHPHSFVN
QAKIHCAYCNGGYTQVDSGFPDIDIQIHNSWLFFPFHRWYLYFYERILGKLIKDPDFALP
FWKWDEPAGMPIPEMFVPETIDGKPNSLYDVYRDANHIKARIVDLDYDGKDKDIPDKQ
QVLCNLSTVYRDLVRNGGDTLSFFGGVYVAGDDPVANGAQSVGSVEAGSHTAVHRWV
GDPNQPNNEDMGNFYSAGYDPAFYIHHSNVDRMWKLWKELSLPGHVDPTETDWLDAS

YVFYDENEDLVRVYNRDSVSLDKLKYNYIENSKEVFPWRNSRPAKRNKSLQTATTSDV
KKVDQIKFPVKLDKILKVSVKRPAVNRSAEEKAKANEVLAIKGIRYDSGKFVKFDVFVN
DKLKDGVFTTPCDPEYAGGFAQIPHSGMKNMFMGSSARFGLTELLEDTNTDGDEYATV
TLVPRTGCEDLTVGNIEIILVPVAAV

>mm-KAD3066726.1 hypothetical protein E3N88_34606 [Mikania micrantha]

MSSSSLLPLTSSFTSLPSTNTHRAIKTRTNQTHGFRVSCSHAPDNNNDNKLILPEAQKLVL
PNVDRRNLLVGLGGLYTATNLTSMPSALAAPITAPDITSICKDASAGIRNKDQAVRTLKC
CPPSLGKPIKDFVFPTDKKVRMRWPAQAGTKEQVDKYRAAIQAMRDLPEDHPHSFVNQ
AKIHCAYCNGGYTQVDSGFPDIDIQIHNSWLFFPFHRWYLYFYERILGKLIKDPDFALPF
WKWDEPAGMPIPEMFVPETIDGKPNSLYDVYRNADHIKDRIVDLDYDGKDKDIPDKQQ
VLCNLSTVYRDLVRNGGDTLSFFGGVYYVAGDDPVGNGDPSVGSVEAGSHTAVHRWVG
DSKQPNGEDMGNFYSAGYDPAFYIHHTNVDRMWKLWKDLRLPGHVDPTEKDWLDAS
YVFYDENEDLVRVYNRDSVSLDKLKYNYIENSKEVFPWRKSRPAKRNKNLQTATTTEV
KKVDQIKFPVKLDKILKVSVKRPAVNRSDEEKAKANEVLVIKGIRYDSGKFVKFDVFVN
DKLKDGVFTTPCDPEYAGGFAQIPHSGMKNMFMGSSARFGLTELLEDTNTDGDEYATV
TLVPRTGCEDLTVGKIEIALVPVAAV

>mm-KAD3066364.1 hypothetical protein E3N88_34244 [Mikania micrantha]

MSSSSLLPLTSSFTSLPSTNTHRAIKTRTNQTQGFRVSCNHAPDNQNDKKLILPEAHKLVL
PNVDRRNLLVGLGGLYTATNLTSMPSALAAPITAPDITSICKDASAGIRNKDQAVRTLKC
CPPSLGKPIKDFVFPTDKKVRMRWPAQAGTKEQVDKYRAAIQAMRDLPEDHPHSFVNQ
AKIHCAYCNGGYTQVDSGFPDIDIQIHNSWLFFPFHRWYLYFYERILGKLIKDPDFALPF
WKWDEPAGMPIPEMFVPETIDGKPNSLYDVYRNADHIKDRIVDLDYDGKDKDIPDKQQ
VLCNLSTVYRDLVRNGGDTLSFFGGVYYVAGDDPVGNGDPSVGSVEAGSHTAVHRWVG
DSKQPNGEDMGNFYSAGYDPAFYIHHTNVDRMWKLWKDLRLPGHVDPTEKDWLDAS
YVFYDENEDLVRVYNRDSVSLDKLKYNYIENSKEVFPWRKSRPAKRNKNLQTATTTEV
KKVDQIKFPVKLDKILKVSVKRPAVNRSDEEKAKANEVLVIKGIRYDSGKFVKFDVFVN
DKLKDGVFTTPCDPEYAGGFAQIPHSGMKNMFMGSSARFGLTELLEDTNTDGDEYATV
TLVPRTGCEDLTVGKIEIALVPVAAV

>mm-KAD3066354.1 hypothetical protein E3N88_34234 [Mikania micrantha]

MSSSSLLPLVSSFTSLPSTNTNRAIKTQTNQAQGFRVSCNQKLVIPNVDRRNLLVGLGGL
YTATNLTSMPSALAAPITTPDITSICKDAGAGVNDKENAVRTLKCCPPSVGKTIKDFVFP
TDNKVRMRWPAHTGTKEQVDKYRAAIQAMRDLPDDHPHSFVSQAKIHCAYCNGGYTQ
VDSGFPDIDIQIHNSWLFFPFHRWYLYFYERILGKLIKDPDFALPFWKWDEPAGMPIPEM
FVPEKIDGKPNSLYDVYRDANHIKARIVDLDYDGKDKDIPDKQQVLCNLSTVYRDLVRN
GGDTLSFFGGVYYVAGDDPVANGAQSVGSVEAGSHTAVHRWVGDPNQPNNEDMGNFY

SAGYDPAFYIHHSNVDRMWKLWKELSLPGHVDPTETDWLNASYVFYDENEDLVRVYN
KDSVSLDKLKYNYIENSKEVFPWRNSRPAKRNKNLQTTTTSEVKKVDQIKFPVKLDKIL
KVSVKRPAVNRSAEEKAKANEVLVIKKIRYDSGKFVKFDVFVNDKLKDGVFTTPCDPE
YAGGFAQIPHGNMKNMFMGSSARFGLTELLVDTNTDGDEYATVTLVPRTGCEDLTVGN
IEIILVPVAAV

>mm-KAD3066728.1 hypothetical protein E3N88_34608 [Mikania micrantha]

MSSSSLLPLTSSFTSLPSTNTHRAIKTRTNQTQGFRVSCNHAPDNQNDKKLILPEAQKLVL
PNVDRRNLLVGLGGLYTATNLTSKPSALALPITTPDITSICKDSCLGFKNAEGAIRSRKCC
PPSLGKTIKDFVFPTDKKVRMRWPAHAGTKEQVGKYRAAIQAMRDLPDDHPHSFASQA
KIHCAYCNGGYTQVDSGFPCNDIQVHNSWLFFPFHRWYLYFYERILGKLINDPDFALPF
WKWDEPAGMPIPEMFVPETIDGKPNSLYDVYRNAKHIKDRIVDLDYYDEDKNIPDKQQ
VLCNLSTVYRDLVRNGGDTLSFFGGVYVAGDDPVENGAKSVGSVEAGCHTAVHLWVG
DSKNPNHEDMGNFYSAGYDPAFYIHHSNVDRMWKLWKELSLSGHVDPTETDWLNASY
VFYDENEDLVRVYNRDSVCLDMLKYNYIENSKEVFPWRNSRPEKRDKNLQVATTGDV
KKVDQIKFPVTLNNIFKVRVKRPAVSRSAEEKAKANEVLVIKGIRYDSEKFVKFDVFVN
DKLKDGVFTTPCDPEYAGGFAQIPHSGMKNMFMESSARFGLTELLEDTHTDGDEYATV
TLVPRTGCEDLTIRKIEIILVRVAAF

>mm-KAD3066729.1 hypothetical protein E3N88_34609 [Mikania micrantha]

MSSSSLLPLISSFTSLPSTNTHRAIKTQTSQAQGFRVSCNQTPDDHNDKKLILPQAQKLVIP
NVDRRNLLVGLGGLYTATNLTSMPSALAAPITAPDITSICKDAGAGVNDKENAVRTLKC
CPPSIGKKIKDFVFPTDKKVRMRWPAHAGTKEQVDKYRAAIQAMRDLPDDHPHSFVSQ
AKIHCAYCNGGYTQVDSGTGFPDIDIQIHNSWLFFPFHRWYLYFYERILGKLIKDPDFAL
PFWKWDEPAGMPIPEMFVPEKIDGKPNALYDVYRNADHIKDRIVDLDYDGTEKNIPDK
QQILCNLSTVYRDLVRNGGDTLSFFGGVYVAGDDPVKNGDPSVGSVEAGSHTAVHRW
VGDPNQPNNEDMGNFYSAGYDPAFYIHHSNVDRMWKLWKELSLPGHVDPTETDWLD
ASYVFYDENEDLVRVYNRDSVSLDKLKYNYIENSKEVFPWRNSRPAKRNKSLQTATTS
EVKKVDQIKFPVKLDKILKVSVKRPAVNRSAEEKAKANEVLAIKGIRYDSGKFVKFDVF
VNDKLKDGVFTTPCDPEYAGGFAQIPHSGMKNMFMGSSARFGLTELLEDTNTDGDEYA
TVTLVPRTGCEDLTVGNIEIILVPVAAV

>mm-KAD3066727.1 hypothetical protein E3N88_34607 [Mikania micrantha]

MSSSSLLPLTSTFTSLPSTNTHQAIKTRTNQTQGFRVSCNRAPDNHNDKKLILPETQKLVL
PNVDRRNLLVGLGGLYTATNLTSMPSALAAPITAPDITSICKDASAGITNQDKAIRSRKC
CPPSLGKTIKDFVFPTDKKVRMRWPAHAGTKEQVDKYRAAIQAMRDLPDDHPHSFVSQ
AKIHCAYCNGGYTQVDSGFADKDIQIHNSWLFFPFHRWYLYFYERILGKLIKDPDFALPF
WKWDEPAGMPIPEMFVPETIDGKPNPLYDVYRSADHIKDRIVDLDYREINGKLDKDIPY

KQQVLCNLSTVYRDLVRNGGDTLSFFGGVYVAGNDPVKNGAQSVGSVEAGSHTAVHR
WVGDSKNPNGEDMGNFYSAGYDPAFYIHHSNVDRMWKLWKDLRLPGHVDPTEPDWL
DASYVFYDENEDLVRVYNRDSVSLDKLKYNYIENSKEVFPWRNSRPAKRNKNLQTATT
TEVKKVDQIKFPVKLDKILKVSVKRPAVNRSAEEKVKANEVLVIKGIRYDSGKFVKFDV
FVNDKLKDGVFTTPCDPEYAGGFAQIPHGGMKNMFMGSSARFGLTELLEDTNTDGDEY
ATVTLVPRTGCDDLTVGKIEIILVSVAAV

>mm-KAD3066363.1 hypothetical protein E3N88_34243 [Mikania micrantha]

MSSSSLIPLTSTFTSLPSTNTNRAIKTRTNQTHGFRVSCNHAPDNQNDKKLILPEAQKLLL
PNVDRRNLLVGLGGLYTATNLTSKPLALAAPITAPDITSICKDASAGIGNQDKALRSRKC
CPPSLGKTIKNFVFPTDKKVRMRWPAHAGTKEQVDKYRAAIQAMRDLPDDHPHSFVSQ
AKIHCAYCNGGYTQVDSGFADKEIQIHNSWLFFPFHRWYLYFYERILGKLIKDPDFALPF
WKWDEPAGMPIPEMFVPETIDGKPNPLYDVYRSADHIKDGIVDLDYREINGKLDKDIPY
KQQVLCNLSTVYRDLVRNGGDTLSFFGGVYVAGNDPVKNGAESVGSVEAGSHTAVHR
WVGDSKNPNGEDMGNFYSAGYDPAFYIHHSNVDRMWKLWKDLRLPGHVDPTEPDWL
DASYVFYDENEDLVRVYNRDSVSLDKLKYNYIENSKEVFPWRNSRPAKRNKNLQTATT
TEVKKVDQIKFPVKLDKILKVGVKRPAVNRSAEEKVKANEVLVIKGIRYDSEKFVKFDV
FVNDKLKDGVFTTPCDPEYAGGFAQIPHGGMKNMFMGSSARFGLTELLEDTNTDGDEY
ATVTLVPRTGCDDLTVGKIEIILVPVAAV

>mm-KAD3066730.1 hypothetical protein E3N88_34610 [Mikania micrantha]

MSSSSLLPLISSFTSLPSTNINQAIKTQTNQAQRFRVSCNQTPDDHNDKKLILPEAQKLVM
PNVDRRNLLVGLGGLYTATNLTTIPSALAAPITAPDITSICKDAGAGVNDKENAIRSLKC
CPPSLGKTIKDFVFPNDKKVRMRWPAHVGTKEQVDKYRAAIQAMRDLPDDHPHSFVSQ
AKIHCAYCNGGYTQVDSGFGDKDIQIHNSWLFFPFHRWYLYFYERILGKLINDPDFALPF
WKWDEPAGMPIPEMFVPETIDGKPNSLYDVYRNADHIKDRIVDLDYYGEDKNIPDKQQ
VLCNLSTVYRDLVRNGGDTLSFFGGVYVAGDDPVENGAKSVGSVEAGSHTSVHLWVG
DKQQPSTPNREDMGNFYSAGYDPAFYIHHSNVDRMWKLWKDLPLPGHVEPTESDWLN
ASYVFYDENEDLVRVYNKDSVSLDKLKYNYIENSKEVFPWRNSRPAKRNKNLQTATTS
EVKKVDQIKFPVKLDKILKVSVKRPAVNRSDEEKATANEVLAIKGIRYDSGKFVKFDVF
VNDKLKDGVFTTPCDPEYAGGFAQIPHGAMKNMFMGSSARFGLTELLVDTNTDGDEY
ATVTLVPRTGCEDLTVGNIEIILVPVAAV

>mm-KAD3066732.1 hypothetical protein E3N88_34612 [Mikania micrantha]

MRWPANHGTKEQVEKYRAAIQAMRDLPDDHPHSFVSQAKIHCAYCNGGYSQVDSGFP
DIDIQIHNSWLFFPFHRWYLYFYERILGKLIKDPEFALPYWKWDEPAGMPIPEMFVPEKI
YCEPNSLYDVYRNADHIKERIVDLDYGGTDRGLPYEQQISSNLCTVYRDLVRNGGDTLS
FFGGVYVAGDSPVENNDPSVGSVESGSHTAVHRWVGDPKQTNGEDMGNFYSAGYDPA

FYIHHANVDRMWKLWKDLRLPGHVEPTEPDWLNASYVFYDENEELVRVCNKDSVNLD
KLKYNYVENPNEVSLWRKSRPVKRNIDLQVVTTDDVEKVDQIKFPVSLNKALKVRVKR
PTVNRSAEEKAKANEVLVINGIRYDTGKFVKFDVFVNDKLKDGLVPTPCDPEYAGGFAE
LPHNDMENMFMKSSARFGLTELLEDTNSDGDEYATVTLVPRTKCDDLTIGEIQIVLVQV
A

>mm-KAD3066351.1 hypothetical protein E3N88_34231 [Mikania micrantha]

MRWPANHGTKEQVEKYRAAIQAMRDLPDDHPHSFVSQAKIHCAYCNGGYSQVDSGFP
DIDIQIHNSWLFFPFHRWYLYFYERILGKLIKDPEFALPYWKWDEPAGMPIPEMFVPEKI
YCEPNSLYDVYRNADHIKERIVDLDYGGTDRGLPYEQQISSNLCTVYRDLVRNGGDTLS
FFGGVYVAGDSPVENNDPSVGSVESGSHTAVHRWVGDPKQTNGEDMGNFYSAGYDPA
FYIHHANVDRMWKLWKDLRLPGHVEPTEPDWLNASYVFYDENEELVRVCNKDSVNLD
KLKYNYVENPNEVSLWRKSRPVKRNIDLQVVTTDDVEKVDQIKFPVSLNKALKVRVKR
PTVSRSAEEKAKANEVLVINGIRYDTGKFVKFDVFVNDKLKDGLVPTPCDPEYAGGFAE
LPHNDMENMFMKSSARFGLTELLEDTNSDGDEYATVTLVPRTKCDDLTIGEIQIVLVQV
A

>mm-KAD3066359.1 hypothetical protein E3N88_34239 [Mikania micrantha]

MSSSSLLPLISSFTSLPSTNFNRTIKTQTNQAQGFRVSCNQTPDDHNDKKLILPQAQKLVIP
NVDRRNLLVGLGGLYTATNLTSMPSALADPIKTPDITSICKEAGAGVNNKEKAIRSLKCC
PPSLGKTIKDFVFPTDKKVRMRWPAHAGTKEQVDKYRAAIQAMRDLPDDHPHSFVSQA
KIHCAYCNGGYTQVESGIGNGDKDIQIHNSWLFFPFHRWYLYFYERILGKLINDPDFALP
FWKWDEPAGMPIPEMFVPETIAGKPNPLYDVYRNADHIKDRIVDLDYGGTDRGLPNEK
QIESNLCTVYRELVRNGADTLSFFGGKYAAGNDPVLIGDKSIGSVERGSHTAVHRWVGD
SKNPNDEDMGNFYSAGYDPAFYIHHSNVDRMWKLWKDLRLSGHVDPTETDWLDASY
VFYDENEDLVRVYNRDSVSLNKLKYNYIENSKEMFPWRNSRPAKRNKNLQVTTNSDV
KKVDQINFPVPLNKIFKVRVKRPAVSRSVEEKEKANEVLVIKRIRYDSGRFVKFDVFVND
KLKDDVVTTTCDPEYAGGFAQIPHGRMKNMFMLSSARFGLTELLEDTNTDDDEYATVT
LVPRTGCGDLTVGKIEIILVPVAAV

>mm-KAD5318067.1 hypothetical protein E3N88_18013 [Mikania micrantha]

MSSSICPPATLTSKPTTTFITTPFTSYLPIKTRTSQTHGFKVACNISPSDHDHNKLILPSIDRR
NLLVGLGGLYTATNLTSFSAALADPITTPDITSSCKEADSGIKDLEKAVRTRQCCPPNIKK
TIKPFVFPNEKTVRVRWPAHNGSTEQVQKYKKAIQAMRDLPDDHPHSFIQQAKIHCAYC
NGGYTQVDNGFPDIEIQIHNSWLFFPYHRWYLYFYERILGKLINDPTFALPYWNWDNPA
GMMIPEIFLDRKDESNPLFDVYRDARHLPPKLVDLDFGTGERNTTNEIQIACNLSTVYRD
LVRNGADTKSFFGGEYVAGDAPVANGAKSVGSVEAGCHIAIHRWVGDSTQANFEDMG
NLYSSGYDPLFYAHHSNVDRMWTLWKALGIQGHTEPTDPDWLNASYVFYDENEDLVR

VYNKDCIKMDKLKYSYHEESKSVFPWRKSRPTKRSKNAQTESNSIKDVKRVEELKFPVT
LDGILKISVKRPAVNRSDEEKAKANEVLLINGIKFDGEKFVKFDVFVNDKLKEGEVTTPC
IKDDVD

>mm-KAD4982380.1 hypothetical protein E3N88_19051 [Mikania micrantha]

MLTMASSLASPPPTTTTTAATGKLFSSSSTYVSSFSFKSSQIPISRNRKNHAVSCKTLDND
HHDENTGKLDRRNLMLGLGGLYGTAATFGSGSLAFADPIMAPDITKCGAADLPEGAKP
TNCCPPLAKKITDFKPLPPSITRVRPAAHLVNKDYIAKFNKAIELMKALPDDDPRSFKQQ
AAVHCAYCDGAYDQVGFPDLELQVHGSWLFLPFHRHYLYFFEKICGKLIGDPNFAIPFW
NWDAPDGMKIPDIYTSKTSPLYDSFRDAQHQSPSIVDLDFNGVDENLSRSKQVSTNLTIM
YRQIVSSAKTASLFMGSPYRAGDEANPGGGSLESIPHGPVHVWTGDRTQPNGEDMGNF
YSAARDPIFYAHHANVDRMWSVWKTLGGKRNDFSDKDWLDSSFLFYDENAEMVRVK
VRDCLDSKNLGYVYQNVETPWLNSKPTPRIKRVLSKIKKLGVARADEHIPFAKDVFPAS
LDKPIKVLVPRPKKSRSKKQKEEEEEILVIQGIEVKRDAFVKFDVFVNDEDDGMNATAD
KTEFAGSFVNVPHKHKHKKNVKTRLRLGISELLEDLNADDDDNVLVTLVPKNGGGEISI
GGIKIEHED

>mm-KAD3066350.1 hypothetical protein E3N88_34230 [Mikania micrantha]

MSSSPLLPLISSFTSLPSTNTHRAIKTQTNQAQGFRVSCNQTPDDLNDKKLILPEAQKLLIP
NVDRRNLLVGLGGLYSTATSLASMPSALAKAIHTPPNACKDASAAPVKRFFLFEEMACG
GLSRLTLKKYSYT

>lsal-CAB4115782.1 unnamed protein product [Lactuca saligna]

MASLSLSTLPTSSPTKKPLFSKTSSHVKQSHRFKVSCNSAANNNEKTVKNSETPKLILPKT
SLEMQNVDRRNLLLGLGGLYGAANLTSIPSAFGTPIAAPDNISDCVTASSNLQNANDAV
RGLACCPPVLSTDKPKDYVLPTNPVLRVRPAAQRATDEYIVKYKAAIQAMKNLPDDHP
HSWKQQAKIHCAYCNGGYNQEQSGFPDIQLQIHNTWLFFPFHRWYLYFYERILGKLIND
PTFALPYWNWDNPTGMVLPAMFETDGKRNPIFDPYRNATHLPPAIFEVGYNGTDSGAT
CIDQISANLSLMYKQMITNAPDTTTFFGGEFVAGDDPLNKEFNVAGSIEAGVHTAAHRW
VGDPRMANSEDMGNFYSAGYDPLFYVHHANVDRMWKIWKDLGIKGHTEPTSTDWLD
ASYVFYDENEELVRVYNRDSVNMTAMGYDYERSEIPWLHSRSVPHTKGANVAAKLVG
IVKKVEDVTFPLKLNETVKVLVKRPTKKRNKKNKEEANEMLFLNKIKFDGEEFVKFDVF
VNDVDDGVETTAAESEFAGSFSQLPHGHKHGTKMSMTSGAAFGLTELLEDIEAEDDDSI
LVTLVPKIGCDDVTVGEIKIKLVPIV

>lsal-CAB4106877.1 unnamed protein product [Lactuca saligna]

MSSFQSLATFTSITTRTLPTSPSNRRSNSYPKQTHRLKVSCNVAPEDNEKLLVVPETQKLI
LPKTSLDTLNVDRRNLLLGLGGLCTTVNLTSIPTAFGRPITAPDISSCRASTDGLDLKNAI
RTNACCPPNLSKKVKDFVFPNDKSLRIRRAAHKAPEDYIEKYKAALKAMRALPDDHPH
SFVSQAKIHCAYCNGGYTQIATGDSDKIIQIHNSWLFFPFHRWYLYFYERILGKLINDPTF
AIPYWNWDNPAGMTLPAFFEDGNNRQEKLQNPAFDAFRNTSHFAPAIVDLDYKGEDSG
APSAKQININLTQMNSQMIRNAHDTRSFFGGRYVAGNDPIPRGDKSIGSIEAGCHTAVHI
WVGNSRTINNEDMGNFYSAGYDPLFYVHHANVDRMWVEWKGLDRRNKEPKDEDWL
NASYVFYDENEELVRVYNKDCVRNDKLRYAYEFSPLPWLKNRPTPRTLKSKIALKSVG
TVKQVEDTKFPLKLDKITKVLVKRPATNRSEEEKEKAVELLLIKDVKYNGGKFVKFDVF
VNDQDDVRASSADESEFAGSFAQVPHGPGDDMLMTSGARFGLTELLEDIQAEDDELILV
TLVPKAGCEEVTVGEIKVELVPLDD

>lsal-CAB4106879.1 unnamed protein product [Lactuca saligna]

MSLPAFFEADGKKNPVFDAFRNVNHVSPEAIVDLDYNGSDSGAPCLQQISTNLGAMYK
QMVSNATDPLSFFGGEFRAGDDPFGNGDPSIGSIEAGCHTAVHRWTGNPRMPNNEDMG
NFYSAGYDPAFYVHHANVDRMWKVWKDLGIKGHTEPTDPDWLNASYVFYDENEELV
RVYNKDCVQTENLKYDFELSPLPWLKNRPVAHTKPETTTKPVEKVKVPDVKFPIKLDKI
QKVLVKRPAKNRSQSEKEKATEQLLIKGIKFNVSKFVKFDVFVNDQDDVPTSSASESEF
AGSFAQLPHHHGGHKKLMTSAARFGLTELLEDIGAEDDEYILVTLVPKVGAEDLTIDEIK
VELVPIV

>lsal-CAB4115785.1 unnamed protein product [Lactuca saligna]

MELNGKDLAPATMIASFIFSTVPSATEVTTNNFSHSSIFSKTSSHRFKYTQENQTHRFKVS
CNKTSDDKYDTLETSLDKKNVDRRNLLLGLGGTLYSAANLTFLPSAFSVPIAAPNVSDC
AIASKGIHNIKDAVRGVACCPPVLTLNSPKNYVFPKETAVRIRPAAQRASDDYIDKYKA
AIKAMRDLPDDHPHSFKQQAKIHCAYCNGSYTQKESGKEYEHLTLQIHNSWLFFPFHR
WYLYFYERILGKLIDDPTFAIPYWNWDNPTGMIIPDLFEKPIQVRERKENPVFDAYRDAR
HLPPALVDIDYNGEDRGVSCIDQITINLSAMYKQMISNASDPTSFFGGRYVAGMDHDDK
NSHGTPSVGSIEAGCHTAVHRWVADPRMPNNEDMGNFYSAGYDPIFYAHHANVDRM
WKIWKELGIRGHREPTDKDWLDASYVFYDENEELVRVYNRDCVDLNKLNYDYETSRIP
WARNRPIPRAKNPQMAARSARMGRSSHDVQFPVKLDGIVKVLVKRPYVNRTKEEKEK
ANEILMLNGICFDSEKFVKFDVYVDDKDDEPETTAADSEFAGSFAQLPHHQSGEKMFMT
SAARFGLTELLEDIEAEDDDSIMVTLVPRTGSDDITISEIKIELVPIV

>lsal-CAB4115778.1 unnamed protein product [Lactuca saligna]

MMASLAFSSLPTSTNTTKPLFPKTSSHVKPFHRFKVSCNAPDDNNDNTFNNSDTRKLILP
KTPLETQNVDRRNLLLGLGGLYGAANLTTIPSAFGIPIAAPDNISDCVAATSNLRNTKDAI
RGLACCPPVLSTNKPMDYVLPSNPVLRVRPAAHKATADYVAKYQQAIQAMKDLPEDH

PHSWKQQGKIHCAYCNGGYNQEQSGYPNLQLQIHNSWLFFPFHRWYLYFYEKILGKLIN
DPTFALPYWNWDNPNGMVIPAMFEQNSKTNSLFDPLRDARHLPPSIFDIEYAGADTNAT
CIDQIGINLSSMYRQMVTNSTDTKRFFGGEFVAGNDPLASEFNVAGTIEAGVHTAAHRW
VGNSRMANSEDMGNFYSAGYDPLFYVHHANVDRMWQIWKDIDRKTHKDPISADWLN
ASYVFYDENENLVRVYNRDCVDINRMGYDYERSAIPWIRSRPTAHAKGANVAAKSAGI
VQKVEDIVFPLKLNKIVKIDVFVNDVDDGIQTTAADSEFAGSFAQLPHNHGDKMFMRSG
AAFGITELLEDIEAEGDDSVVVTLVPRTGCDEVTIGEIKIQLVPIV

>lsal-CAB4098603.1 unnamed protein product [Lactuca saligna]

MASLPTPTVTAAGATTKTYSSSFTTTSPVISSWPLFSKKCVLTKPLKHKISCNAGSSENSL
NNLDRRNVLLGLGGLAGAVNLTSVPSVGAAPISAPDISKCGSTPLTGFRPGDNTPTGGDC
CPPDSTQIMDFKFPKNETFRVRPAAHLLSPKYIAKFNEAIKRMKELPETDPRNFLQQAHI
HCAYCNGAYTQSSSGFPDIEIQIHNSWLFFPFHRWYLYFYERILGSLIDDPTFALPFWNW
DTPAGMTIPKYFNDPKNAVFDPKRNQAHLQGVVDLGYNGKDSDTTDIEKVKNNLAIMY
RQMVTNATDPTAFFGGEYRAGIEPISGGGSVEQSPHTPVHRWVGDPRELNGENLGNFYS
AGRDTLFYCHHSNVDRMWSLWKMQGGKHKDITDPDWLNTSFVFYDENKNLVRVYVK
DCLFTNQLGYDYQRVDVPWLNSKPVPRAPRSGVAKKSIGKVKQAKEVSFPVKLDKIVK
VLVARPKKSRSKREKEDQEELLIVQGITYDSEKYVKFDVYVNDEDDDASAPDQTEFAGS
FAQLPHKHKGKTMSKTNFRAGLTELLEDLEADDDDNVLVTIVPRSGSEDITIDNIKIIYA

>lsal-CAB4110483.1 unnamed protein product [Lactuca saligna]

MASLNSTLATVIPSSPDISKTATKRRLMKTHAKLTHRFQVSCNDHEKPPTTNPQPEKLILP
PETSLNRQNVDRRNLLLGLGGLYTTTNLNSLPPVFANPIKAPSFVLGCTDSLWNMDFES
GVRTGACCPPAAKELEMDYKFPTTGETRIRQPVHRASPEYIQKFKDAMQKMKALPDDD
PCSFKNQAKVHCAYCNNSYTQMASGYPEKVLQVHYSWLFFPFHRWSLYFFERILGDLIE
DKTFGLPYWNWDNPAGMEIPAVFEDEGRSNPLYNSYRNVNHLRPAVIDLDYQMKERN
LSPLDQVQVNLCIMNRQMKRNASDPTSFFGGEYVAGDNPISSVGSIEAGCHTAVHRWV
GDPRTPNDEDLGNFYSAAYDPLFYVHHANVDRMWTLWKGLGGEGRKEPNDKNWEEA
SYVFYDENRKPVRVYNKHCVNLEDLKYEYEDSETPWKSNPPKPRSATYTETTSPENVTE
LEFPLSISETVTVQVKRPETNRPKDQKKTIKEILLLKGINFNGGKFVKFDVFVNTFEDINRI
SPCESQYAGGFGLLPHKNSEKMNSMTGVRIELTELLEEINADGDDSVQVTIVPRVGCDD
VTFEDIKIELIDVYEKESN

>lsal-CAB4091620.1 unnamed protein product [Lactuca saligna]

MASFAPSQATSSRSGRRFSSSSTYSSSFSFKSSQVPIARISKHPHAVSCKTLDNDLHHHTN
SSKLDRRNVLLGLGGLYGAAANFGSNSLAFADPIMGPDISKCGPADLPQGAVPTNCCPP
YNTKIIDFKLPPQSNPLRVRPAAHLVDKDYVDKFSKAIELMKALPDDDPRNFTQQAKVH
CAYCDAAYVQLGYPDVELQVHNSWLFFPFHRYYIYFFEKICGKLIGDPNFAIPFWNWDS

PDGMKIPDIYTDKNSSLYDILRDAKHQPPTLIDLDFFGTDDNLSPSEQTSTNLTIMYRQM
VSNAKTASLFMGSPYRAGDDPSPGPGSLESVPHNPIHLWTGDRNQPNGEDMGNFYSAG
NDPIFFAHHANLDRLWSVWKTLGGRRKDFTDNDWLDSSFLFYDENAELNRVKVRDCV
DSKNMNYIYQDVDLPWIESKPVPRLQRASKNIKKHAHVHIPFAKDVFPASLDNVIKVLV
PRPKKSRTKKQKEEEEEVLVIEGIEVKRDKFVKFDVLVNDEDDGTRATAAETEFAGSFV
NVPHMHKHEKNGKTRLRLGITDLLVDLKAEDDDNVLVTLVPKTSGGDISIGGIKIKHE

>lsal-CAB4091621.1 unnamed protein product [Lactuca saligna]

MASFGSSPTSTLTGTACRSERRISSSSNYSSSFSFKSSQIPIQRISKHRHAISCKTLDDDHHH
HANSGKLDRRNILLGLGGLYGTAATFGSNSPAIADPLAPNLSKCGPADLPEGAIPTDCCP
PYTTKILDFKLPPLSNTLRVRPAAHLANEDYIAKFNKAIELMKALPDDDPRSFKQQANV
HCAYCEGAYDQVGSDLQLQVHNSWLFFPFHRHYMYFFEKICAKLIDDPNFAIPFWNWD
APDGMKIPDIYTNKNSPLYDTLRDAKHQPPSLIDLDFNGVDENLSPSKQTSTNLTIMYRQ
MVSSSKTASLFMGSPYRSGDDPDPGAGSLENTPHNAVHIWAGDWNQKNGENMGNLYS
AARDPIFYAHHANIDRMWSVWKTLGGRRKDFTDNDWLDSSFLFYDENAELNRVKVRD
CLDTKNLGYVYQDVEIPWLKNKPVPRRTKATHKPRHNKRAVARADENIPFAEVVFPAS
LDKVIKVLVPRPKISRSEKQKEEEEEILVIEGIEVKMDEFVKFDVFINDEDDGMTATADKT
EFAGSFVNIPHKHNHGRNLKTRLRLGISELLEDLKAEDDQNVLVTLVPKTKGSGLSIGEI
KIEYEE

>lsal-CAB4106878.1 unnamed protein product [Lactuca saligna]

MLGRQRVTLPNSPSNRRSNSYPKQTHRLKVSCNVAPEENEKLLVVPETQKLILPKTSLD
VNTLNVDRRNLLLGLGDLYTTVNFTSIPAVFAKPITTPNISSCRASTDGFDLKSAIRTNAC
CPPNLSKKVKDFVFLSDKSLRIRRVAHKAPEDYIDKYKAALKAMRALPDDHPHSFVSQA
KIHCAYCNSEYTQIATGNSDKIIQIHNSWLFFPFHRWYLYFYERILGKLINDPTFTIPFWN
WDNPAEMTLLAFFEDGNNRKEKLENPACEAFRNTSYFAIADLNYSGDDSGFPCAQQISI
NLTQMNNQMTRNVHDTRSFFGGKYVAGSDPIPNRDSSIGSIEVVCHTTIHRCVGDSRMN
NKEDKGNFYSAGYDPVFYVHHANINRMWVEWMELVRVYNKDCVCNDKLRYAYKFSP
LTWFKNPTTPRTLKSKIALKSVRTVNQVEDTKFPLKLDKVTKVLVERLATNRSKEEKEK
AVELLMIKGVKYNGGKYVKFDVFVNDQDDVRASSAEESEFAGSFAQLLHSPCDDMLM
TSGARFRLTDLLEDI

>lsal-CAB4115777.1 unnamed protein product [Lactuca saligna]

MAPFNFTLATPTTSSSPFFSQTTTKQRRLKTHGKQTHRFQVSCNVSSNDHEKPPPTNPQP
QKLILPQTSLNLQNVDRRNLLLSLGGVYSAATLSGLPPAFAEPIKAPFNQPDRPCKDAVS
GFDINKKLLRPIDCCPLSKNVEESDFKFPDKSSKTRIRYPLHNLPLGYLDKYMDAIQKMK
DLPDSDPRSFKNQAKVHCAYCNGSYTQNDKELQIHNSWLFFPFHRWYLYFYERILGDLI
HDSTFGLPYWNWDNPEGMTIPHFFVEKQCNNYNVKNGENPLYDEYRDASHLRYELIDL

DYSGKNRDLCYDQKEINLATMNRQMMRNAFDATSFFGGKYVAGDEPIPRGDNVVGSV
EAGCHTAVHRWVGNPTNENKEDMGNFYSAGYDPLFYVHHSNVDRMWTLWKQMGGK
EPTDTDWENASYVFYDEKQNPVRVNNKQSVDLSNLKYEYKSSATPWMDRPPRSRCNR
PGYTKRNNTKDFPYQKDPPEKLTLTNTVRLRVKRPPASKNRSAAQKKSEKEILYFIGISF
DCTEAAKFDVFVNDCDEEQITPCDSENVGSFAAVPHAKGMAMGCKSGMRFSLTELLEE
TKAEGDESIRVTIVPRTPGNKVTIDAIEIRLIPVL

>lsal-CAB4115783.1 unnamed protein product [Lactuca saligna]

MMTSFTLYTVPAATTATTNIIKTNCYQLKTHAKQTHRLKASCNAIPDENNDKTLETSLQ
MKNIDRRNIILQLGGLFVASNMTSVPLAYADAISARSNHSVCAASPLGIQNLGTPVKEH
MENREDVDTNKLGYQYKRSEIPWGRSQPTEYRKDSKFGDKSIGIEKKVWEVEFPVKLN
KTVKVLVKRPAVNRTKEDKQKANEILLVNGVRFDGEKYVKFDVFVNDIDNGTETTPAD
SEFAGSFAQLPHGKTDRMMMMSGVRFGLTELLEDIKAEDDEYVLVKLVPRTGCDDVTV
SEIKIELVPVV

>lsal-CAB4091611.1 unnamed protein product [Lactuca saligna]

MASLAPSPTTTTTTGGRCFSSSSTYSSSFSFKSSQVPIARITNHRHAVSCKGAQDDDDHH
HENSGKFDRRNILLGLGGLYGAATTFGSNSLAYAAPIMAPDLTKCGPADLPQGAVPTNC
CPPYTTKILDFKLPPPSNTFRVRPAAHLANKDYIAKFNKAIELMKALPDDDPRSFKQQAA
VHCAYCDGAYDQVGFPDLELQVHGSWLFLPFHRYYLYFFEKICGKLINDPNFAIPFWN
WDAPDGMKIPDIYTNKKSPLYDALRDAKHQPPSLIDLDYNGDDENLSRSKQTSTNLTIM
YRQMVSSSKTASLFMGSPYRAGDEASPGAGSLESIPHGPVHIWTGDRNQQNGEDMGNF
YSAARDPIFYAHHSNVDRMWSVWKTLGGRRNDFTDKDWLDSSFLFYDENAEMVRVK
VRDCLDSKKLGALSKIKKLVVARADEHIPFAKDVFPASLDKVIKVLVPRPKKSRSKKQK
EDEEEILVIEGIEVKRDEFAKFDVFVNDEDDGMRATADKTEFAGSFC

>lsal-CAB4091612.1 unnamed protein product [Lactuca saligna]

MAGIPATPATFPMSLDKPTTVMVARPAKKEREKEEEEVLVIEGIEINRDEFVKFDVFIND
EDEEAAAGGGAEKAECAGSFVNVPHKHRNGHSGDGGKVKKTQLRIGISELLEDLGVEE
DDEDVVVKLVPRCDNVHVTIGGIKIEIE

>ec-XP_043620775.1 polyphenol oxidase I, chloroplastic-like [Erigeron canadensis]

MSCLLLTSSFTTTNSKPNTFKARTIKTRGLRVSCNVTPTNDDNQIILPETQKLIMPNVDRR
DLLVGLGGLCTAGNLSSLSSALADPIVAPPPDLSCKDASVGNNAKKTLRPLECCPPNLNK
KVKRFVFPNDQEPVRIRWPAHKGTKEQVDKYKKAVAAMRALPDDHPHSFKQQASIHC
AYCNGGYTQALSGYPDTELQIHNSWLFFPFHRWYLYFYERILGKLICDPTFALPYWNWD
NPAGMQIPEMFLYEKCNPKYLGDKCNPLYDEYRDPKHQPPQLVNLQFNGDDKDSDTID

LIKRNLFTVYRDLVRNGGDTISFFGGMYVAGDDPVRNGDPSVGSVEASSHTAVHRWVG
NSNLPNGEDMGNFYSAGYDPLFYVHHANVDRMWKLWKDLRLPGHVEPTECDWLNSS
YVFYDENQDLVRVFNKDSVDIKKLKFDYPKFELPWINSRPPRRDKMSQVALIALGDVKS
LDEVKFPFKLDKILKVLVKRPGVNRTNEDKAKSNENLLINGIKYRGDKFVKFDVFVNDK
VNTDEGKFPTPSDPEYAGGFAELPHGGNESNMLMSSAVRFGLDELLEDTNTEGDEYAV
VTLVPIIGTENLTIQEIKIELVPV

>ec-XP_043620652.1 polyphenol oxidase I, chloroplastic-like [Erigeron canadensis]

MSSSLLHPLTSPFTTSPSKPNTFKARTNGFRVSCNVKPTNDDNNETQKLILPAYNIVDRR
NLLVGLGGLCTAANLSNLSSALADPITAPDITSICKDATSNIKLEGAIRTEKCCPPSLGKTI
KPFVFPKGGEKVRMRWPAHKGTKEQVEKYKQAIAAMRALPDEHPHSFKQQASIHCAY
CNGGYTQVDSGFPDIDIQIHNSWLFFPFHRWYLYFYERILGTLINDPTFALPYWNWDDPA
GMELPEIFLGDESNPLYDVYRDPKHQPPLLVDLAFNGSDKDPDIDQKECNLFAVYRDLV
RNASDTKSFFGGEYVAGDEPIKSGDKSIGSVEAGTHTAVHRWVGDSNRPNGEDMGNFY
SAGYDPVFYCHHANVDRMWKLWKDFGTPGHVEPTKDDWLNASYVFYDENQDLVRV
YNKDSVDTRKLKFDYARSDLGELKWSKSRPPKRNVKSQAATIPLGDVKSLDQVKFPVK
LDKIIKFRVKRPAVNRTDDQKAKSKELLLINGIKFSADKFVKFDVFVNDKVNVGTDKFP
TPCDPEYAGGFAQVPHSERSKMKMTSAARFGLDELLDDTNTEGDEYAVVALVPRTGTQ
DLTIEEIKIELVPVV

>ec-XP_043621955.1 polyphenol oxidase I, chloroplastic-like [Erigeron canadensis]

MSSSLLPLTSSFTTSHSKSKTFKPSTNQTQAFRVSCNVKPTTDNNNENNQKLILPETQNLI
LPVNNVDRRNLLVGLGGLCTAANLSNLSSALAAPITAPDITTICKDAKSGFKIEGAVRTE
KCCPPALGKPVKKFVFPKGEKVRMRWPAHKGTKEQVEKYKQAIAAMRALPDDHPHSF
KQQASIHCAYCNGGYTQVDSGFPDIDIQIHNSWLFFPFHRWYLYFYERILGSLIDDPTFAL
PYWNWDDPAGMEIPKMFLGDESNPLYDVYRDPRHQPPLLVDLAFNGRDKDPDIDQQEC
NLFTVYRDLVRNAGDSTSFFGGDYVAGDAPVKSGSDSIGSVESGSHTAVHRWVGDSSR
PNGEDMGNFYSAGYDPVFYCHHANVDRMWKLWKDLKIPGHVEPTKDDWLNASYVFY
DENQDLVRVYNKDSVEPKKLKFDYAKSDISELSWSKSRPPKRNKKSQAATIPLGDVKSV
EQVKFPIKLDKIIKFRVKRPAVNRTDDEKAKSKELLLINGIKYRGDRFVKFDVFVNDKVN
VGTDKFPTPCDPEYAGGFAQVPHSERSKMLMSSAARFGLDELLDDTNTEGDEYAVVAL
VPRTGTEDLTIKDIKIELVPVV

>ec-XP_043622914.1 polyphenol oxidase I, chloroplastic-like [Erigeron canadensis]

MSSSLLPLTSSFTTCRSKPNTFKARTNQTHGFRVSCNVKPINDDNNETQKLILPNNNVDR
RNLLVGLGGLCTAANLSNLSSALAEPIKAPDITSICRDANKGFDATHALRTAKCCPPSLG
KTIKPFKFPRGEKVRMRWPAHKGTKEQVDKYKRAIDAMRKLPDEHPHSFKSQASIHCA
YCNSGYTQVASGHPELDIQIHNSWLFFPFHRWYLYFYERILGCLIDDPTFALPYWSWDD

PAGMQIPEIFLGDESNPLYDVYRDPNHQPPLLVDLAFDGKDKDPDIDQKECNLFAVYRD
LVRNGGDAISFFGGEYAVGSDTPNSKSAGSMERHCHTAVHRWVGDSVNNRNGEDMGN
FYSAGYDPVFYCHHANVDRMWKLWKDLGIPGHEEPTHEDWLNASYVFYDENQDLVR
VSNKDSVDTRKLKFDYAKSDLSDLLWIKSRPPKRNINSQAATIPLGDVKSLDQVKFPVK
LDKIIKFRVKRPAVNRTDADKAKSKETLLINGIKFNPNNFLKFDVFVNDKVNVGTDEFPT
PCDPEYAGGFAQLPHGVGEHKMLMSSAARFGLDELLEDTNTEGDEYVVVALVPRTGTE
DLTIGEIKIELVPVV

>ec-XP_043629003.1 polyphenol oxidase I, chloroplastic-like [Erigeron canadensis]

MMTSLPTPTVVVAKYTAVTTTSPVVSSWPNMFPMTKGSLFKLPRQSNGKMISCNASSD
KENDQKPMIESSFDRRDMLLGLGGLVGVASLTSARSVGAAPLAAPDISKCGNNPLSGFT
PGENTPQGDDCCPPDSPQILDFEFPKEQPLRVRPAAHLMSPSYIAKFQEAVKRMKELPKD
DPRSFFQQAQVHCAYCNGSYTQSSSGFPDVEIQIHNSWIFYPFHRWYLYFYERILGSLIDD
PTFGLPFWNWDTPAGMRIPSYFNDPNSSLFDSKRNQDHLNAVIDLGYHASNTDTPEQDT
VSNNLAIMYRQMVTNATDPISFFGGEYRAGTEPIDGGGSIEQIPHIPIHVWVGDPRETNGE
DMGNLYSSGRDPLFYCHHCNVDRMWSLWKMLDGKNVDITDTDWLNTSFVFYDENKN
LVRVYVKDCLLTNKLGYDYQKVDVPWVSTKPVPRAPKSGIAKKMTGKIKNTNDTSFPI
KLDKIVKLLVSRPKKSRSKKEKKEHQELLVLYGIKYDSQIYVKFDVFVNDEDDNSSSPSQ
TEYAGSFAQLPHKHKGKSRSKTSFRAGMTELLDELQADDDDSVLVTLIPRTGCDDVTID
GIKIIYV

>ec-XP_043622467.1 polyphenol oxidase I, chloroplastic-like [Erigeron canadensis]

MSCLLLTSSFTTTTHSKPNTFKTRTNKNRGLRVSCNKTPTNDDNQIILPETQKLIMPNVV
DRRDLLVGLGGLCTAGNLSSLSSALAIPISTPDDITSKCQDASIKSKGDIKRTLKCCPPSLG
KTVKPFEFPKGETVRMRWPAHEGTNEQVDKYKKAIQAMRALPDDHPHSFKQQASIHCA
YCNGGYTQVVGNGFPDIKIDVHNSWLFFPFHRWYLYFYERILGKLIDDPTFALPYWNW
DDPSGMQIPKMFLFDENENNPLYDDFRDKRHQPPQLVNLALKPNQDKDLSNDQEKCNL
FTVYRDLVSNANNEIDFFGGKYVAGAGPNDPKPSTGSVERSSHTAVHLWVGDSSLPNRE
DMGNFYSAGYDPLFYVHHANVDRMWKLWKDLRPGENVEPTDNDWLNASYVFYDEN
QDVVRVYNKDSVDIRKLKFDYAKSDINKVAWRKSRPRRRDTNEQAPSLPVVKSVNEET
FPVKLDKILKYRVTRPKRTNDQKAADCKELLSINGIKYRGDKFVKFDVFVNDKVNTKD
DKLPTPCDPEYAGGFAQVPHCESVMFMVSAARFGLDELLEDTNTKGEEYVVVALVPRT
GTEDLTVGEIKIELCPVV

>ec-XP_043626822.1 polyphenol oxidase I, chloroplastic-like [Erigeron canadensis]

MASLQTLTAPTTAETTTKTFSSSFTKTSPPVSSWPLFSKSSKKCTIKSLKHKVSCNANNN
HLDRRDILLGLGGLAGAVNFSVPSVGAAPLAAPDISKCGTNPLTGFRPGEATPTGGDCCP
PDSSQIMDFKFPTDQAFKVRPAAHLLSPKYIAKFNEAIKRMKELPESDPRNFLQQAHIHC

AYCNGAYTQSSSGFPDIEIQIHNSWLFFPFHRWYLYFYERILGSLIGDPTFALPFWNWDTP
AGMTIPKYFNDSKSALFDPKRNQAHLKAVVDLGYNGKDTDATDIEKVKNNLAIMYRQ
MVTNATDPTAFFGGEYRAGIEPISGGGSVEQSPHTPVHRWVGDPRQTNGENLGNFYSAG
RDTLFYCHHSNVDRMWSLWKMLGGKHKDITDPDWLNSSFVFYDENAKLVRVYVKDC
LLTNQLGYDYQRVDVPWLNSKPVPRAPKSGVAKKLDGKVKKTNDVTFPVKLDKIVKV
LVPRPKKSRSKKEKADKEELLIIQGITYDSEKYVKFDVYVNDEDDDDVSAPDQTEFAGS
FAQLPHKHKGKTQAKTNFRAGLTELLEELEADDDDNVLITVIPRSGSEDITIDAIKIIYA

>ec-XP_043626900.1 polyphenol oxidase, chloroplastic-like [Erigeron canadensis]

MASLAPPSTTAKPFSSSSSTYSSSFSFKSSQIPISRENRINRHAIISCKTLDDNNNNNHNENV
NSNKVDRRNMLLGLGGLYGAAATLGSDSLAFAKPIVAPDITKCGAADIPQGAKPMNCC
PPSASKIIDFKIPPPSATRVRPAAHLVSKDYISKFNKAIELMKALPDDDPRSFKQQAAVHC
AYCDGAYDQVGFPDLELQVHGSWLFLPFHRYYLYFFEKICGKLIDDPTFAIPFWNWDAP
DGMKIPDIYTNKKSPLYDSFREARHQPPSIIDLDYNGVDENLSKSKQVSTNLTIMYRQMV
SSAKTASLFMGSPYRAGDEASPGGGSLESIPHGPVHIWTGDSTQPNGEDMGNFYSAGRD
PLFYAHHANVDRMWSVWKTLEGRHQDFTDTDWLDSSFLFYNENAEMVRVKVRDCVD
SKNIGYVYQDVETPWLNSKPVPRVKRVLSKIKKLGSARADDEHHLFAKDVFPTTLYNKP
IKVLVPRPKKSRSKKQKDQVEEILVIEGIEVKRDVFVKFDVLLNDEDEGTSATADKTEFA
GSFVNVPHKHKHGKSVKTKLRLGISELLDDLDADDDENVFVTLVPRNGGDEVSIKGIKIE
LED

>ec-XP_043622604.1 polyphenol oxidase I, chloroplastic-like [Erigeron canadensis]

MSTSLPLITSSVTTTFTARTKKTQGFRVSSCNNVTPTNNDDNDNNQLILPETQKLVLPNN
MNVDRRNLLVGLGGVCTAANLASLSSTLASPITAPDPKCKYSYVDKPGSAIRTLKCCPP
NLGKEVKPFVFPHKKEPVRMRWPAHKGTKEQVEKYKRAIAAMRALPDDHPHSFKQQA
SIHCAYCNGGYTQIESGFPDVELQIHNSWLFFPFHRWYLYFYERILGKLIDDPTFALPYW
NWDDPAGMEIPEIFLGDESNPLYDFFRDPKHLPPRVVDLAYSGKDQDIDPSLDQKECNLI
TMHRDMITNAVDDISFFGGVYVGGSEPVKNGDPSIGSVEAGCHTAMHLWVGRPRSEFP
NGEDMGNFYSAGYDPLFFVHHANVDRMWKLWKDLCLPGHVEPVEDEWLNSSYLFYD
ENEDLIRVYNKDAVDMRKLGFDYSHTDINNVPWRNSRPAKRKEKLPPASGGVHPGKQP
LKLTDKNLKVRVTRPDPPKDDDRKNYKEVLFIDGIKHKGNKLVKFDVFVNDKISADDQ
ALSTCDPEYAGGFAQIAHGSDMAMPCGARFGLDELLEDTNSEDDKVAVVKLVPRTPAE
KITIGNIRIDWVSASIPLADVEKLNSEVFLPVKLGNILKVSIDRPDVIEKRFYKEVLSIKGK
NLDNKAVKFDVFVNDKVNKNSKKEDLPTPCSPEYAGRFARLPLNNGDSSSDFEVTFDL
DKLLRDTKTNHKREVVVALVPKTAETKSLIITDILIKLV

>ec-XP_043618990.1 polyphenol oxidase latent form, chloroplastic-like [Erigeron canadensis]

MSSSIPPLITSSLNTSPFKARTNKTQVFRVSSCKVTSTNDNNDHQLILPETQKLILPNNMN
VDRRNLLVGLGGVCTAANLASLSSAFAAPIEAPDVASVCKVATSGINPDAVKRTQKCCP
PSIPGKDVEPFKFPKEDEEVRMRWPAHKADQYPQQVQWYKDAIKKMRDLPDNHPHSF
VQQASIHCAYCNGGYTQELSGFPDVELQIHNSWLFFPFHRWYLYFYERILGNLIGEPTFA
LPYWNWDDPAGMQIPNIFLGDKSNPLYDEFRNTKHLPPALVDLALGAGNKDQEKCNLI
TVHRDLITNAVDDTSFFGGVYVAGNEPVQNRDPSVGSVEAGSHTAVHVWVGRPRSEFP
NGEDMGNFYSAGYDPLFYVHHTNVDRMWKLWKDLCLPGHVEPAEEDWLNASYVFYD
ENLNLVRVSNRDSVNIQKLKFDYAESNIKTVPWRDSRPDKRNPKSQNVVHASQPLDAP
GKDPLVLKDKNVRVRVGRPERPNDDYTKGYKEILFIDGIAYKNTNKVVSFDVFVNDEV
SADDKALSVCDPEYAGGFAQIAHGDMSMPCGARFGLDDLLANTNYNTKDGKAVVVKL
VPKTNPPEEITIKKIEIKQVPVSFPFADTKILKTEVPLPVTLGNILKVRVDRPQRTDKNKES
KEILIIKGIYPDNKAVKFDVFVNDKVKENTKKEDIPTPCSPEYAGRFARVPLNDDVSSSLI
EAKFELDKLFRDTKTENQKEAVVALLAKTVETRGLSITDIKIELVPIETK

>ec-XP_043625668.1 polyphenol oxidase, chloroplastic-like [Erigeron canadensis]

MAGIPTTQIFPMSLDKPITLVVARPSKNEREKGEEEQEEVLVIEDIEVKNDEYVKFDVFIN
DEDDVAASVGGAGKTEFAGSFVKVPHKQRENREDGDHAKVMMMKTKLRLGISELLES
LGVEDDDENVMVKLVPKCDNVHVMIGGIKIEIE
