# Supplementary material for: Structural and expression analysis of polyphenol oxidases potentially involved in globe artichoke (C. cardunculus var. scolymus L.) tissue browning
Source: Sci Rep. 2023 Jul 29;13:12288. doi: 10.1038/s41598-023-38874-4 (PMC10387078; doi:10.1038/s41598-023-38874-4)
Supplement: Supplementary file 5 — Supplementary Information 5. [file 41598_2023_38874_MOESM5_ESM.pdf]

TITLE

Structural and expression analysis of polyphenol oxidases potentially involved in globe artichoke (C. cardunculus var. scolymus L.) tissue browning

AUTHORS

Valerio Pompili, Elena Mazzocchi, Andrea Moglia, Alberto Acquadro, Cinzia Comino, Giuseppe Leonardo Rotino, Sergio Lanteri

Supplementary Figure S1. Globe artichoke tissue samples collected for gene expression analysis of PPO genes. Pictures showing capitulum sections of Violetto di Toscana varietal type at time zero (T0) and 15 minutes (T15) after cutting. This Supplementary Figure is related to Figure 5 and Figure 6.

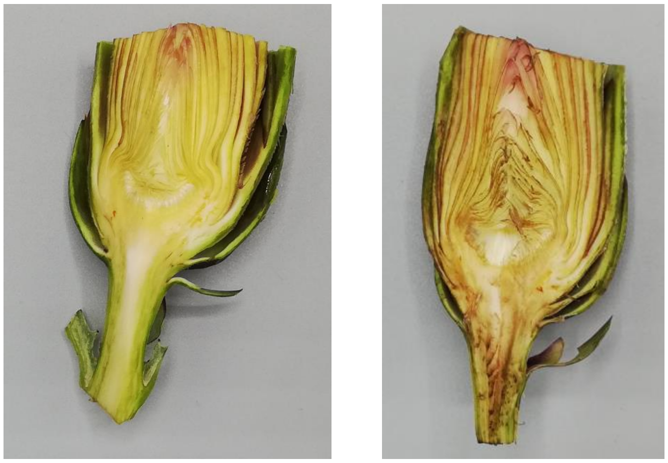

T 0   T 15
