# Supplementary material for: Structural and expression analysis of polyphenol oxidases potentially involved in globe artichoke (C. cardunculus var. scolymus L.) tissue browning
Source: Sci Rep. 2023 Jul 29;13:12288. doi: 10.1038/s41598-023-38874-4 (PMC10387078; doi:10.1038/s41598-023-38874-4)
Supplement: Supplementary file 6 — Supplementary Information 6. [file 41598_2023_38874_MOESM6_ESM.pdf]

TITLE

Structural and expression analysis of polyphenol oxidases potentially involved in globe artichoke (C. cardunculus var. scolymus L.) tissue browning

AUTHORS

Valerio Pompili, Elena Mazzocchi, Andrea Moglia, Alberto Acquadro, Cinzia Comino, Giuseppe Leonardo Rotino, Sergio Lanteri

Supplementary Table S1. List of PPO primers used for gene expression analyses in RTqPCR. Product size and melting temperature (Tm) are respectively indicated as bp and °C. This Supplementary Table is related to Figure 5, 6 and 7.

Gene	Primer	Primer Sequence (5'-3')	Product size	Tm	R^2	Efficiency %
PPO1 (V2_02g003610.1.01)	P Fw<br>P Rev	GATTTCCAAAACTCTGCCAG<br>AACACCAGACTCGATTGGTC	186	62<br>62	0.901	115.668
PPO2 (V2_02g003620.1.01)	P Fw<br>P Rev	GTGCAATGCCACCGATAACA<br>CGATGTCAGGCGTGGTGATA	161	59,19<br>59.90	0.969	111.799
PPO3 (V2_02g003630.1.01)	P Fw<br>P Rev	TTTCTTTTCCACCTCCCGCC<br>CGCAAGTATAATCGGCAGAGAC	77	60,54<br>59,27	0.958	116.906
PPO4 (V2_02g003640.1.01)	P Fw<br>P Rev	GCAGGTAGTTTCTCGCAGGT<br>CGTCCCTTGGTACCAGTGTC	145	60,04<br>60,04	0.981	90.962
PPO5 (V2_02g003650.1.01)	P Fw<br>P Rev	GGGTGGTCAAGCATGCATTC<br>TGGACGTAGCAGCTGTTTGT	98	59,83<br>59,89	0.972	102.467
PPO6 (V2_08g003420.1.01)	P Fw<br>P Rev	TGCCACACAAACACAAGGGT<br>CCGGATCTTGGAACCACTGT	127	60,61<br>59.68	0.985	95.492
PPO7 (V2_12g010610.1.01)	P Fw<br>P Rev	CAAACCATCGCCATGCAGTT<br>TTTCTCCGGTCAACCTTGCC	79	59,76<br>60,54	0.952	95.637
PPO8 (V2_17g002880.1.01)	P Fw<br>P Rev	GCTTCTCCACTCTTCCCACC<br>TTCAGGGTGGTTGGTGTCAC	113	60,04<br>60,11	0.995	103.541
PPO9 (V2_17g002890.1.01)	P Fw<br>P Rev	CTTCTTTTAGCTCGGCCGCC<br>GTTGGTCTTGTTGGTGGTGG	59	61,71<br>59,26	-	-
PPO10 (V2_17g002900.1.01)	P Fw<br>P Rev	GGCTTCTCTTCCTTCTCCGG<br>CGATGGGTTTGCTTTGCTGA	135	59,82<br>59,40	0.992	94.357
PPO11 (V2_ScYrq3g_1694g000100.1.01)	P Fw<br>P Rev	CCACATAAGCAGAGGGACGG<br>CATCATCATCACCCACCTCCA	103	60,18<br>59,51	0.974	117.183
